# New Naphthalene Derivatives from the Mangrove Endophytic Fungus *Daldinia eschscholzii* MCZ-18

**DOI:** 10.3390/md22060242

**Published:** 2024-05-27

**Authors:** Zhiyong Xu, Ting Feng, Zhenchang Wen, Qing Li, Biting Chen, Pinghuai Liu, Jing Xu

**Affiliations:** 1 Collaborative Innovation Center of Ecological Civilization, School of Chemistry and Chemical Engineering, Hainan University, Haikou 570228, China; xuzhiyong@jxas.ac.cn (Z.X.); 20081700210004@hainanu.edu.cn (T.F.); wenzc789@126.com (Z.W.); 22210710000026@hainan.edu.cn (Q.L.); cbt688228@163.com (B.C.); twlph@163.com (P.L.); 2 Institute of Applied Chemistry, Jiangxi Academy of Sciences, Nanchang 330096, China; 3 Institute Research and Utilization on Seaweed Biological Resources, Key Laboratory of Haikou, Hainan University, Haikou 570228, China

**Keywords:** mangrove endophytic fungus, *Daldinia eschscholzii*, naphthalenes, antimicrobial activity, cytotoxicity

## Abstract

Five new naphthalene derivatives dalesconosides A–D, F (**1**–**4**, **6**), a known synthetic analogue named dalesconoside E (**5**), and eighteen known compounds (**7**–**24**) were isolated from *Daldinia eschscholzii* MCZ-18, which is an endophytic fungus obtained from the Chinese mangrove plant *Ceriops tagal*. Differing from previously reported naphthalenes, compounds **1** and **2** were bearing a rare ribofuranoside substituted at C-1 and the 5-methyltetrahydrofuran-2,3-diol moiety, respectively. Their structures were determined by detailed nuclear magnetic resonance (NMR) and mass spectroscopic (MS) analyses, while the absolute configurations were established by theoretical electronic circular dichroism (ECD) calculation. Compounds **1**, **3**, **13**–**17** and **19** showed broad ranges of antimicrobial spectrum against five indicator test microorganisms (*Enterococcus faecalis*, Methicillin-resistant *Staphylococcus aureus*, *Escherichia coli*, *Pseudomonas aeruginosa* and *Candida albicans*); especially, **1**, **16** and **17** were most potent. The variations in structure and attendant biological activities provided fresh insights concerning structure−activity relationships for the naphthalene derivatives.

## 1. Introduction

**Figure 1:**
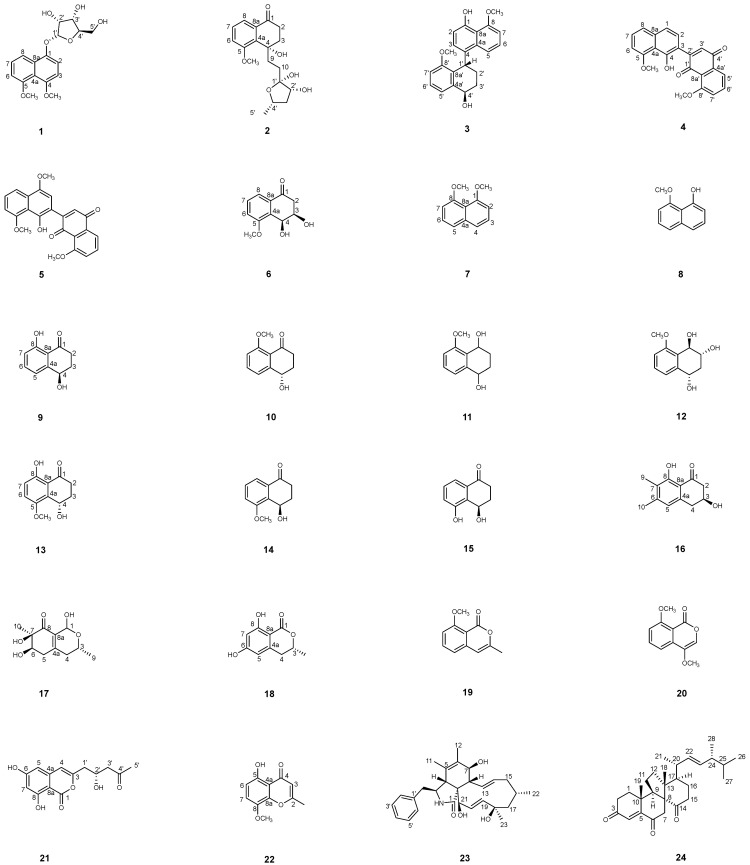
Chemical structures of compounds **1**–**24**.

Microorganisms from special ecological niches such as the mangrove endosymbionts have been demonstrated to be a reliable source of structurally novel and pharmacologically potent natural products (NPs) and have likewise drawn the attention of NP researchers [1,2]. Mangrove-associated fungi produce a larger number of bioactive secondary metabolites compared to any other mangrove-derived microbes; more than 70% were isolated from endophyte fungi [3,4]. Filamentous fungi of the species *Daldinia eschscholtzii* is known as an endophyte commonly found in multiple hosts such as dead trees [5], insects [6], alga [7], broad-leaved forest [8] and mangroves [9,10]. It has been demonstrated to be a prolific source of bioactive polyketides including immunosuppressive dalesconols A and B, acetodalmanols A and B, (+)-daeschol A, dalmanol A, 2,16-dihydroxyl-benzo[j]fluoranthene [6,11], free radical scavengers (±)-daeschol A [11], cytotoxic (±)-acetodalmanol B, 2,16-dihydroxyl-benzo[j]fluoranthene [11], fungistatic helicascolide C [7], stem cell differentiation inducer selesconol [12], NLRP3 inflammasome activation inhibitors spirodalesol [13] and α-glucosidase inhibitors chromones, naphthoquinones, and naphthofurans [14,15]. As part of our ongoing investigations on mangrove endophytic fungi [16,17,18], *Daldinia eschscholtzii*-Mcz18 was isolated from a fresh branch of the mangrove plant *Ceriops tagal*. Five new aromatic polyketide dalesconosides A–D, F (**1**–**4**, **6**), a known synthetic analogue named dalesconoside E (**5**) (this is the first isolation from a natural source), as well as 18 known compounds (**7**–**24**) (Figure 1) were isolated and identified from the mangrove endophytic fungus *Daldinia eschscholzii* MCZ-18. These compounds were examined for antimicrobial and cytotoxic activities. Details of the isolation, structure elucidation, and antimicrobial and cytotoxic activities of these compounds are reported herein.

## 2. Results and Discussion

### 2.1. Structure Elucidation

**Figure 2:**
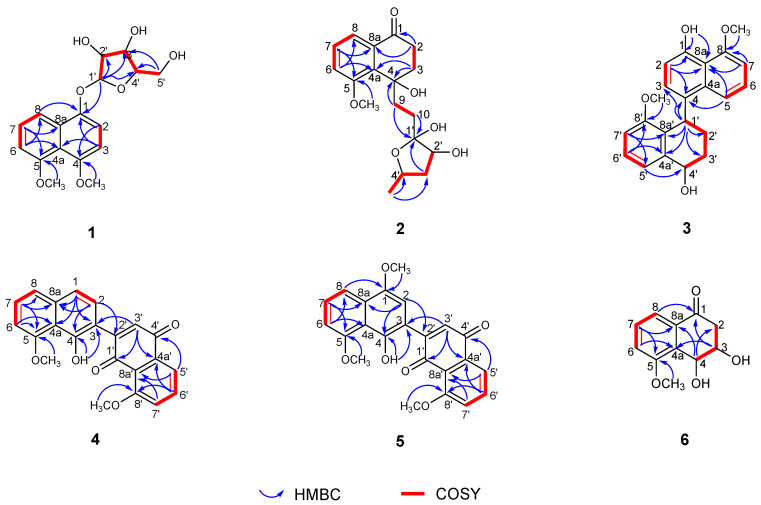
Key COSY and HMBC correlations of compounds **1**–**6**.

**Figure 3:**
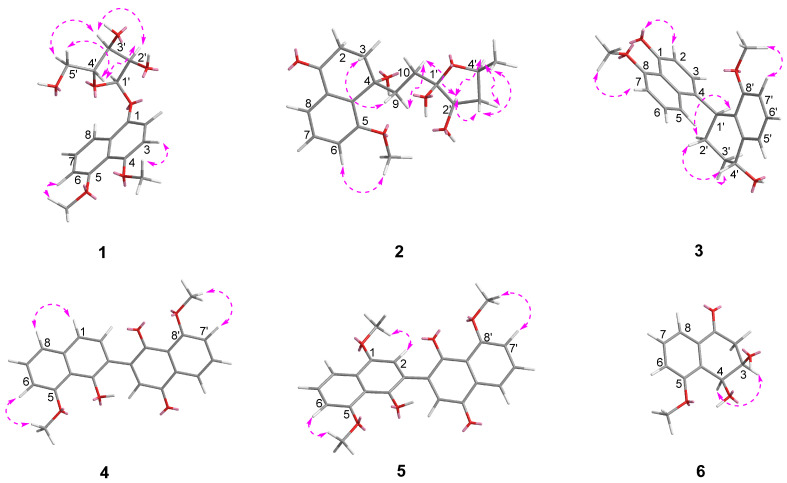
Key NOESY correlations of compounds **1**–**6**.

**Figure 4:**
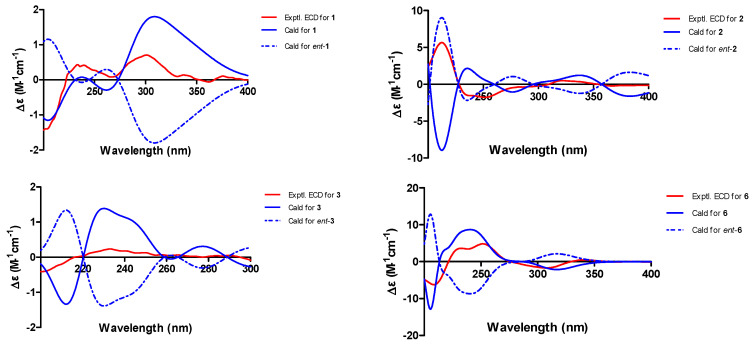
Experimental and calculated electronic circular dichroism (ECD) spectra of **1**–**3** and **6**.

Dalesconoside A (**1**) was isolated as yellow needles with the molecular formula C_17_H_20_O_7_ established by HR-ESIMS (*m*/*z* 337.1289, calcd for [M+H]^+^ 337.1282). Consequently, **1** had eight degrees of unsaturation. The ^1^H and ^13^C NMR data (Table 1 and Table 2) of **1** indicated that seven out of eight degrees of unsaturation are derived from a naphthalene moiety. Therefore, the last unsaturation degree was attributed to an additional ring system. The ^1^H and ^13^C NMR in combination with the ^1^H-^1^H COSY and HSQC spectra showed that the compound had five signals of aromatic protons at [*δ*_H_ 8.00 (d, *J* = 6.4 Hz), *δ*_C_ 116.5, d, CH-8; *δ*_H_ 7.39 (t, *J* = 6.5, 6.4 Hz), δ_C_ 127.2, d, CH-7; 7.18 (d, *J* = 6.8 Hz), δ_C_ 113.1, d, CH-3; *δ*_H_ 6.96 (d, *J* = 6.1 Hz), *δ*_C_ 108.4, d, CH-6; 6.84 (d, *J* = 6.8 Hz), *δ*_C_ 108.2, d, CH-2], an anomeric proton [δ_H_ 5.63 (d, *J* = 3.6 Hz), δ_C_ 103.9, d, CH-1′], two methoxy groups [*δ*_H_ 3.92, s, *δ*_C_ 56.9, q, 5-OCH_3_; *δ*_H_ 3.87, s, *δ*_C_ 57.7, q, 4-OCH_3_], and signals of a furanoside moiety at δ_H_ 3.65–4.24. Comparison of the ^1^H and ^13^C NMR data with those of 1,8-dimethoxynaphthalene (**7**) co-isolated in the culture medium and isotorachrysone-6-*O*-α-D-ribofuranoside [19] revealed that **1** was a 1,8-dimethoxynaphthalene ribofuranoside. The HMBC correlations (Figure 2) observed from H-1′ (*δ*_H_ 5.63) to C-1 (*δ*_C_ 156.6) established that the connection of the furanoside and naphthalene moieties were through the ether bridge. The relative configuration of **1** was interpreted by the diagnostic NOE interactionsH-1′/H-2′, H-2′/H-3′, H-1′/H_2_-5′, and H-3′/ H_2_-5, which revealed their co-facial relationship (Figure 3). The α configuration of ribose was confirmed by the chemical shifts (*δ*_H_ 5.63, *δ*_C_ 103.9, CH-1′) and coupling constant of the anomeric proton *J*_1′,2′_ value 3.6 Hz. The sample limitations precluded further acid hydrolysis to study the absolute configuration of the α-ribofuranoside in **1**, whereas the calculated ECD spectrum method can be used to predict the absolute configuration of C-1′ (Figure 4, Tables S1 and S2). Consequently, the absolute configuration of C-1′ was assigned to be *R*, and the sugar moiety was assigned as α-D-ribofuranoside. Thus, compound **1** was elucidated as 4,5-dimethoxynaphthalen-1-*O*-α-D-ribofuranoside and named dalesconoside A (Figures S1–S8).

Dalesconoside B (**2**) was obtained as a colorless oil, and it possessed the same molecular formula of C_18_H_24_O_6_ as **1** with seven degrees of unsaturation as determined by HRESIMS (*m*/*z* 359.1464, calcd for [M+Na] ^+^359.1465). The ^1^H NMR spectrum of **1** (Table 1) exhibited resonances for three aromatic protons [*δ*_H_ 7.60 (dd, *J* = 7.8, 1.0 Hz), H-8; *δ*_H_ 7.42 (dd, *J* = 8.1, 7.8 Hz), H-7; *δ*_H_ 7.32 (dd, *J* = 8.1, 1.0 Hz), H-6], which indicated the presence of a trisubstituted benzene ring. The ^1^H NMR spectrum further showed signals for 12 aliphatic protons, six oxygenated protons, including two oxygenated methines (δ_H_ 4.15 (t, *J* = 7.5 Hz), H-2′; *δ*_H_ 4.29, m, H-4′), one methoxy group (*δ*_H_ 3.89, s, 5-OCH_3_) and one secondary methyl group [*δ*_H_ 1.21 (d, *J* = 6.3 Hz), H_3_-5′]. The ^13^C NMR spectrum disclosed 18 carbon resonances, including two sp^3^ methyls, five sp^3^ methylenes, two sp^3^ methines, two sp^3^ quaternary carbons (including a dioxysubstituted sp^3^ carbon at δ_C_ 115.8 (C-1′)), three sp^2^ olefinic methines, and four sp^2^ nonprotonated carbons (including one ketone carbonyl), as supported by the DEPT and HSQC spectra. ^1^H–^1^H COSY spectrum (Figure 2) suggested the presence of fragments of –CH_2_(2)–CH_2_(3)–, –CH(5)–CH(6)–CH(7)–, –CH_2_(9)–CH_2_(10)–, and –CH(2′)–CH_2_(3′)–CH(4′)–CH_3_(5′), suggesting that **2** contains a tetralone skeleton and a 5-methyltetrahydrofuran-2,3-diol moiety. This was confirmed by the HMBC correlations from H_2_-2 (*δ*_H_ 2.46, m) and H-7 (*δ*_H_ 7.42) to C-8a (*δ*_C_ 134.7); H_2_-3 (*δ*_H_ 2.76, m; *δ*_H_ 2.63, m) and H-6 (*δ*_H_ 7.32) to C-4a (*δ*_C_ 134.6); and H_2_-3 and H_2_-9 (*δ*_H_ 2.54, m; *δ*_H_ 2.33, m) to C-4 (δ_C_ 84.8). Other correlations in the HMBC spectrum from H_2_-9, H_2_-10 (*δ*_H_ 2.56, m; *δ*_H_ 2.23, m) and H_2_-3′ (*δ*_H_ 2.00, m; *δ*_H_ 1.83, m) to C-1′ (*δ*_C_ 115.8); 5-OCH_3_ (*δ*_H_ 3.89) to C-5 (*δ*_C_ 159.3) further supported the atom connectivity in compound **2**. The relative configuration of **2** was assigned by analysis of the NOESY spectrum (Figure 3). There were observed correlations between H-2′/ H-4′, H-2′/ H_b_-3′, H_a_-3′/ H_3_-5′, H-2′/ H_a_-10, H_a_-10/ H_b_-9, and H_a_-9/ H_b_-3, indicating that H-2′, H_b_-3′, H-4′, H_b_-9 and H_a_-10 are on the same face and H_a_-3′, H_3_-5′, H_a_-9 and H_b_-3 are on the other face, which is consistent with a 4S^*^,1′R^*^,2′S^*^,4′R^*^ relative configuration. The absolute configuration of **2** was then determined by comparing experimental and calculated ECD spectra using time-dependent density-functional theory (TDDFT) as shown in Figure 4, Tables S3 and S4. However, theoretical computations with the expected structure provided an ECD spectrum that was an excellent fit with the mirror image of the measured spectrum of **2**. Finally, the structure of **2** was assigned to be (*R*)-4-(2-((1′*S*,2′*R*,4′*S*)-1′,2′-dihydroxy-4′-methyltetrahydrofuran-1′-yl)ethyl)-4-hydroxy-5-methoxy-3,4-dihydronaphthalen-1(2H)-one (Figures S9–S16).

Dalesconoside C (**3**) was obtained as yellow needles, which has the molecular formula C_22_H_22_O_4_ established by HR-ESIMS (*m*/*z* 351.1597, calcd for [M+H]^+^ 351.1591). Consequently, **3** had 12 degrees of unsaturation. The ^1^H and ^13^C NMR data of **3** (Table 1 and Table 2) indicated that 11 of the 12 units of unsaturation come from a naphthalene moiety and an aromatic ring. Thus, the last remaining degree of unsaturation must be attributed to an additional ring system. The 1D NMR spectroscopic data indicated the presence of a structure with two units, one of which is identical with those of 8-methoxy-1-naphthol (**8**), which was isolated from the same source. The second part showed signals corresponding to 1,2,3,4-tetrahydro-5-methoxynaphthalene with the substitution at C-4. The ^1^H-^1^H COSY and HSQC spectra of **3** allowed the assignments of the fragments –CH(2)–CH(3)–, –CH(5)–CH(6)–CH(7)–, –CH(1′)–CH_2_(2′)–CH_2_(3′)–CH(4′)– and –CH(5′)–CH(6′)–CH(7′)–. The HMBC correlations from H-1′ (*δ*_H_ 5.04) to C-3 (*δ*_C_ 126.6), C-4 (*δ*_C_ 131.5), C-2′ (*δ*_C_ 23.5), C-3′ (*δ*_C_ 26.7), C-4a′ (*δ*_C_ 140.4) and C-8a′ (*δ*_C_ 128.2); from 1-OH (*δ*_H_ 9.37) to C-1 (*δ*_C_ 152.8), C-2 (*δ*_C_ 109.4) and C-8a (*δ*_C_ 115.6); from 8-OCH_3_ (*δ*_H_ 4.08) to C-8 (*δ*_C_ 156.9); and from 8′-OCH_3_ (*δ*_H_ 3.49) to C-8′ (*δ*_C_ 157.2) enable the establishment of the planer structure of **3**. The relative configuration of **3** was based on the NOESY correlations as indicated in Figure 3. The NOESY correlations observed of H-1′/H_b_-2′(*δ*_H_ 1.86), H-1′/H-5 (*δ*_H_ 7.91), H_b_-2′/H-5 and H_b_-2′/H-4′ (*δ*_H_ 4.87) indicated that H-1′ and H-4′ were on the same side of the 1,2,3,4-tetrahydronaphthalene moiety. The absolute configuration of **1** were also determined by comparing experimental and calculated electronic circular dichroism (ECD) spectra for the truncated model (1′*S*, 4′*S*) -**3a** and the truncated model (1′*R*, 4′*R*) -**3b** using time-dependent density-functional theory (TDDFT) (Tables S5 and S6). The calculated electronic circular dichroism (ECD) curve for 1a was in good agreement with the experimental ECD spectrum of **3** (Figure 4). Therefore, the absolute configuration of **3** was firmly assigned as 1′*S*, 4′*S*. Thus, the complete structure of **3** was established (Figures S17–S24).

Dalesconoside D (**4**) was obtained as a red amorphous powder. Its molecular formula, C_22_H_16_O_5_ (15 degrees of unsaturation), was established by HR-ESI-MS (*m*/*z* 361.1071, calcd for [M+H]^+^ 361.1071). Using the ^1^H and ^13^C NMR (Table 1 and Table 2) combined with the HSQC spectra of **4**, a phenolic hydroxyl signal at *δ*_H_ 9.82 (s, 4-OH), the signals for nine aromatic signals at [*δ*_H_ 7.78 (dd, *J* = 7.6, 2.9 Hz), *δ*_C_ 118.8, d, CH-5′; *δ*_H_ 7.68 (t, *J* = 8.2, 7.8 Hz), *δ*_C_ 134.6, d, CH-6′; 7.43 (d, *J* = 7.6 Hz), *δ*_C_ 121.9, d, CH-8; *δ*_H_ 7.37 (t, *J* = 7.8 Hz), *δ*_C_ 126.8, d, CH-7; *δ*_H_ 7.35 (d, *J* = 7.6 Hz), *δ*_C_ 118.7, d, CH-1; *δ*_H_ 7.33, s, *δ*_C_ 128.8, d, CH-2; *δ*_H_ 7.31 (d, *J* = 8.2 Hz), *δ*_C_ 117.8, d, CH-7′; *δ*_H_ 7.06, s, *δ*_C_ 134.5, d, CH-3′; 6.82 (d, *J* = 7.6 Hz), *δ*_C_ 104.6, d, CH-6], and signals of two methoxy groups [*δ*_H_ 4.05, s, *δ*_C_ 56.2, q, 5-OCH_3_; *δ*_H_ 4.01, s, *δ*_C_ 56.5, q, 8′-OCH_3_] were observed. The coupling constants and ^1^H-^1^H COSY spectrum showed the presence of three ^1^H-^1^H spin systems from –CH(1)–CH(2)–, –CH(6)–CH(7)–CH(8)– and –CH(5′)–CH(6′)–CH(7′)–. The NMR spectroscopic data indicated that **4** comprised one 7-hydroxy-1-methoxyanthracene-9,10-dione subunit and one 8-methoxy-1-naphthol (**8**) subunit [20]. The HMBC spectrum (Figure 2) and NOESY correlations (Figure 3) supported the assignments of the methoxy groups 5-OCH_3_ at C-5 (*δ*_C_ 156.7) and 8′-OCH_3_ at C-8′ (*δ*_C_ 159.7). Moreover, HMBC correlations of 4-OH with C-4 (*δ*_C_ 152.8) and C-3(*δ*_C_ 116.2), H-2 with C-3(*δ*_C_ 116.2) and C-2 (*δ*_C_ 150.4), H-2 with C-2′(*δ*_C_ 150.4), and H-3′ with C-3 (*δ*_C_ 116.2) provided evidence for the C-3-C-2′ linkage of **4**. On the basis of the above results, the structure of dalesconoside D (**4**) was identified as 1′-hydroxy-8,8′-dimethoxy-[2,2′-binaphthalene]-1,4-dione (Figures S25–S32).

Dalesconoside E (**5**) was obtained as a red amorphous powder. Its molecular formula C_23_H_18_O_6_ (i.e., differing from that of **4** by an additional OCH_3_ group) was established from HRESIMS at *m*/*z* 391.1177 [M+H]^+^. The ^1^H and ^13^C NMR data (Table 1 and Table 2) of **5** were similar to those of **4** except for the significant downfield shift of C-1(δ_C_ 147.9) and the presence of a methoxy signal (*δ*_H_ 3.96, s, *δ*_C_ 56.0, q, 1-OCH_3_) indicative of **5** was a methoxylated analogue of **4**. The HMBC correlations (Figure 2) of 1-OCH_3_ with C-1 revealed that the extra methoxy group is bound to C-1. Hence, **5** was 1-methoxydalesconoside D (Figures S33–S40). Compound **5** is here reported for the first time as a natural product. It was previously prepared via a two-step sequence involving the oxidative dimerization of hydroquinone monomethyl ethers [21].

Dalesconoside F (**6**) was isolated as a colorless amorphous powder. Its molecular formula C_11_H_12_O_4_ was deduced from HRESIMS at *m*/*z* 209.0806 [M+H]^+^, indicating six degrees of unsaturation. The planer structure of **6** was determined as 3,4-dihydroxy-5-methoxy-3,4-dihydronaphthalen-1(2H)-one [22], which was chemically synthesized from 3,4,5-trihydroxy-1-tetralone with diazomethane, on the basis of ^1^H and ^13^C NMR observations, including 2D ^1^H-^1^H COSY and HSQC and HMBC spectral data (Figure 2). However, the observed NOESY correlations (Figure 3) of H-3 with H-4 suggested that these protons were on the same spatial orientation. Furthermore, the theoretical ECD spectrum (Figure 4, Tables S7 and S8) was also calculated by a quantum chemical method at the [B3LYP/ 6-311+G(2d,p)] level, and the predicted ECD curve of (3*R*,4*S*)-**6** was in good agreement with that of the experimental one. Finally, the absolute configuration of **6**, named dalesconoside F, was unambiguously determined to be 3*R*,4*S* (Figures S41–S48).

By comparing physical and spectroscopic data with those reported in the literature, the known compounds were elucidated as 1,8-dimethoxynaphthalene (**7**), 8-methoxy-1-naphthol (**8**) [23], regiolone (**9**) [24], nodulisporone (**10**) [25], nodulisporol (**11**) [25], xylariol A (**12**) [26], (4*R*)-4,8-dihydroxy- 3-hydro-5-methoxy-1-naphthalenone (**13**) [27], (4R)-O-methylsclerone (**14**) [28], (4*R*)-3,4-dihydro-4,5-dihydroxynaphthalen-1(2H)-one (**15**) [29], (3*S*)-3,8-dihydroxy-6,7-dimethyl-a-tetralone (**16**) [9], fusaraisochromenone (**17**) [30], 3*R*-3,4-dihydro-6,8-dihydroxy-3-methylisocoumarin (**18**) [31], 2-acetyl-7-methoxybenzofuran (**19**) [32], 4,8-dimethoxy-1H-isochromen-1-one (**20**) [22], (+)-citreoisocoumarin (**21**) [33], 5-hydroxy-8-methoxy-2-methyl-4H-1-benzopyran-4-one(**22**) [34], cytochalasin O (**23**) [35], and dankasterone A (**24**) [36].

### 2.2. Biological Activity

Compounds **1**–**24** were tested for antimicrobial activity against *Pseudomonas aeruginosa*, *Enterococcus faecalis*, methicillin-resistant *Staphylococcus aureus* and *Escherichia coli* as well as antifungal activity against *Candida albicans*. As shown in Table 3, all metabolites showed antimicrobial activity against at least one of the five tested indicator pathogens at the selected concentration of 50 μg/mL, but all of the antimicrobial activities were weaker than that of the positive control. Compounds **1**, **3**, **13**–**17** and **19** exhibited a wide range of antimicrobial activities; especially, **1** (against *P. aeruginosa* and *E. coli*), **16** (against *E. coli*) and **17** (against MRSA) were most potent, and their resistant strain with MIC values reached 6.25 µg/mL. The comparison between compounds **1** and **7** approved that the substitution of additional furanose was the critical structural component for its antimicrobial activity. The antibacterial results of the comparison of **2**–**16** suggested that the dihydronaphthalen-1-one nucleus may not be sensitive to the tested microbes in some cases, but the extra auxochromes, e.g., -OCH_3_, -OH or CH_3_ enhanced the antibacterial effect of the compound against the tested bacteria and fungi. Meanwhile, when the hydroxyl group is oxidized to a carbonyl group to a certain extent, the antibacterial activity of the metabolite against some indicator bacteria is weakened. Comparing the antimicrobial activities of compounds **17**–**22** indicated that the *O*-containing heterocyclic substructure may increase the compound’s effect on *P. aeruginosa*, *E. faecalis*, *MRSA* and *E. coli*. When the -OH and -OCH_3_ groups are differently substituted on the benzene ring or the lactone/quionone of metabolites’ isocoumarin backbone, different indicator bacteria have different promotion and inhibition. In general, the broad antimicrobial activities of these naphthalene derivatives support their continued investigation so as to develop a deeper understanding of structure−activity relationships and mechanism of antimicrobial action.

## 3. Materials and Methods

### 3.1. General Experimental Procedure

Optical rotation was recorded at 20 °C using a WYA-2S digital Abbe polarimeter (Shanghai Physico-optical Instrument Factory, Shanghai, China). UV spectral data were obtained from online UV spectra acquired on a Shimadzu UV-2401 PC spectrophotometer. NMR spectra were recorded on a Bruker AV-400 and AV-500 (Bruker Corporation, Fällanden, Switzerland) instrument with TMS as an internal standard. ESI-MS spectra were recorded on a VG Auto Spec-3000 mass spectrometer (VG, Manchester, UK). High-resolution ESI-MS were recorded on an Agilent 6210 mass spectrometer (Agilent Technologies, Waldbronn, Germany) employing peak matching. The ECD spectra were measured on a JASCO J-715 spectra polarimeter (Japan Spectroscopic, Tokyo, Japan). SephadexLH-20 (Beijing Biotopped Science & Technology Co., Ltd., Beijing, China), SiliaSphere C18 (SiliCycle, Quebec, Canada) and Silica gel (200–300 mesh, Qingdao Marine Chemical Factory, Qingdao, China) were used for column chromatography (CC). Thin-layer chromatography (TLC) was performed on precoated silica gel GF254 plates (Qingdao Marine Chemical Factory, Qingdao, China).

### 3.2. Fungal Material

The strain MCZ-18 was isolated from a fresh, healthy branch of *Ceriops tagal* collected from the Dong Zhai Gang-Mangrove Garden on Hainan Island, China. The fungus was isolated under sterile conditions from the inner tissue of the flower following an isolation protocol described previously [37] and identified as *Daldinia eschscholzii* (GenBank accession no. MH712260) by morphologic traits and molecular identification. A voucher strain was deposited in the School of Chemical Engineering and Technology, Hainan University, Haikou, China.

### 3.3. Fermentation, Extraction and Isolation

The *Daldinia eschscholzii* MCZ-18 strain was cultivated on an autoclaved rice solid-substrate medium, consisting of 80 1 L Erlenmeyer flasks. Each flask included 80 g of rice, 0.24 g of sea salt and 80 mL of water. The culture was maintained at a temperature of 25 °C. Following a fermentation period of 29 days, the mycelia and rice were subjected to three extractions using EtOAc. The extracts underwent filtration and evaporation under decreased pressure to yield a crude extract of 75 g. The crude extracts were then submitted to vacuum liquid chromatography (VLC) on silica gel employing a step gradient of petroleum ether/ethyl acetate (1:0–0:1, *v*/*v*) to obtain five fractions (Fr.1–Fr.5). Fr.1 was eluted equivalently with petroleum ether to give **8** (200 mg). Fr.2 was eluted equivalently with petroleum ether / ethyl acetate (50:1, *v*/*v*) to obtain **7** (150 mg). Fr.3 was eluted with a step gradient of petroleum ether/ethyl acetate (20:1, 10:1, 5:1, 3:1, 1:1, *v*/*v*) to give 3 fractions (Fr.3.1–Fr.3.3). Subsequently, Fr.3.1 was further separated by Sephadex LH-20 chromatography (CH_2_Cl_2_/MeOH, 1:1, *v*/*v*) and finally by an ODS column eluting with MeOH-H_2_O (70:30, 80:20, 90:10, 100:0, *v*/*v*) giving **24** (4.5 mg) and **22** (20 mg). Fr.3.2 was eluted equivalently with petroleum ether/ethyl acetate (5:1, *v*/*v*) to give 3 subfractions (Fr.3.2.1–Fr.3.2.3). Fr.3.2.2 was further purified by preparative TLC (developing solvents: CH_2_Cl_2_/MeOH, 100: 3, *v*/*v*) to obtain **14** (12 mg) and **16** (24 mg). Fr.3.2.3 was purified by Sephadex LH-20 CC (CH_2_Cl_2_/MeOH, 1:1, *v*/*v*) and finally by an ODS column eluting with MeOH-H_2_O (60:40, 70:30, *v*/*v*) to obtain **3** (2 mg), **19** (6 mg), **11** (1.0 mg), **5** (2.6 mg), **4** (1.2 mg) and **9** (2 mg). **2** (1.8 mg) and **6** (2.2 mg) was acquired by Sephadex LH-20 chromatography (CH_2_Cl_2_/MeOH, 1:1, *v*/*v*) and C-18 ODS column (MeOH/H_2_O, 60:40, 70:30, *v*/*v*). Fr.3.3 was eluted with a step gradient of petroleum ether/ethyl acetate (3:1, 2:1, 1:1, *v*/*v*) to give 3 fractions (Fr.3.3.1–Fr.3.3.3). Fr.3.3.2 was purified by Sephadex LH-20 CC (CH_2_Cl_2_/MeOH, 1:1, *v*/*v*) and finally by an ODS column eluting with MeOH-H_2_O (60:40, *v*/*v*) to obtain **13** (3 mg), **18** (3.2 mg) and **12** (2 mg). Fr.3.3.3 was purified by Sephadex LH-20 CC (MeOH) and finally by an ODS column eluting with MeOH-H_2_O (45:55, *v*/*v*) to obtain **21** (3.3 mg), **10** (3.6 mg) and **17** (4.4 mg). Fr.4 was purified by an ODS column eluting with MeOH-H_2_O (40:60, 50:50, 60:40, 70:30, 80:20, 90:10, 100:0, *v*/*v*) to obtain 3 subfractions (Fr 4.1–Fr 4.3) and **23** (3.8 mg). Fr.4.2 was purified by Sephadex LH-20 CC (MeOH) and finally by an ODS column eluting with MeOH-H_2_O (45:55, *v*/*v*) to obtain **15** (3 mg), **20** (2.6 mg) and **1** (5.5 mg).

Dalesconoside A (**1**): yellow needles; [α]^20^_D_ +100 (*c* 0.0001, MeOH); UV (MeOH) λ_max_ 226 nm; ^1^H and ^13^C NMR data, see Table 1 and Table 2; (+)-HRESIMS at *m*/*z* 337.1289 [M+H]^+^ (calcd for C_17_H_21_O_7_ 337.1282). 

Dalesconoside B (**2**): colorless oil; [α]^20^_D_ +10 (*c* 0.0001, MeOH); UV (MeOH) λ_max_ 205 nm; ^1^H and ^13^C NMR data, see Table 1 and Table 2; (+)-HRESIMS at *m*/*z* 359.1464 [M+Na] ^+^ (calcd for C_18_H_24_O_6_Na 359.1465).

Alesconoside C (**3**): yellow needles; [α]^20^_D_ +80 (*c* 0.0001, MeOH); UV (MeOH) λ_max_ 202 nm; ^1^H and ^13^C NMR data, see Table 1 and Table 2; (+)-HRESIMS at *m*/*z* 351.1597 [M+H]^+^ (calcd for C_22_H_23_O_4_ 351.1591).

Dalesconoside D (**4**): red amorphous solid; UV (MeOH) λ_max_ 201 nm; ^1^H and ^13^C NMR data, see Table 1 and Table 2; (+)-HRESIMS at *m*/*z* 361.1071 [M+H]^+^ (calcd for C_22_H_17_O_5_ 361.1071).

Dalesconoside E (**5**): red amorphous solid; UV (MeOH) λ_max_ 202 nm; [α]^20^_D_ +70 (*c* 0.0001, MeOH); UV (MeOH) λ_max_ 201 nm; ^1^H and ^13^C NMR data, see Table 1 and Table 2; (+)-HRESIMS at *m*/*z* 391.1177 [M+H]^+^ (calcd for C_23_H_19_O_6_ 391.1176).

Dalesconoside F (**6**): colorless solid; [α]^20^_D_ +80 (*c* 0.0001, MeOH); UV (MeOH) λ_max_ 221 nm; ^1^H and ^13^C NMR data, see Table 1 and Table 2; (+)-HRESIMS at *m*/*z* 209.0806 [M+H]^+^ (calcd for C_11_H_13_O_4_ 209.0808).

### 3.4. ECD Calculations

The Monte Carlo conformational searches were carried out by means of the Spartan’s 14 software (v1.1.4) using a Merck Molecular Force Field (MMFF). The conformers of **1**–**3** and **6** with a Boltzmann population of over 5% were chosen for ECD calculations, and then the conformers were initially optimized at B3LYP/6-31g (d, p) in gas. The theoretical calculation of ECD was conducted in MeOH using time-dependent density functional theory (TD-DFT) at the B3LYP/6-31+g (d, p) level for all conformers of compounds. Rotatory strengths for a total of 30 excited states were calculated. ECD spectra were generated using the program SpecDis 1.6 (University of Würzburg, Würzburg, Germany) and GraphPad Prism 5 (University of California San Diego, CA, USA) from dipole-length rotational strengths by applying Gaussian band shapes with sigma = 0.3 eV.

### 3.5. Antimicrobial Activity Assay

The microplate assay method was used to assess the antimicrobial activities of compounds **1**–**24** against four terrestrial pathogenic bacteria (*Pseudomonas aeruginosa*, *Methicillin-resistant Staphylococcus aureus*, *Bacillus subtilis* and *Escherichia coli*) and one pathogenic fungus (*Candida albicans*) (Guangdong Microbial Culture Collection Center, China, CDMCC) according to previously reported methods [38]. Ciprofloxacin and amphotericin B were used as a positive control.

## 4. Conclusions

In conclusion, five new naphthalene derivatives (**1**–**4**, **6**), a new natural product (**5**) and eighteen known compounds (**7**–**24**) were isolated from mangrove endophytic fungus *Daldinia eschscholzii* MCZ-18. Compounds **1** and **2** are naphthalenes bearing a rare ribofuranoside substituted at C-1 and the 5-methyltetrahydrofuran-2,3-diol moiety, respectively. This is the first report of these naphthalene subtypes being isolated from mangrove-derived fungal sources. All the isolated metabolites showed more or less antimicrobial activities. Several naphthalene derivatives (**1**, **3**, **13**–**17** and **19**) showed broad ranges of antimicrobial spectrum. Based on the structure–activity relationship analysis, oxygen heterocyclic rings such as ribofuranoside and tetrahydropyran moieties, auxochromes, e.g., -OCH_3_, -OH or CH_3_ were considered as the pharmacophores. The results presented herein reinforce the importance of the mangrove microbial environment as a source of antimicrobial compounds with remarkably varied applications.

## Figures and Tables

**Table 1:** ^1^HNMR data of **1**–**6**.

Position	1 ^a,d^	2 ^a,c^	3 ^b,c^	4 ^b,c^	5 ^b,c^	6 ^a,c^
1				7.33, d (7.6)		
2	6.84, d (6.8)	2.46, m	6.59, d (8.0)	7.35, d (7.6)	6.72, s	H_a_ 3.18, dd (16.9, 2.8)
						H_b_ 2.64, dd (16.9, 3.2)
3	7.18, d (6.8)	H_a_ 2.76, m	6.37, d (8.0)			4.37, dd (3.2, 2.8)
		H_b_ 2.63, m				
4						5.13, d (2.8)
5			7.91, d (8.7)			
6	6.96, d (6.1)	7.32, dd (8.1, 1.0)	7.45, t (8.4, 7.7)	6.82, d (7.6)	6.87, d (7.7)	7.29, d (8.1)
7	7.39, t (6.5, 6.4)	7.42, t (8.1, 7.8)	6.86, d (7.7)	7.37, t (7.8)	7.39, t (8.3, 8.0)	7.42, t (8.1, 7.8)
8	8.00, d (6.4)	7.60, dd (7.8, 1.0)		7.43, d (7.6)	7.87, d (7.9)	7.56, d (7.8)
9		H_a_ 2.54, m				
		H_b_ 2.33, m				
10		H_a_ 2.56, m				
		H_b_ 2.23, m				
1′	5.63, d (3.6)					
2′	4.24, m (overlap)	4.15, t (7.5)	H_a_ 2.38, m; H_b_ 1.86, m			
3′	4.15, dd (6.6, 3.1)	H_a_ 2.00,m	H_a_ 1.83, m; H_b_ 1.78, m	7.06, s	7.05, s	
		H_b_ 1.83, m				
4′	4.23, m (overlap)	4.29, m	4.87, t (2.7)			
5′	H_a_ 3.71, dd (9.7, 3.8) H_b_ 3.65, dd (9.7, 3.2)	1.21, d (6.3)	7.11, d (7.6)			
6′			7.32, t (7.9)	7.78, dd (7.6, 2.9)	7.77, dd (7.6, 1.0)	
7′			6.79, d (8.0)	7.68, t (8.2, 7.8)	7.68, t (8.3, 7.7)	
8′				7.31, d (8.2)	7.32, d (8.1)	
1-OCH_3_					3.96, s	
4-OCH_3_	3.87, s					
5-OCH_3_	3.92, s	3.89, s		4.05, s	4.03, s	3.93, s
8-OCH_3_			4.08, s			
8′-OCH_3_			3.49, s	4.01, s	4.01, s	
4-OH				9.82, s	9.42, s	

^a^ In CD_3_OD. ^b^ In CDCl_3_.^c 1^H (400 MHz). ^d 1^H (500 MHz).

**Table 2:** ^13^C NMR data of **1**–**6**.

Position	1 ^a,d^	2 ^a,c^	3 ^b,c^	4 ^b,c^	5 ^b,c^	6 ^a,c^
1	148.6, C	199.8, C	152.8, C	118.7, CH	147.9, C	198.9, C
2	108.2, CH	38.6, CH_2_	109.4, CH	128.8, CH	107.2, CH	41.8, CH_2_
3	113.1, CH	36.6, CH_2_	126.6, CH	116.2, C	114.9, C	71.4, CH
4	153.6, C	84.8, C	131.5, C	152.8, C	146.4, C	65.1, CH
4a	131.7, C	134.6, C	134.1, C	114.8, C	115.4, C	131.2, C
5	158.0, C	159.3, C	117.9, CH	156.7, C	156.5, C	160.1, C
6	108.4, CH	118.6, CH	125.5, CH	104.6, CH	105.6, CH	117.3, CH
7	127.2, CH	130.3, CH	103.8, CH	126.8, CH	126.4, CH	130.4, CH
8	116.5, CH	120.1, CH	156.9, C	121.9, CH	116.0, CH	118.7, CH
8a	119.7, C	134.7, C	115.6, C	137.4, C	129.0, C	133.8, C
9		36.1, CH_2_				
10		35.1, CH_2_				
1′	103.9, CH	115.8, C	33.7, CH	183.3, C	183.3, C	
2′	73.7, CH	75.0, CH	23.5, CH_2_	150.4, C	151.0, C	
3′	71.2, CH	40.4, CH_2_	26.7, CH_2_	134.5, CH	134.5, CH	
4′	87.6, CH	73.3, CH	67.3, CH	185.4, C	185.4, C	
4a′			140.4, C	134.5, C	134.4, C	
5′	63.3, CH_2_	22.4, CH_3_	121.6, CH	118.7, CH	118.7, CH	
6′			127.2, CH	134.6, CH	134.5, CH	
7′			110.2, CH	117.8, CH	117.8, CH	
8′			157.2, C	159.7, C	159.7, C	
8a′			128.2, C	121.0, C	121.2, C	
1-OCH_3_					56.0, CH_3_	
4-OCH_3_	57.7, CH_3_					
5-OCH_3_	56.9, CH_3_	56.1, CH_3_		56.2, CH_3_	56.2, CH_3_	56.5, CH_3_
8-OCH_3_			56.2, CH_3_			
8′-OCH_3_			55.6, CH_3_	56.5, CH_3_	56.5, CH_3_	

^a^ In CD_3_OD. ^b^ In CDCl_3_.^c 13^C (100 MHz). ^d 13^C (125 MHz).

**Table 3:** Antimicrobial activities of isolated compounds **1**–**24**.

	MIC(μg/mL)				
Compound	*P. aeruginosa*	*E. faecalis*	MRSA	*E.coli*	*C. albicans*
**1**	6.25	25	12.5	6.25	25
**2**	50	>50	50	>50	>50
**3**	25	25	25	12.5	>50
**4**	25	50	50	>50	>50
**5**	12.5	50	25	>50	50
**6**	>50	>50	50	50	>50
**7**	12.5	50	25	25	50
**8**	25	>50	25	50	12.5
**9**	>50	25	>50	>50	25
**10**	50	>50	50	50	>50
**11**	50	25	50	50	>50
**12**	25	25	25	>50	25
**13**	25	12.5	12.5	25	25
**14**	25	25	25	25	25
**15**	50	25	50	25	25
**16**	12.5	25	12.5	6.25	50
**17**	12.5	12.5	6.25	12.5	25
**18**	>50	>50	12.5	25	>50
**19**	12.5	25	25	12.5	>50
**20**	>50	>50	50	50	>50
**21**	50	>50	50	50	>50
**22**	>50	>50	25	50	>50
**23**	>50	>50	25	25	>50
**24**	50	>50	>50	>50	>50
Ciprofloxacin	0.78	0.625	0.3125	0.625	
Amphotericin B					0.78

## Data Availability

The original data presented in the study are included in the article/Supplementary Material; further inquiries can be directed to the corresponding author.

## Supplementary Materials

The following supporting information can be downloaded at https://www.mdpi.com/article/10.3390/md22060242/s1, Figures S1–S48; Tables S1–S8. The 1D, 2D NMR and HRESIMS spectra for **1**–**24** are available online at Figures S1–S48.
