# Supplementary material for: New Naphthalene Derivatives from the Mangrove Endophytic Fungus Daldinia eschscholzii MCZ-18
Source: Mar Drugs. 2024 May 27;22(6):242. doi: 10.3390/md22060242 (PMC11204397; doi:10.3390/md22060242)
Supplement: Supplementary file 1 [file marinedrugs-22-00242-s001.zip › marinedrugs-3007896-supplementary.pdf]

Supporting Information

Article

New Naphthalene Derivatives from the Mangrove Endophytic Fungus *Daldinia eschscholzii* MCZ-18

Zhiyong Xu [1,2], Ting Feng [1], Zhenchang Wen [1], Qing Li [1], Biting Chen [1], Pinghuai Liu [1,3] and Jing Xu [1,*]

[1] Collaborative Innovation Center of Ecological Civilization, School of Chemistry and Chemical Engineering, Hainan University, Haikou 570228, China; xuzhiyong@jxas.ac.cn (Z.X.); 20081700210004@hainanu.edu.cn (T.F.); wenzc789@126.com (Z.W.); 22210710000026@hainan.edu.cn (Q.L.); cbt688228@163.com (B.C.); twlph@163.com (P.L.)

[2] Institute of Applied Chemistry, Jiangxi Academy of Sciences, Nanchang 330096, China

[3] Institute Research and Utilization on Seaweed Biological Resources Key Laboratory of Haikou, Hainan University, Haikou 570228, China

[*] Correspondence: happyjing3@163.com; Tel.: +86-898-6627-9226; Fax: +86-898-6627-9010

Contents

Figure S1. *^{1}H-NMR of compound **1** 4*

Figure S2. *^{13}C-NMR of compound **1** 4*

Figure S3. *DEPT of compound **1** 5*

Figure S4. *^{1}H-^{1}H COSY of compound **1** 5*

Figure S5. *HSQC of compound **1** 6*

Figure S6. *HMBC of compound **1** 6*

Figure S7. *NOSEY of compound **1** 7*

Figure S8. *HR-ESI-MS of compound **1** 7*

Figure S9. *^{1}H-NMR of compound **2** 8*

Figure S10. *^{13}C-NMR of compound **2** 8*

Figure S11. *DEPT of compound **2** 9*

Figure S12. *^{1}H-^{1}H COSY of compound **2** 9*

Figure S13. *HSQC of compound **2** 10*

Figure S14. *HMBC of compound **2** 10*

Figure S15. *NOSEY of compound **2** 11*

Figure S16. *HR-ESI-MS of compound **2** 11*

Figure S17. *^{1}H-NMR of compound **3** 12*

Figure S18. *^{13}C-NMR of compound **3** 12*

Figure S19. *DEPT of compound **3** 13*

Figure S20. *^{1}H-^{1}H COSY of compound **3** 13*

Figure S21. *HSQC of compound **3** 14*

Figure S22. *HMBC of compound* ***3*** *14*

Figure S23. *NOSEY of compound* ***3*** *15*

Figure S24. *HR-ESI-MS of compound* ***3*** *15*

Figure S25. *^{1}H-NMR of compound* ***4*** *16*

Figure S26. *^{13}C-NMR of compound* ***4*** *16*

Figure S27. *DEPT of compound* ***4*** *17*

Figure S28. *^{1}H-^{1}H COSY of compound* ***4*** *17*

Figure S29. *HSQC of compound* ***4*** *18*

Figure S30. *HMBC of compound* ***4*** *18*

Figure S31. *NOSEY of compound* ***4*** *19*

Figure S32. *HR-ESI-MS of compound* ***4*** *19*

Figure S33. *^{1}H-NMR of compound* ***5*** *20*

Figure S34. *^{13}C-NMR of compound* ***5*** *20*

Figure S35. *DEPT of compound* ***5*** *21*

Figure S36. *^{1}H-^{1}H COSY of compound* ***5*** *21*

Figure S37. *HSQC of compound* ***5*** *22*

Figure S38. *HMBC of compound* ***5*** *22*

Figure S39. *NOSEY of compound* ***5*** *23*

Figure S40. *HR-ESI-MS of compound* ***5*** *23*

Figure S41. *^{1}H-NMR of compound* ***6*** *24*

Figure S42. *^{13}C-NMR of compound* ***6*** *24*

Figure S43. *DEPT of compound* ***6*** *25*

Figure S44. *^{1}H-^{1}H COSY of compound* ***6*** *25*

Figure S45. *HSQC of compound* ***6****26*

Figure S46. *HMBC of compound* ***6*** *26*

Figure S47. *NOESY of compound* ***6*** *27*

Figure S48. *HR-ESI-MS of compound* ***6*** *27*

Table S1. *Gibbs free energies[a] and equilibrium populations[b] of low-energy conformers of dalesconoside A (**1**) 27*

Table S2. *Cartesian coordinates for the low-energy reoptimized MMFF conformers of dalesconoside A (**1**) at B3LYP/6-31G(d,p) level of theory in gas 27*

Table S3. *Gibbs free energies[a] and equilibrium populations[b] of low-energy conformers of dalesconoside B (**2**) 33*

Table S4. *Cartesian coordinates for the low-energy reoptimized MMFF conformers of dalesconoside B (**2**) at B3LYP/6-31G(d,p) level of theory in gas 33*

Table S5. *Gibbs free energies[a] and equilibrium populations[b] of low-energy conformers of dalesconoside C (**3**) 28*

Table S6. *Cartesian coordinates for the low-energy reoptimized MMFF conformers of dalesconoside C (**3**) at B3LYP/6-31G(d,p) level of theory in gas 39*

Table S7. *Gibbs free energies[a] and equilibrium populations[b] of low-energy conformers of dalesconoside D (**6**) 42*

Table S8. *Cartesian coordinates for the low-energy reoptimized MMFF conformers of dalesconoside D (**6**) at B3LYP/6-31G(d,p) level of theory in gas* 43

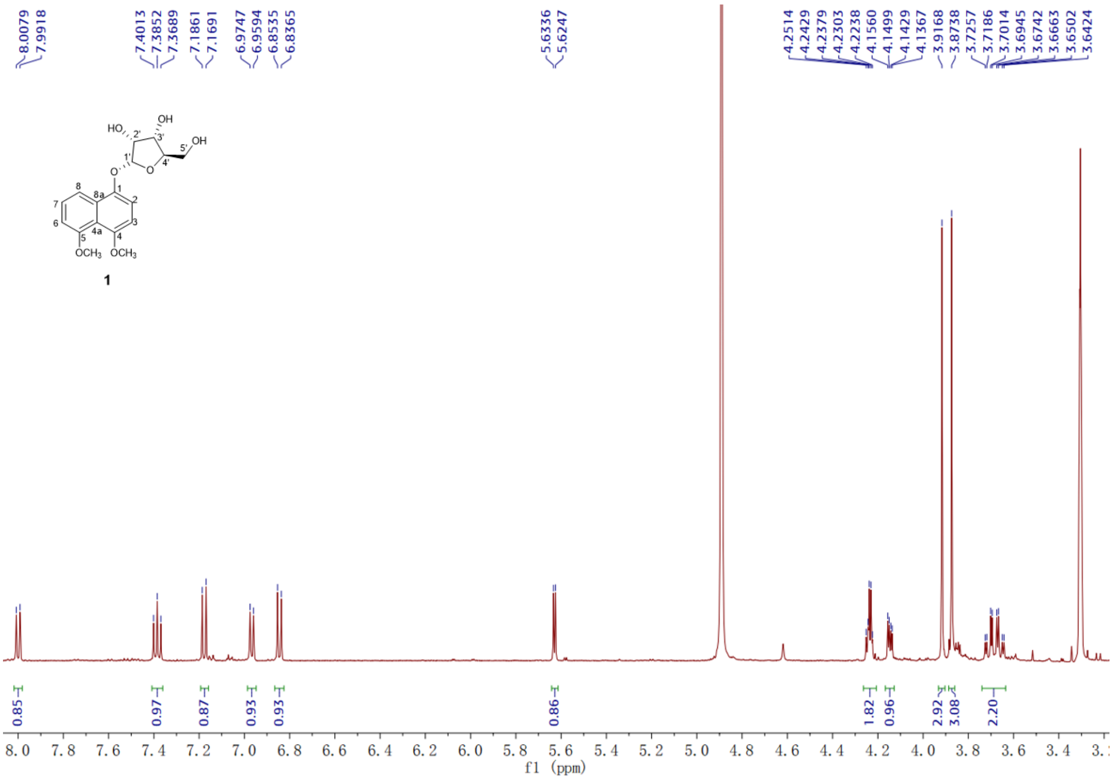

Figure S1. *^{1}H-NMR of compound **1***

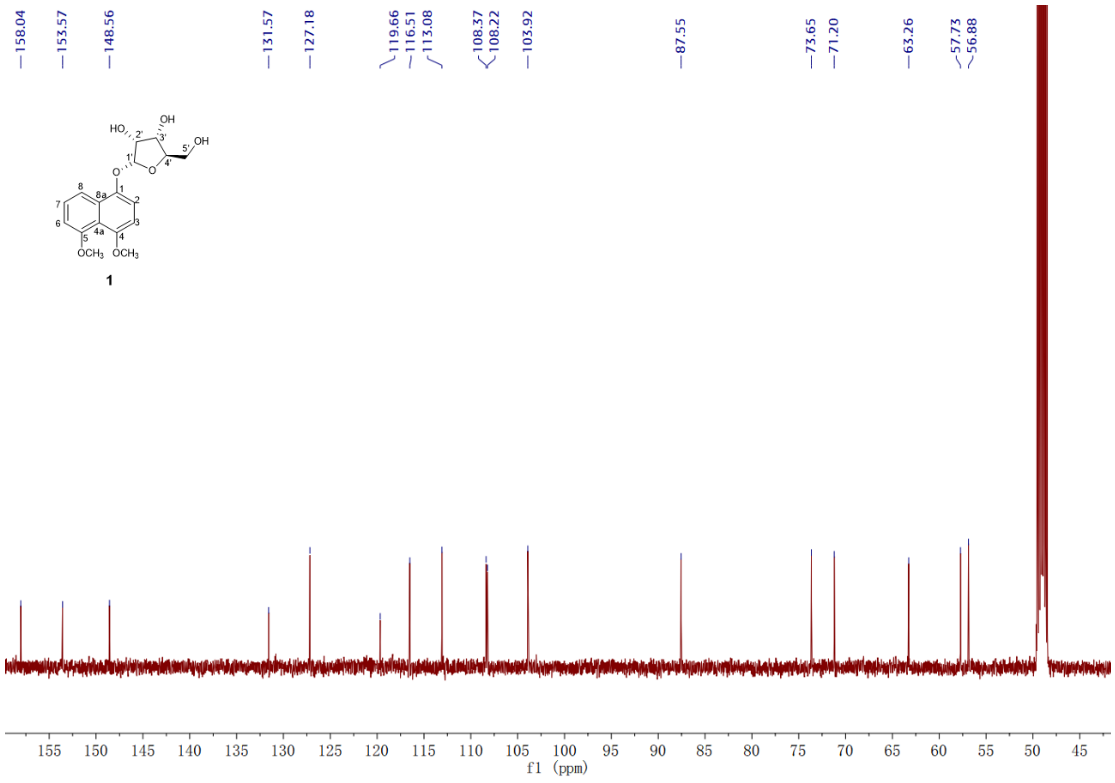

Figure S2. *^{13}C-NMR of compound* ***1***

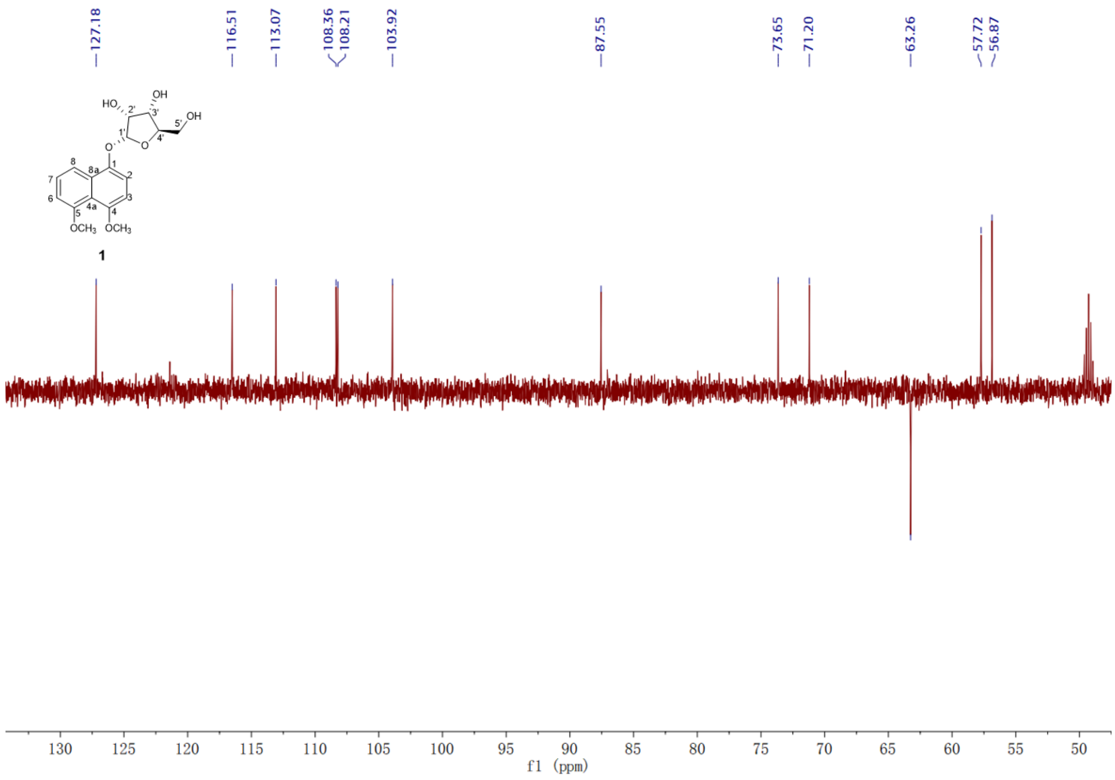

Figure S3. *DEPT of compound* ***1***

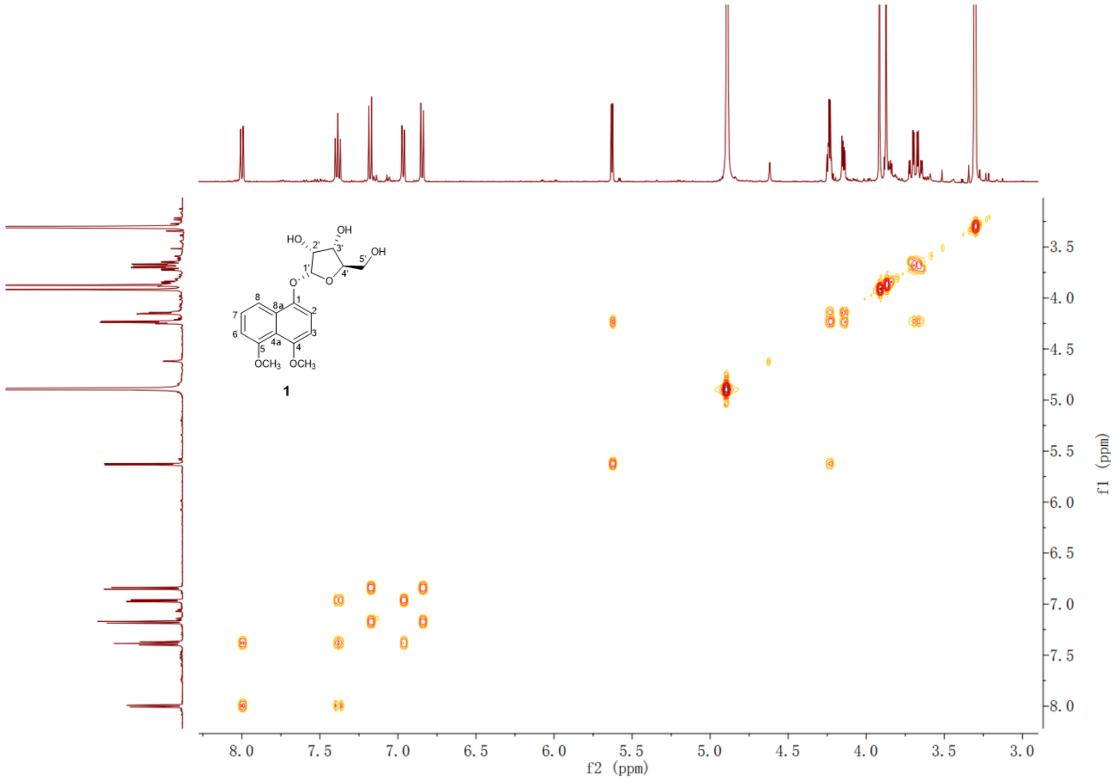

Figure S4. ^1H-^1H *COSY of compound* ***1***

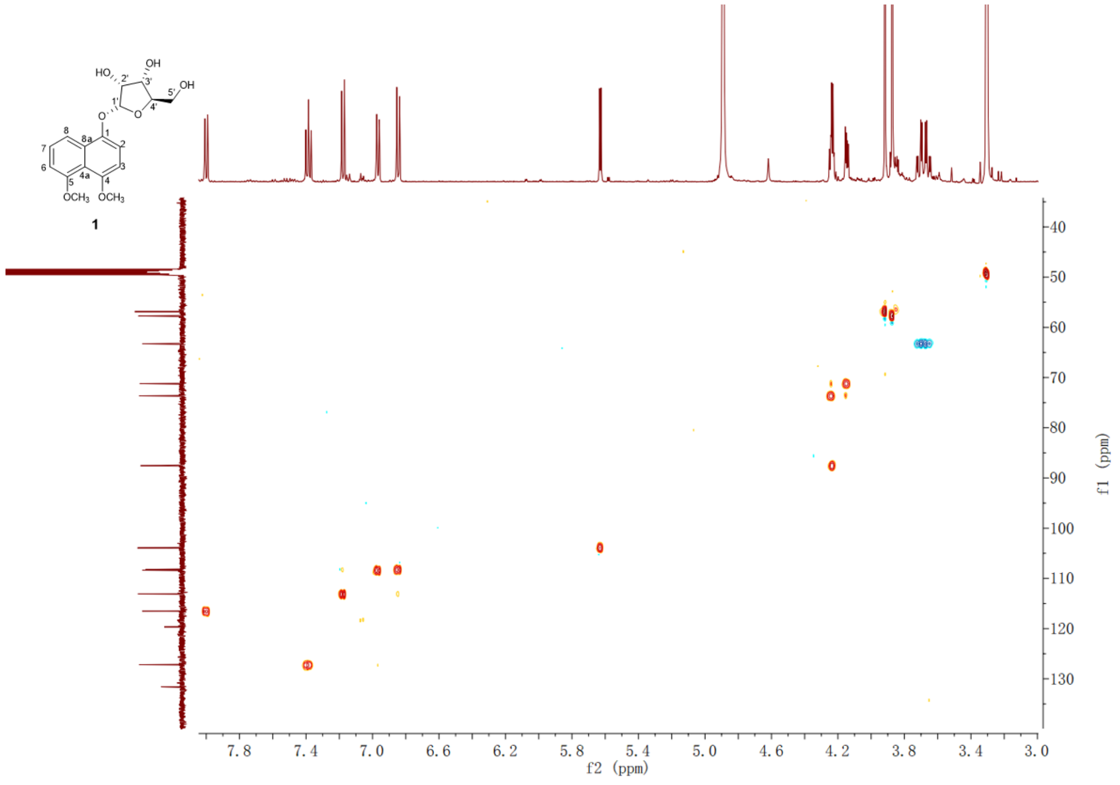

Figure S5. *HSQC of compound* ***1***

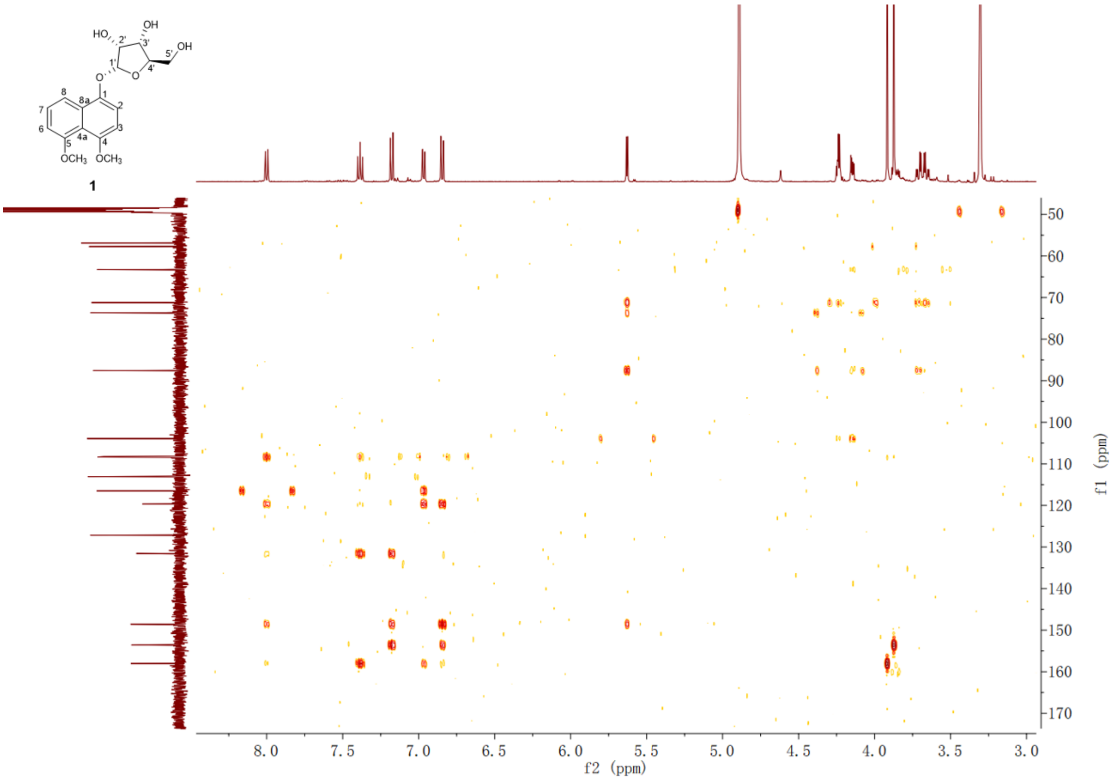

Figure S6. *HMBC of compound **1***

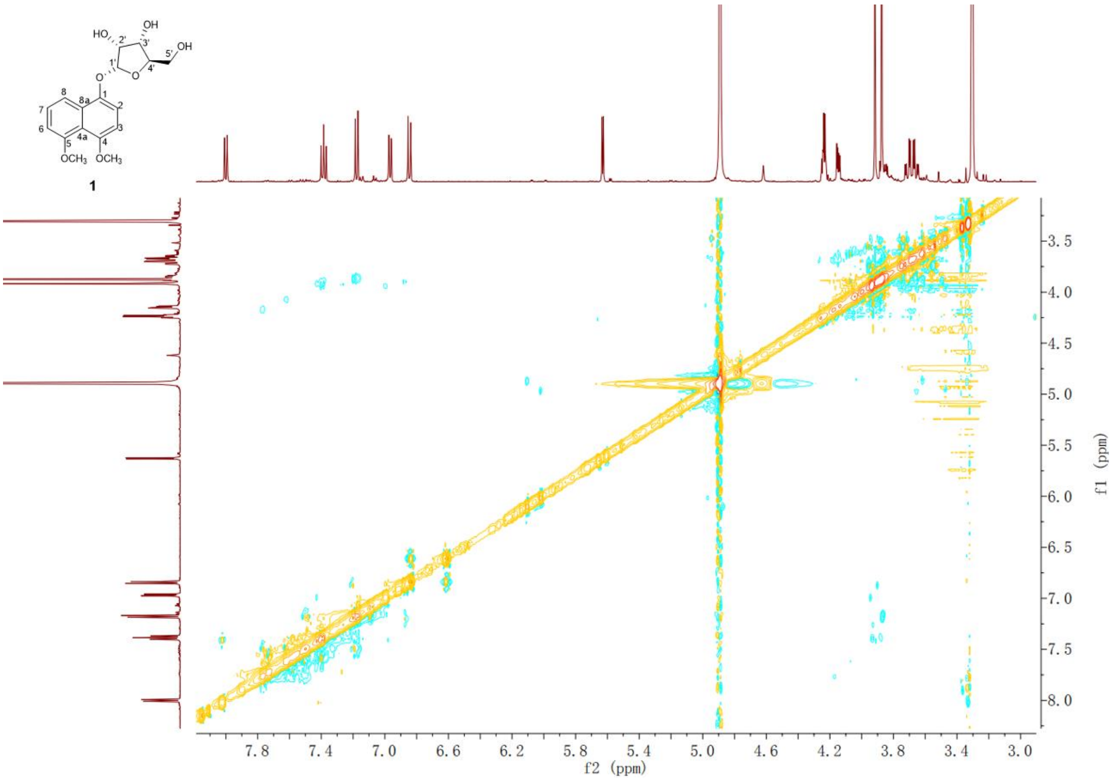

Figure S7. *NOSEY of compound **1***

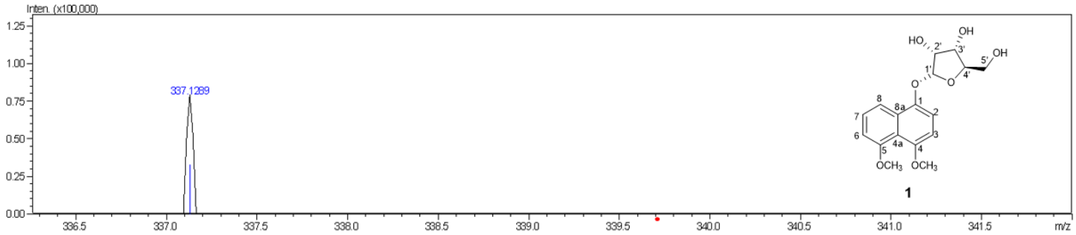

Figure S8. *HR-ESI-MS of compound **1***

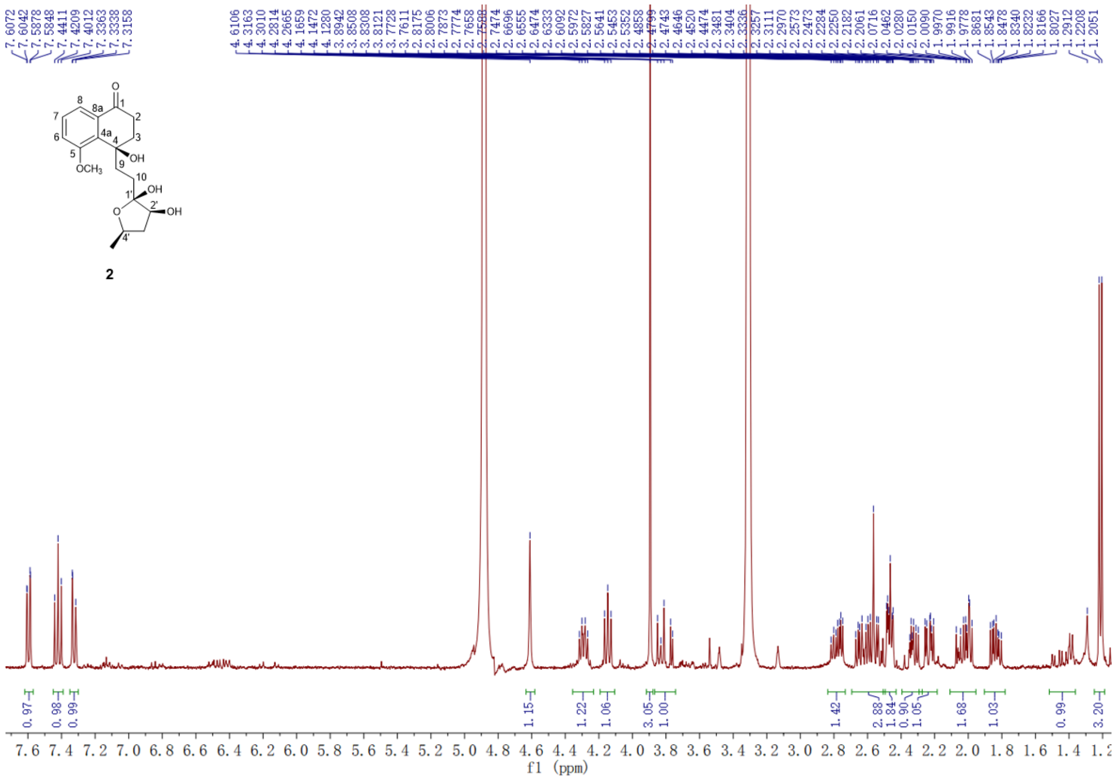

Figure S9. *^{1}H-NMR of compound **2***

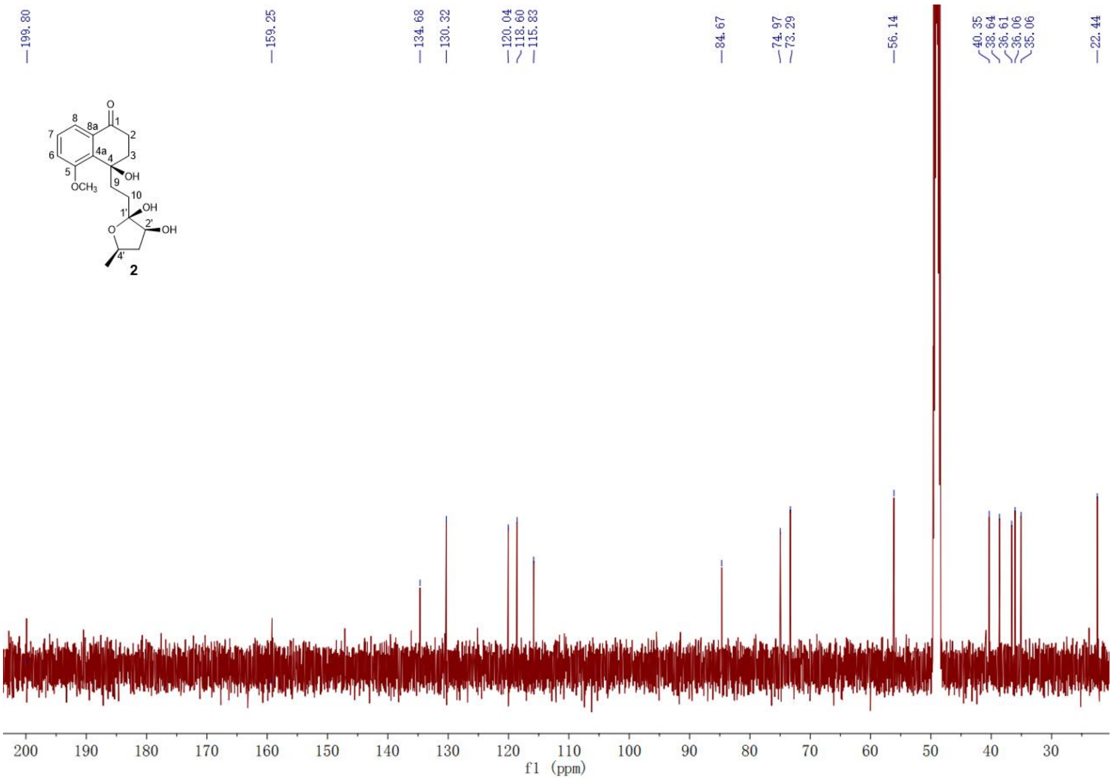

Figure S10. *^{13}C-NMR of compound **2***

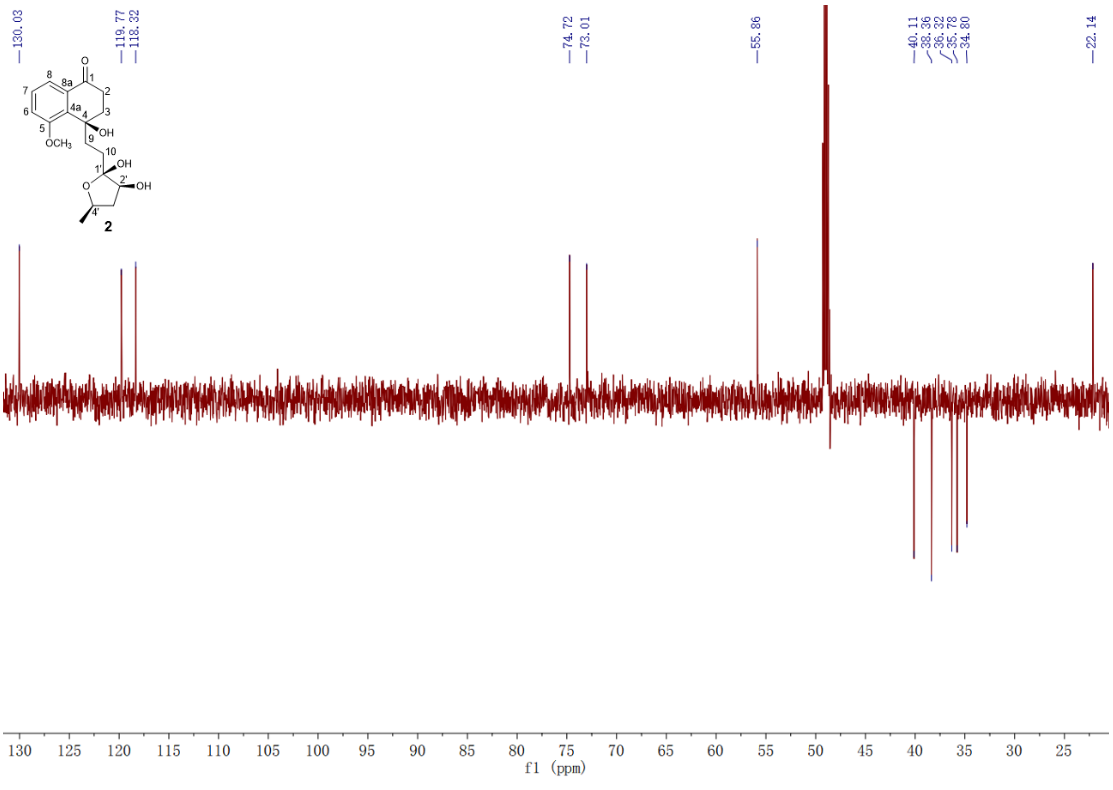

Figure S11. *DEPT of compound **2***

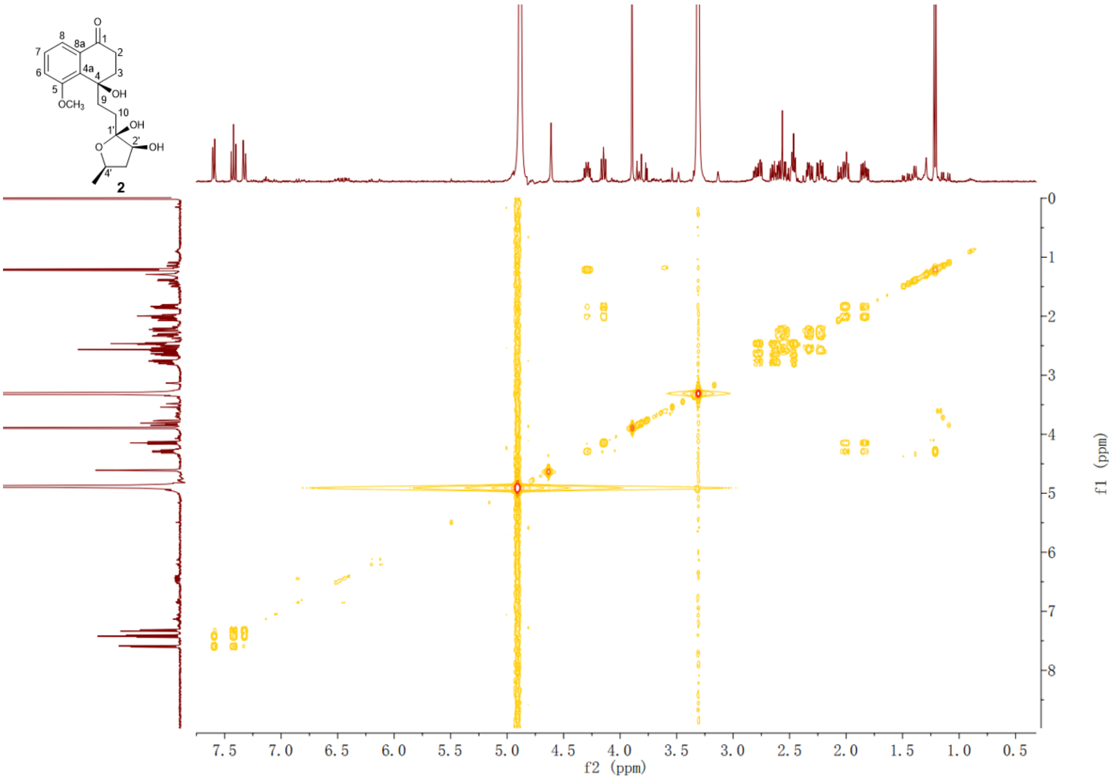

Figure S12. *¹H-¹H COSY of compound* ***2***

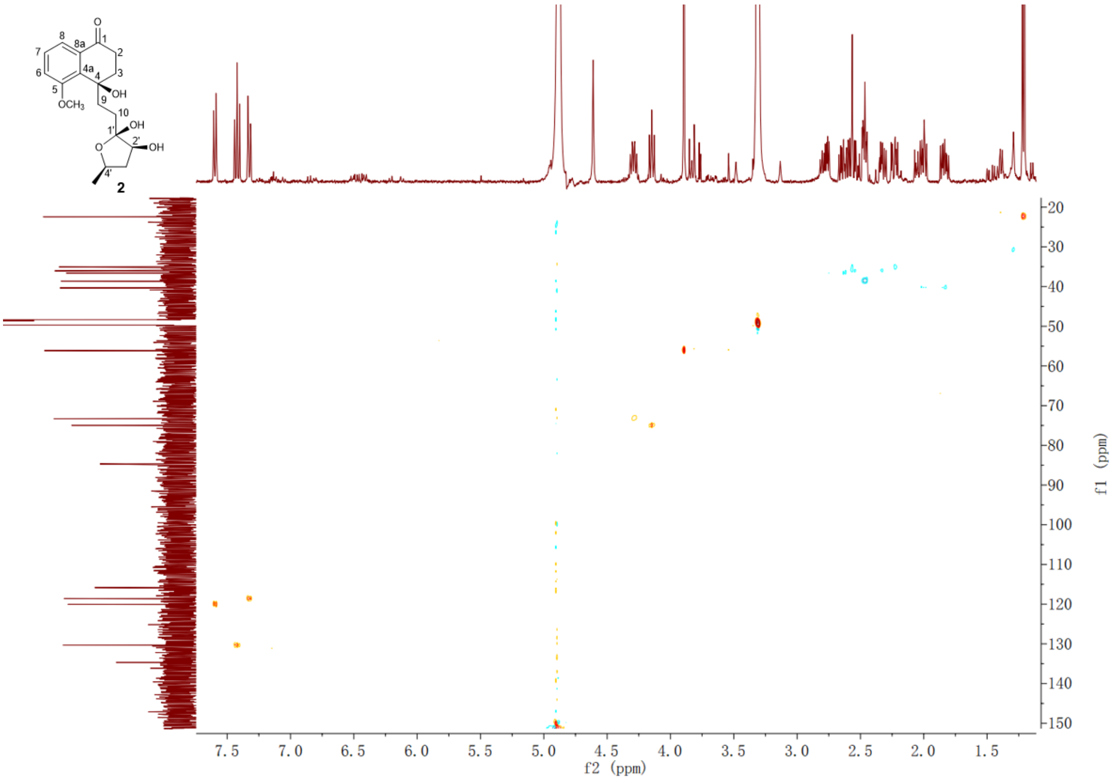

Figure S13. *HSQC of compound **2***

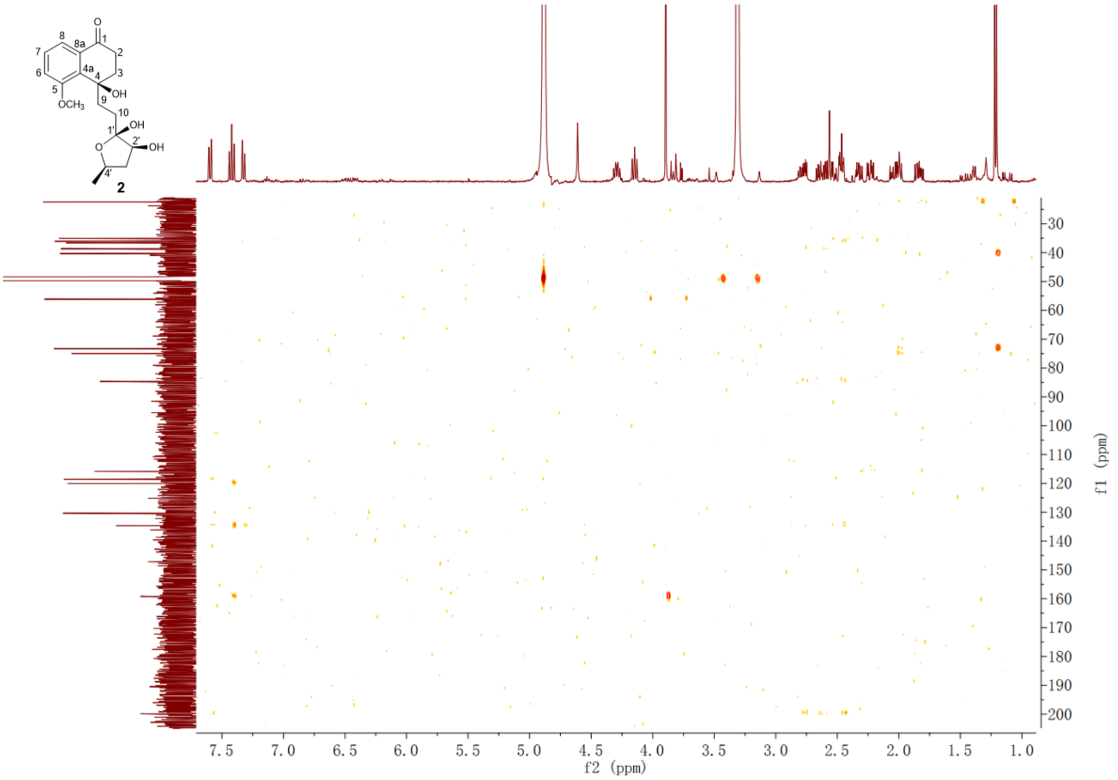

Figure S14. *HMBC of compound **2***

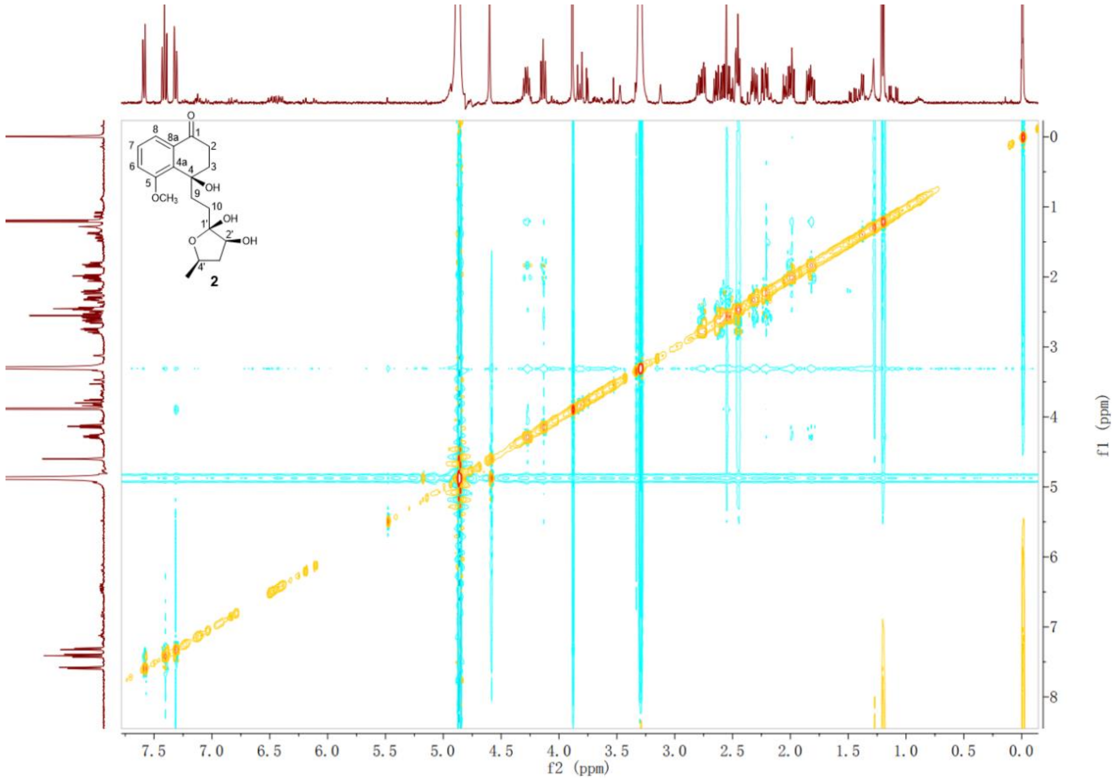

Figure S15. *NOSEY of compound* ***2***

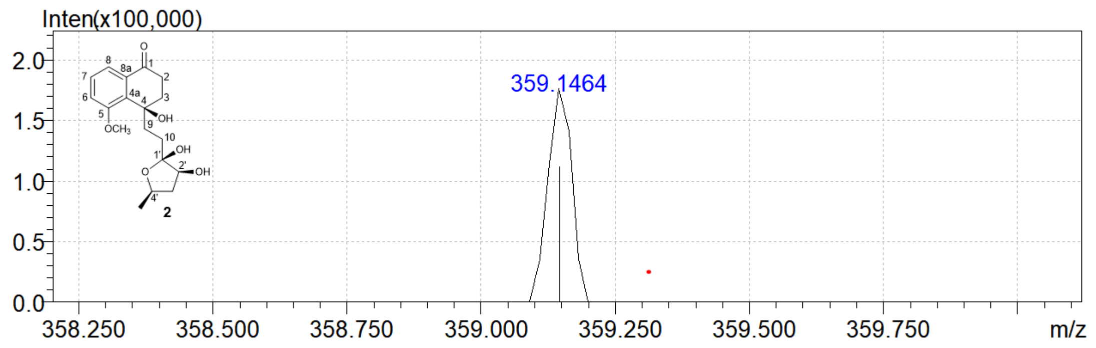

Figure S16. *HR-ESI-MS of compound* ***2***

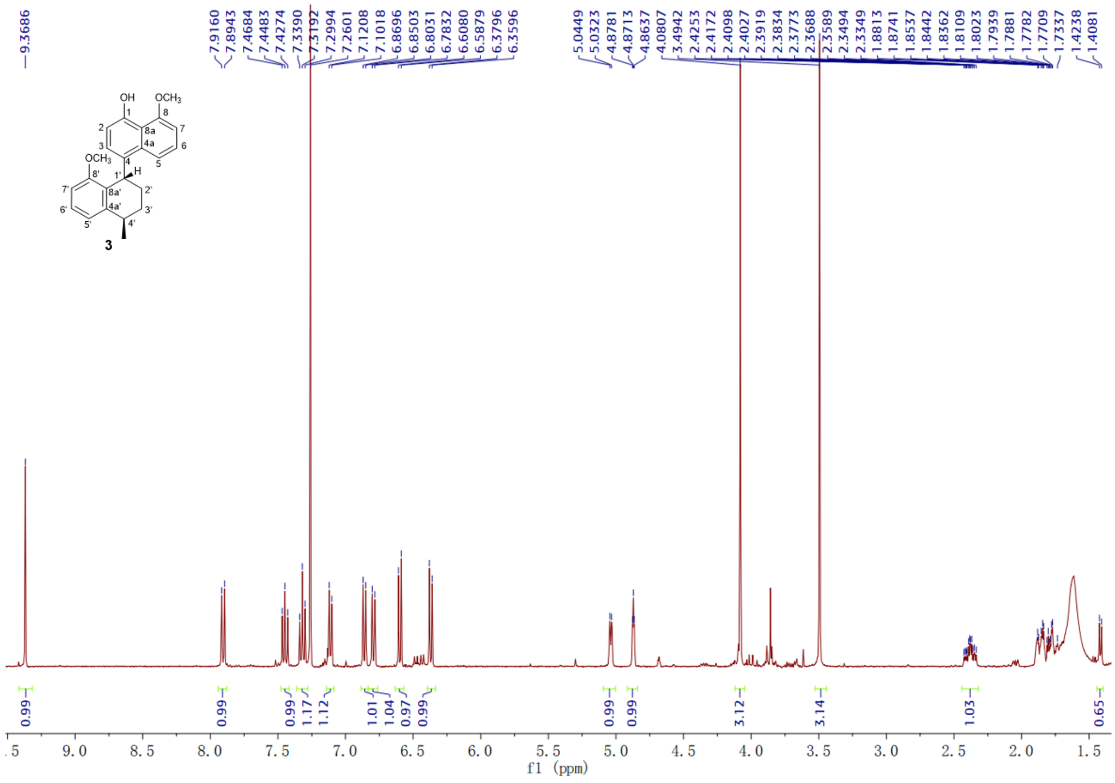

Figure S17. *^{1}H-NMR of compound* **3**

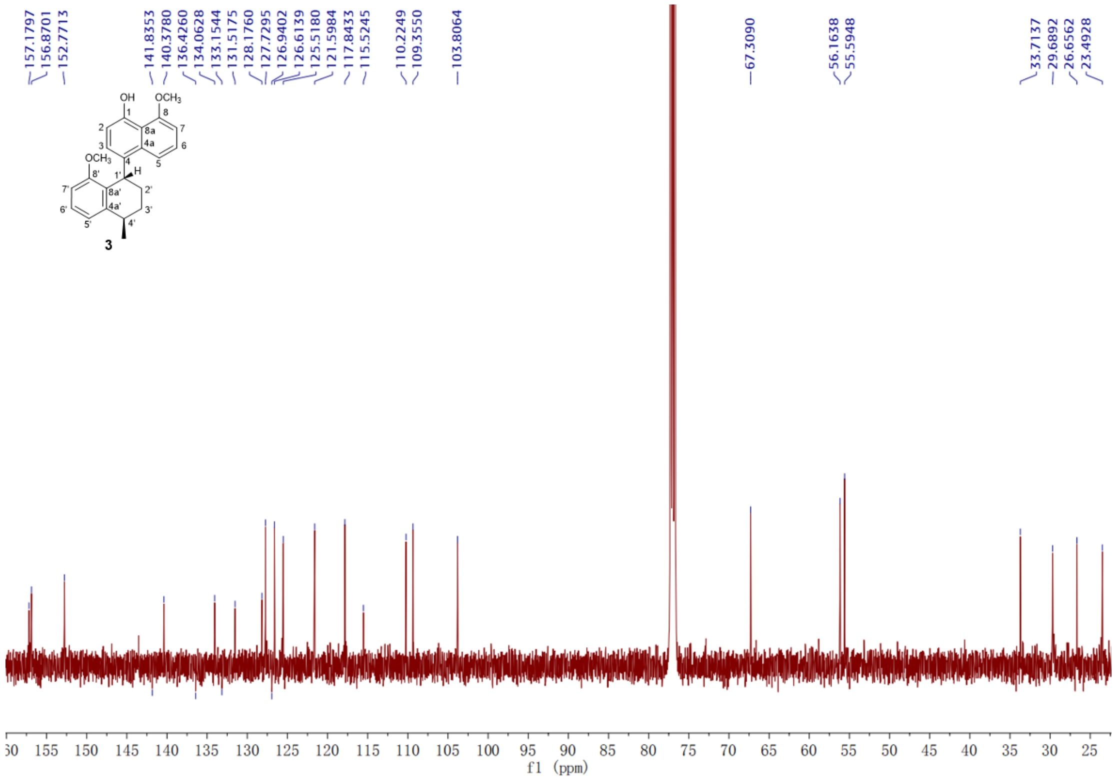

Figure S18. *^{13}C-NMR of compound* ***3***

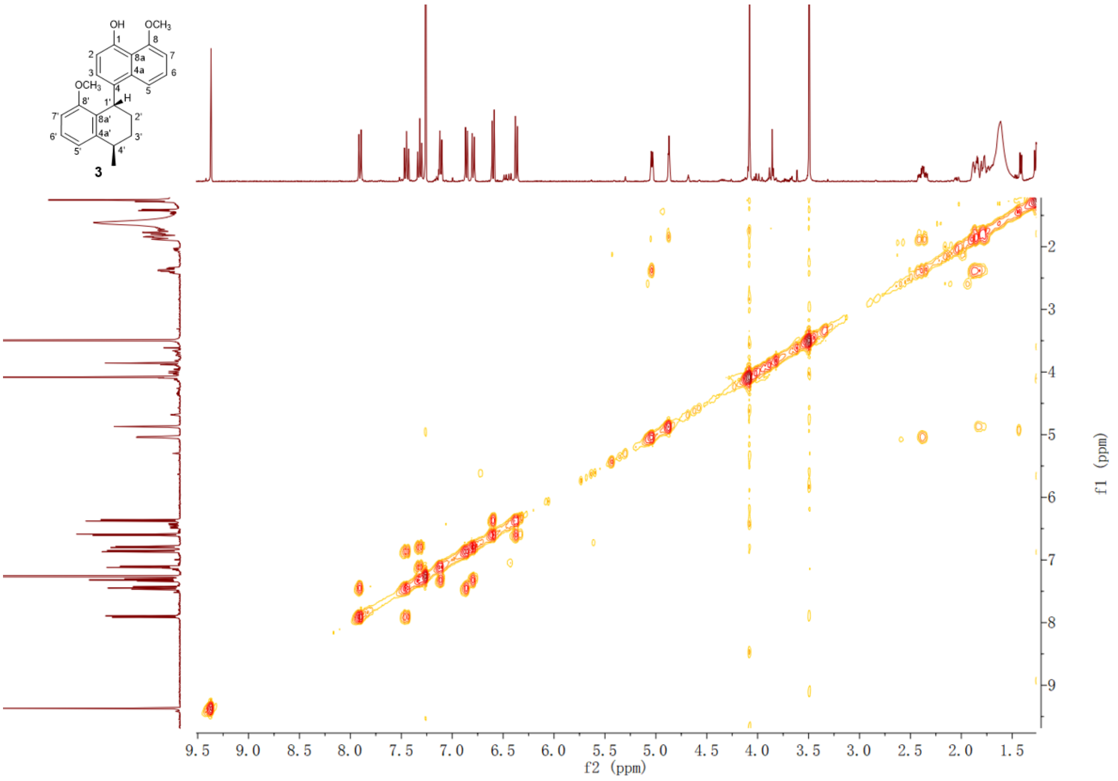

Figure S19. *DEPT of compound* ***3***

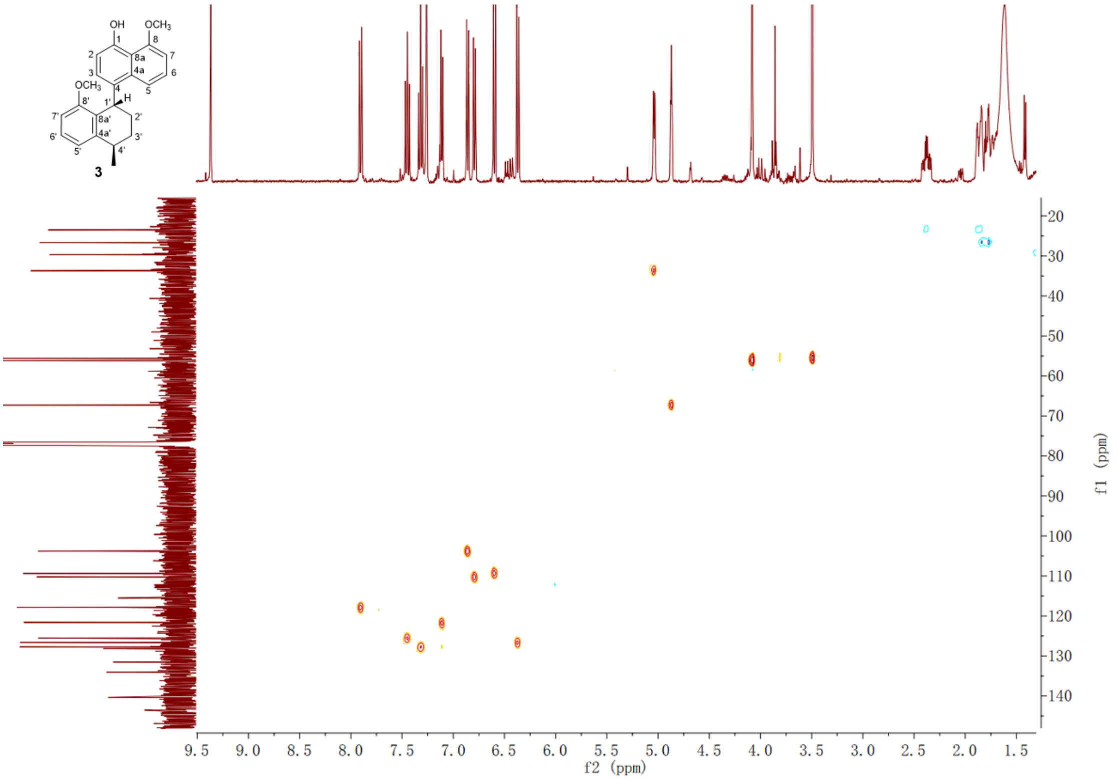

Figure S20. *^{1}H-^{1}H COSY of compound **3***

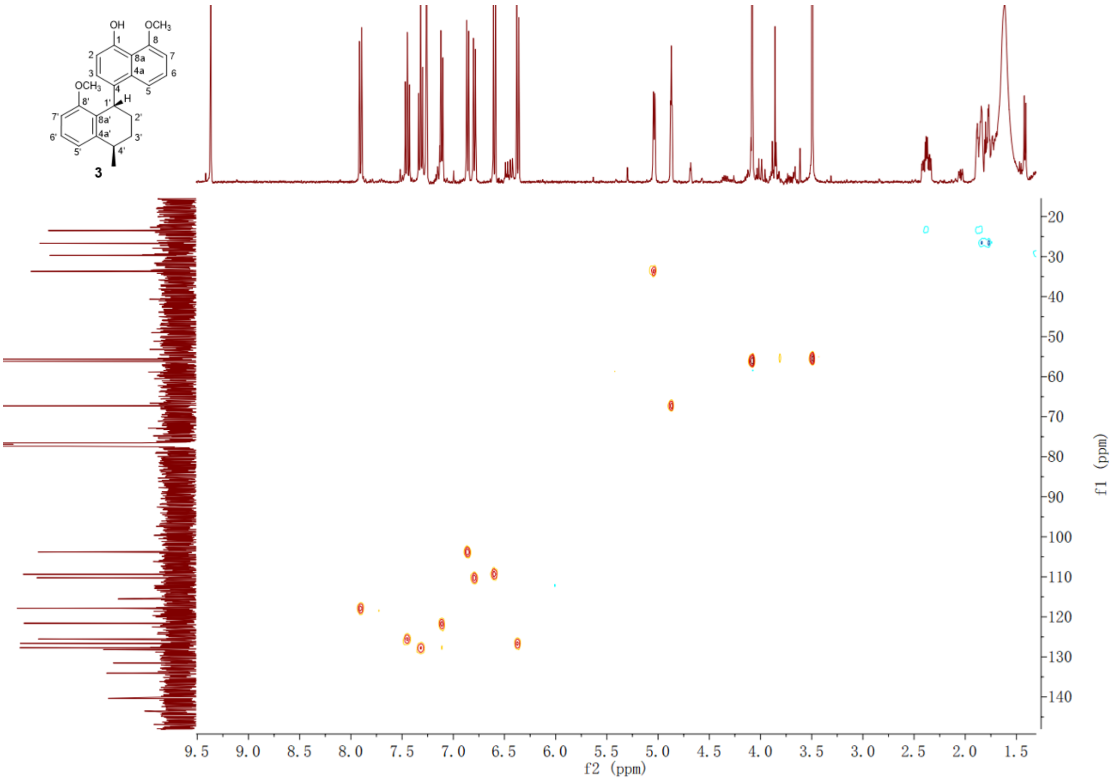

Figure S21. *HSQC of compound **3***

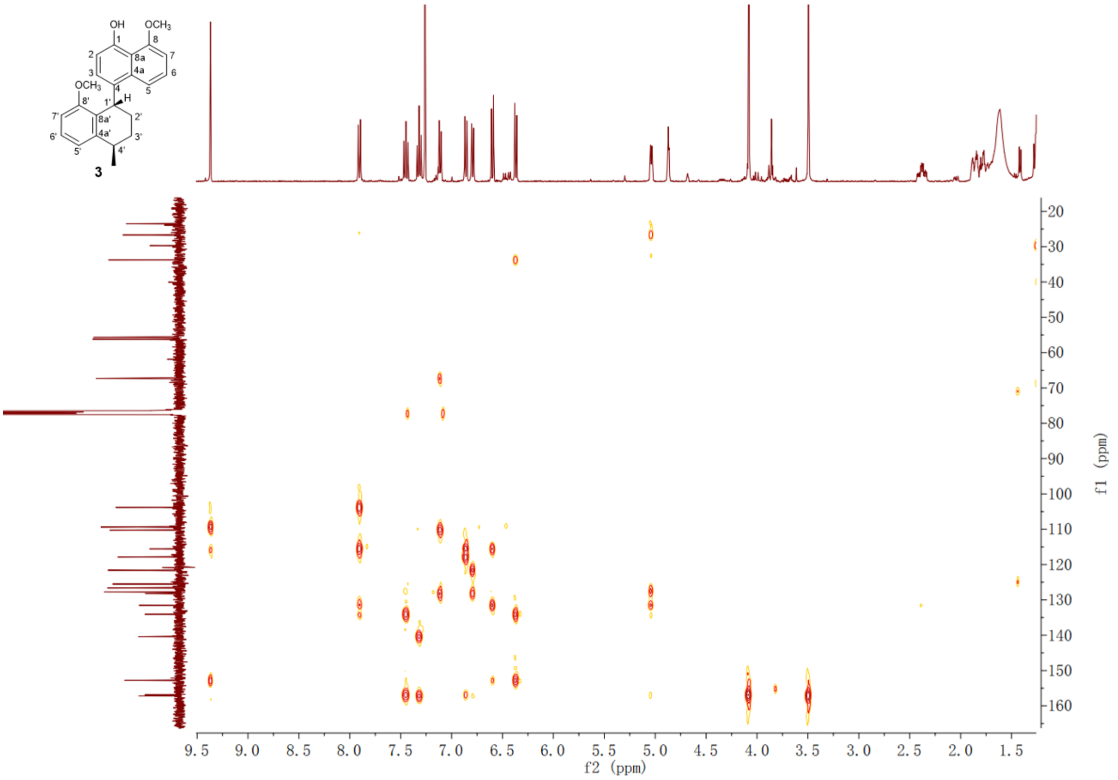

Figure S22. *HMBC of compound* ***3***

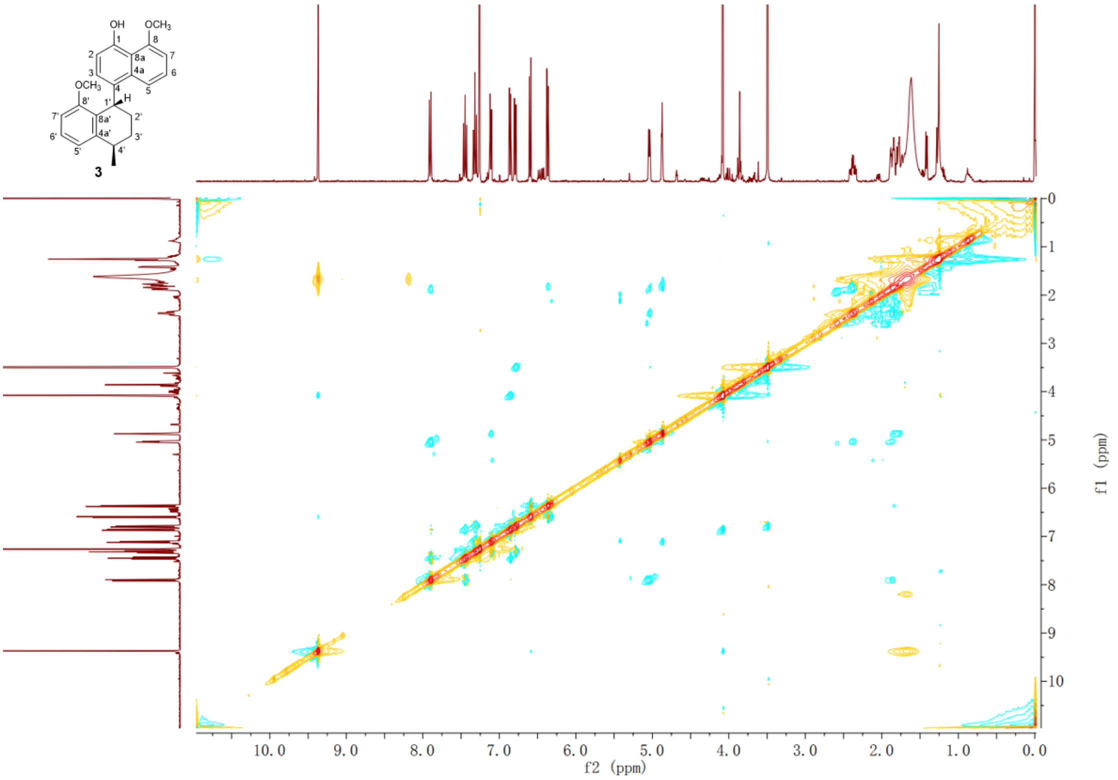

Figure S23. *NOSEY of compound* ***3***

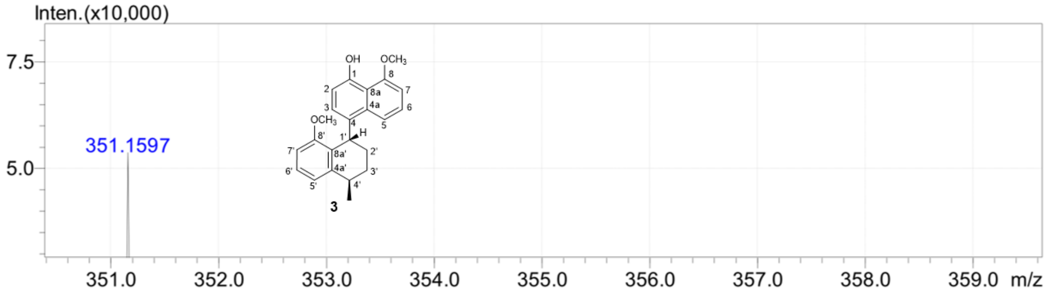

Figure S24. *HR-ESI-MS of compound* ***3***

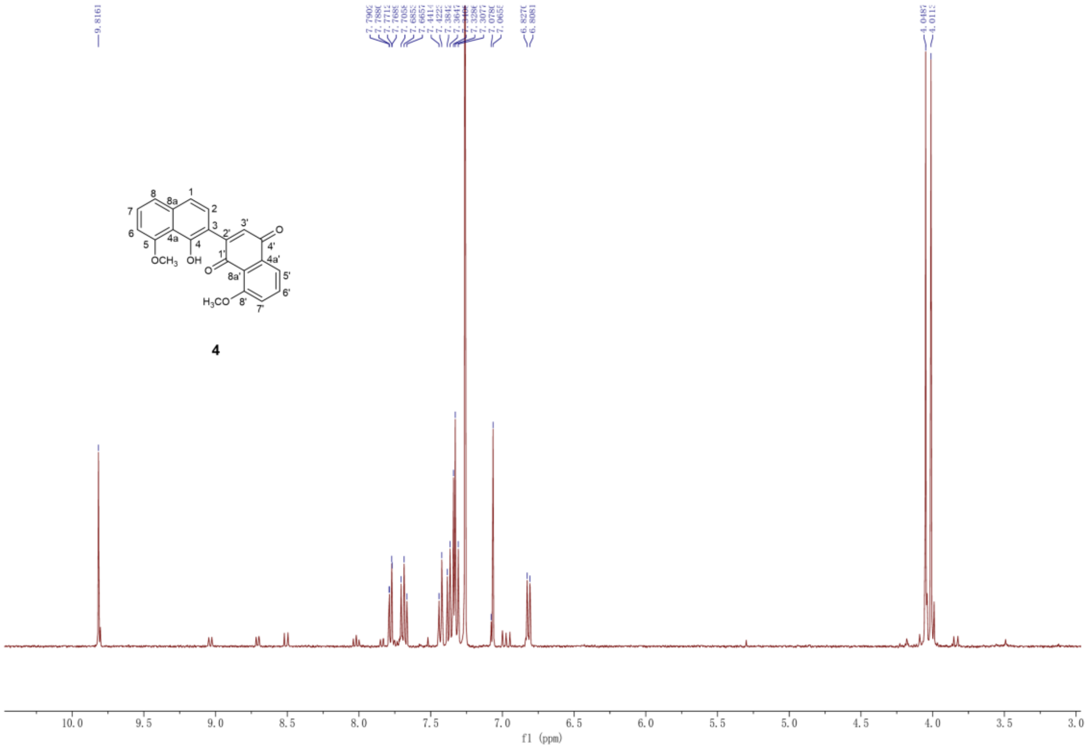

Figure S25. *^{1}H-NMR of compound* ***4***

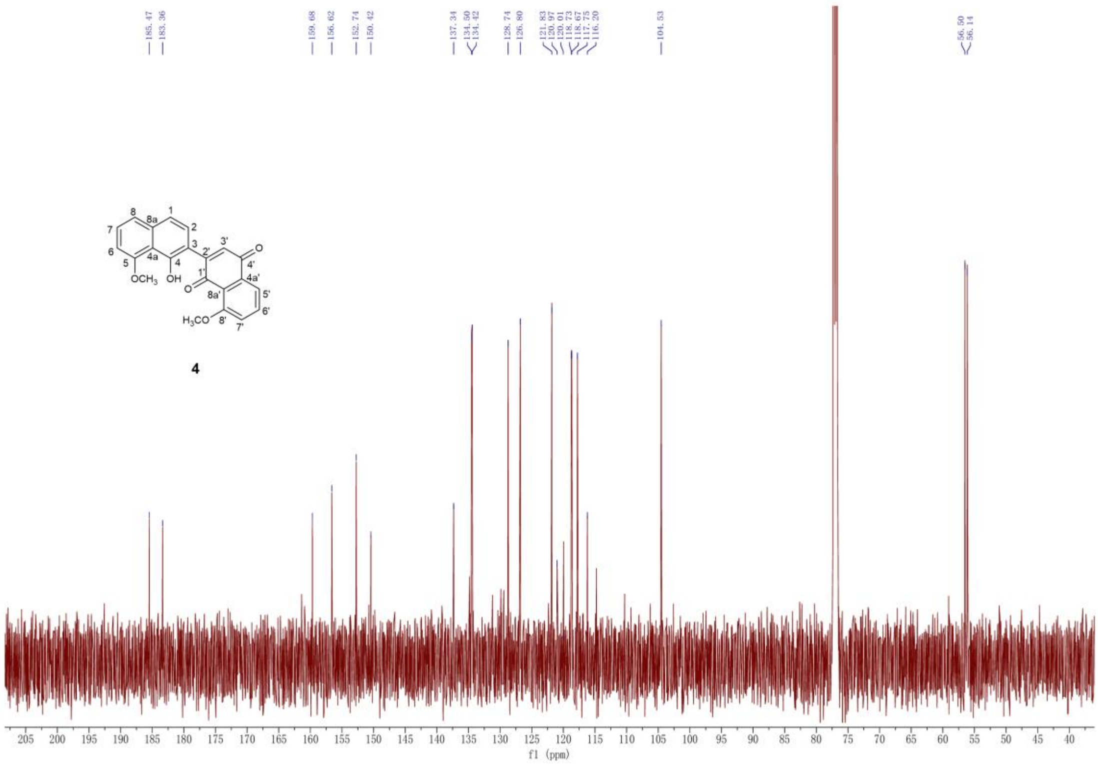

Figure S26. *^{13}C-NMR of compound* ***4***

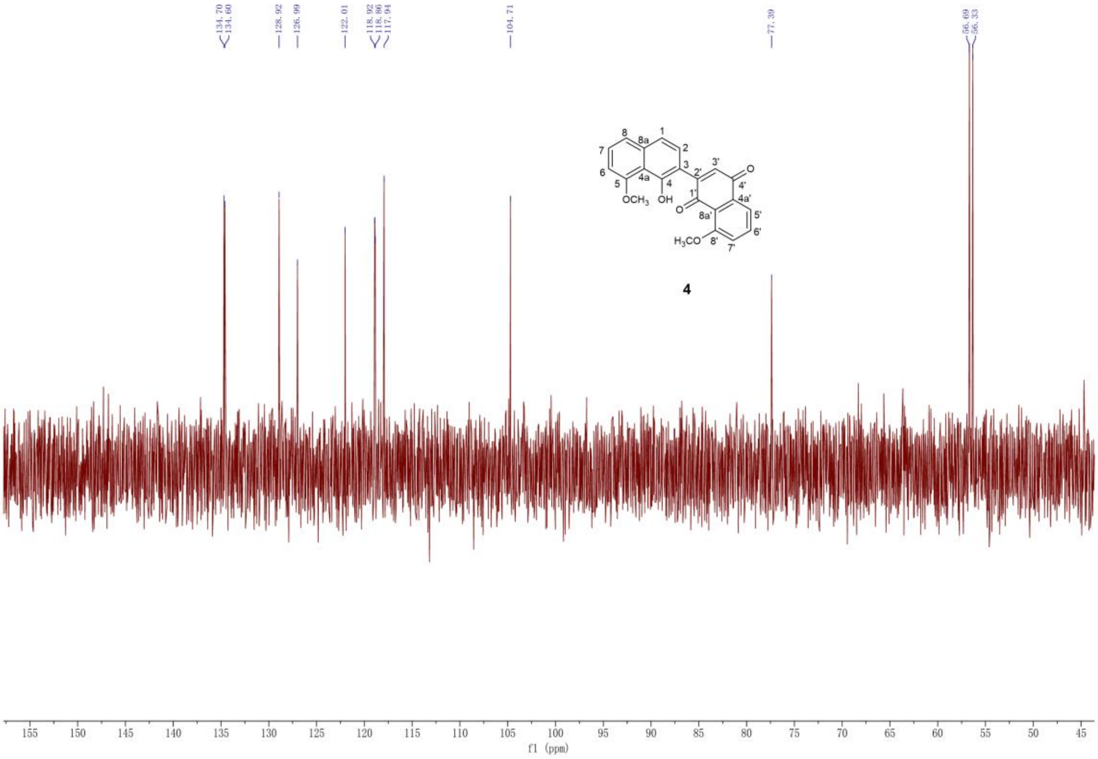

Figure S27. *DEPT of compound* ***4***

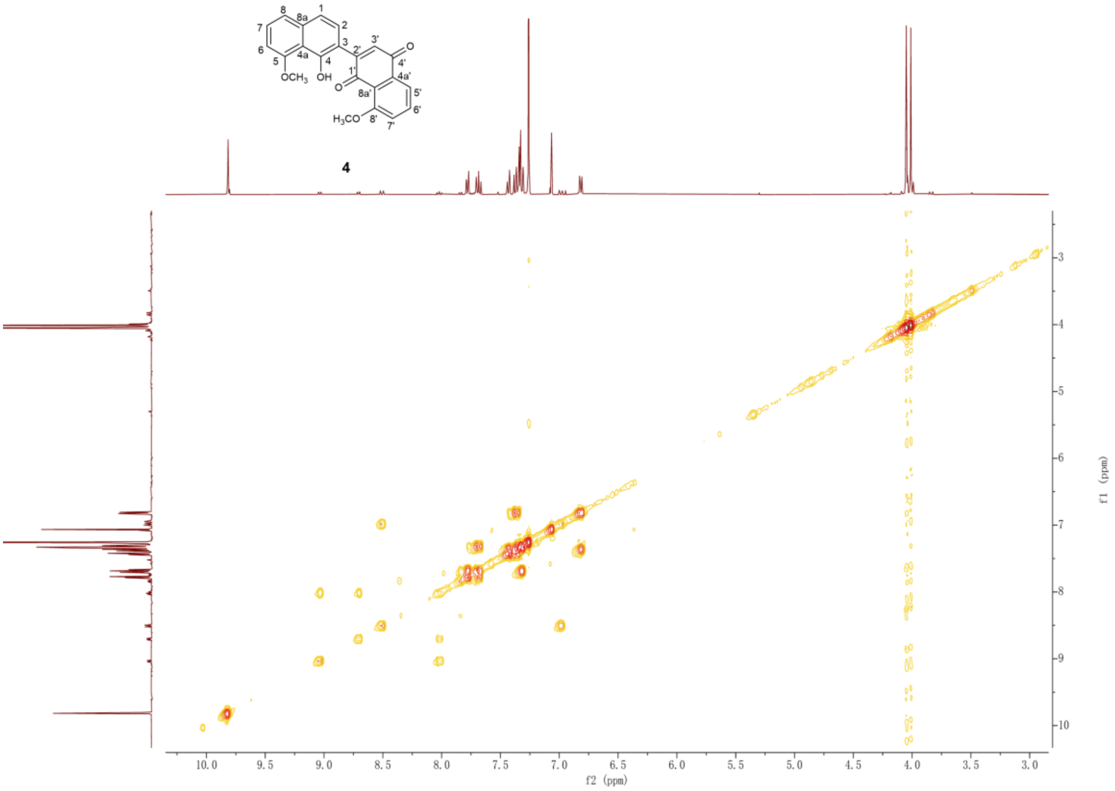

Figure S28. *^{1}H-^{1}H COSY of compound **4***

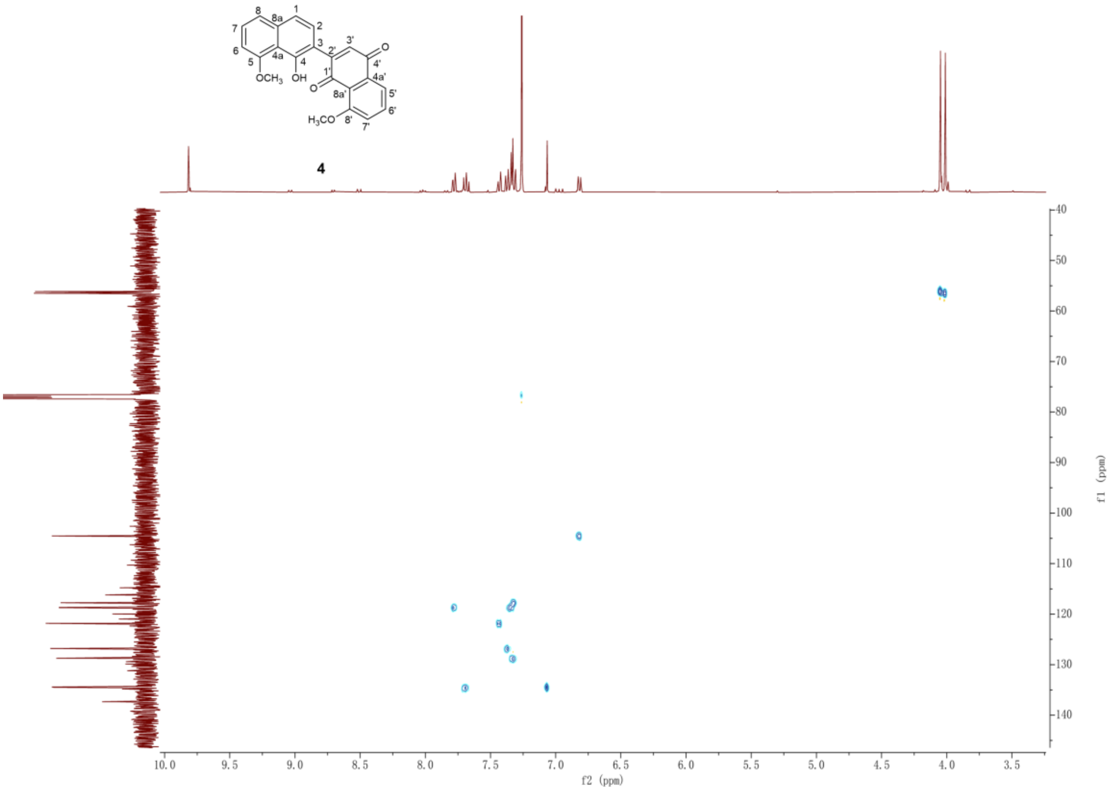

Figure S29. *HSQC of compound **4***

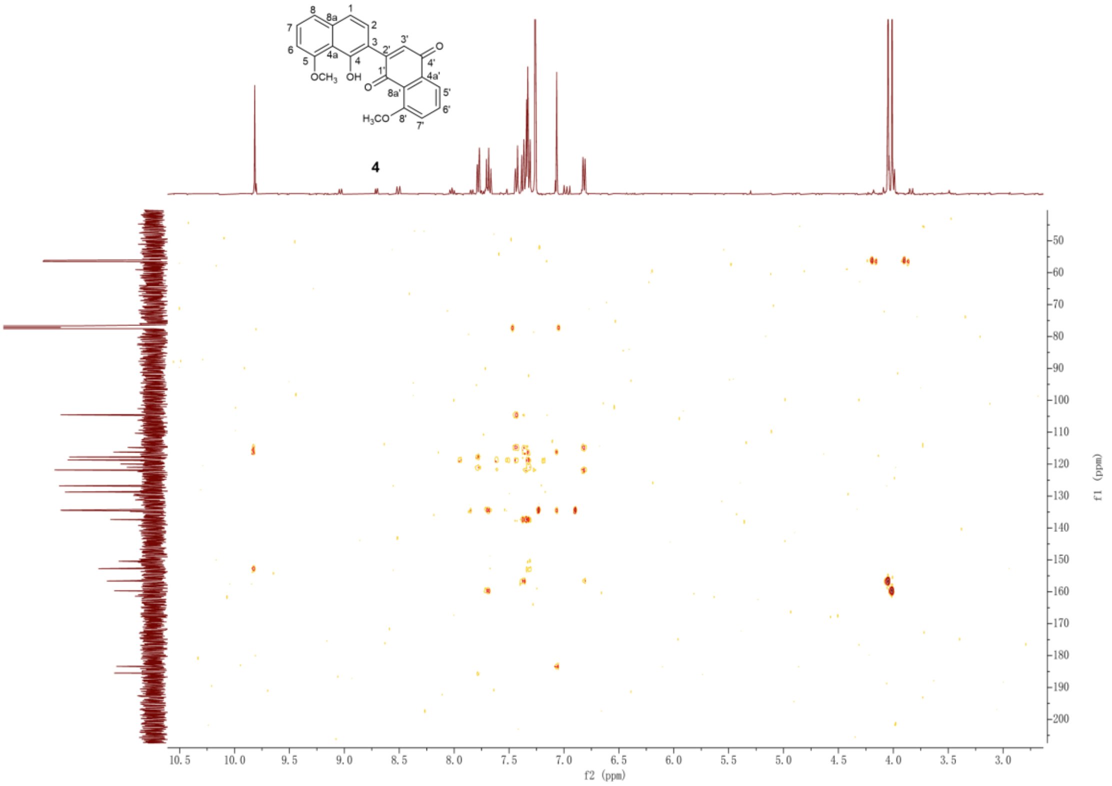

Figure S30. *HMBC of compound **4***

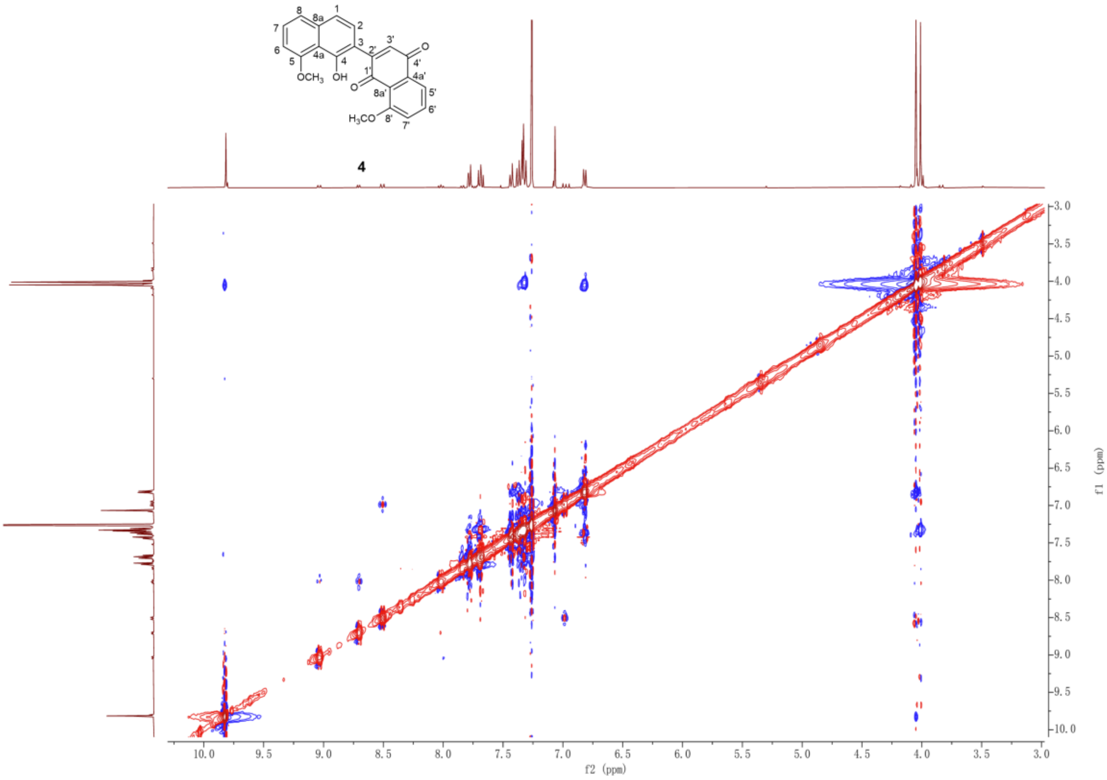

Figure S31. *NOSEY of compound* ***4***

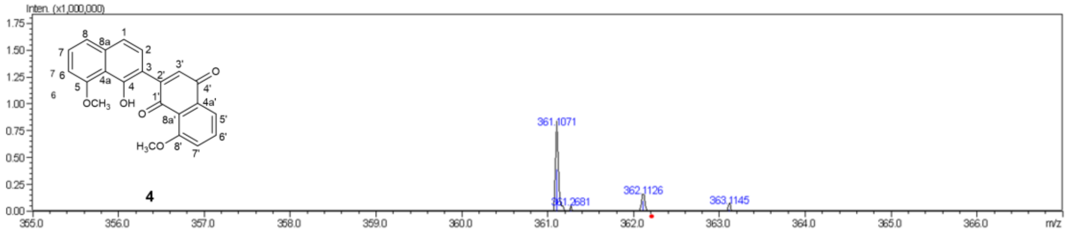

Figure S32. *HR-ESI-MS of compound* ***4***

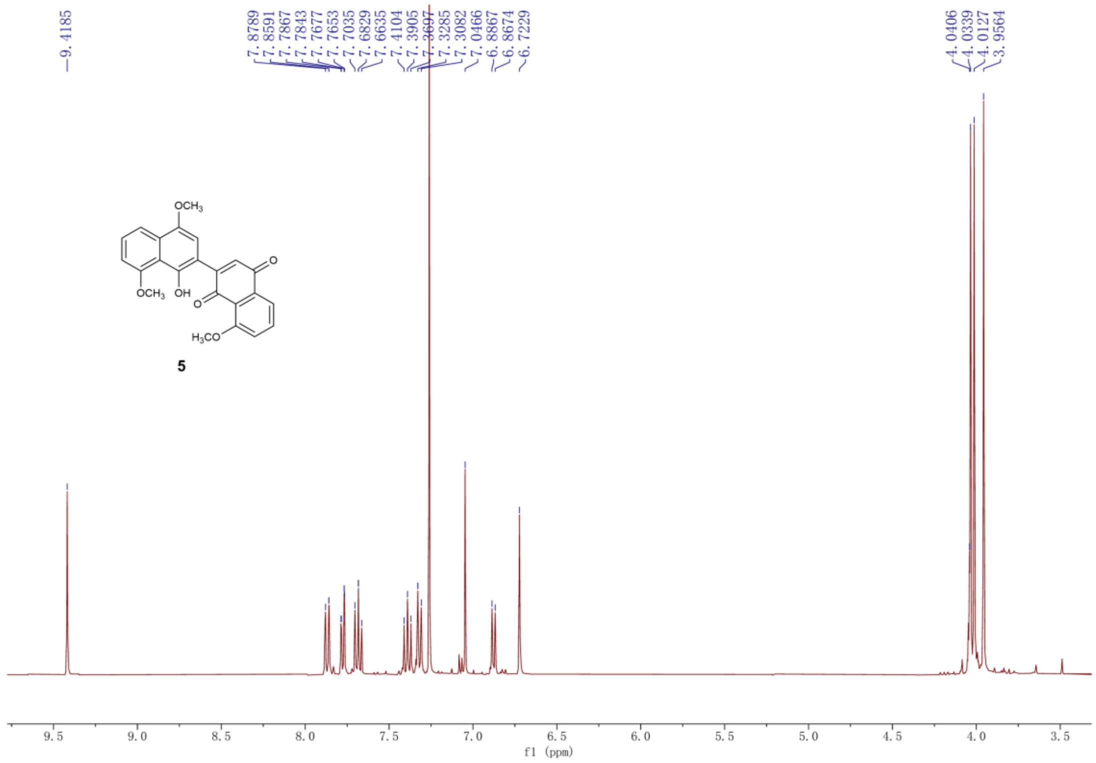

Figure S33. *^{1}H-NMR of compound* ***5***

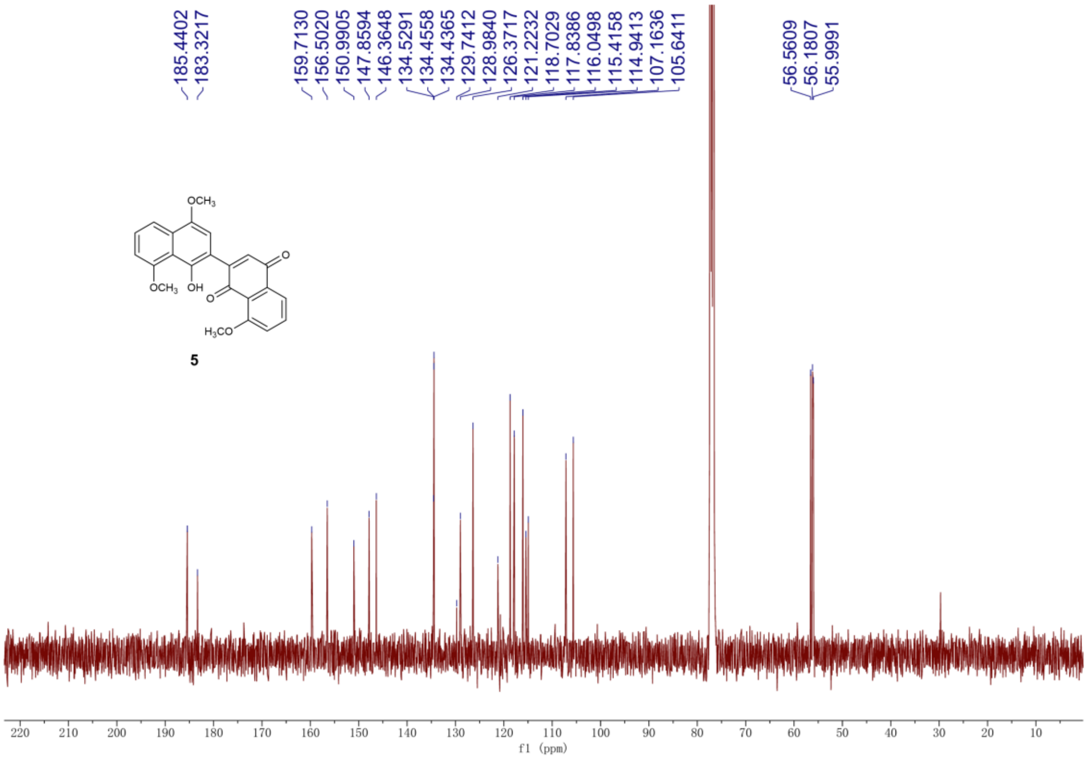

Figure S34. *^{13}C-NMR of compound **5***

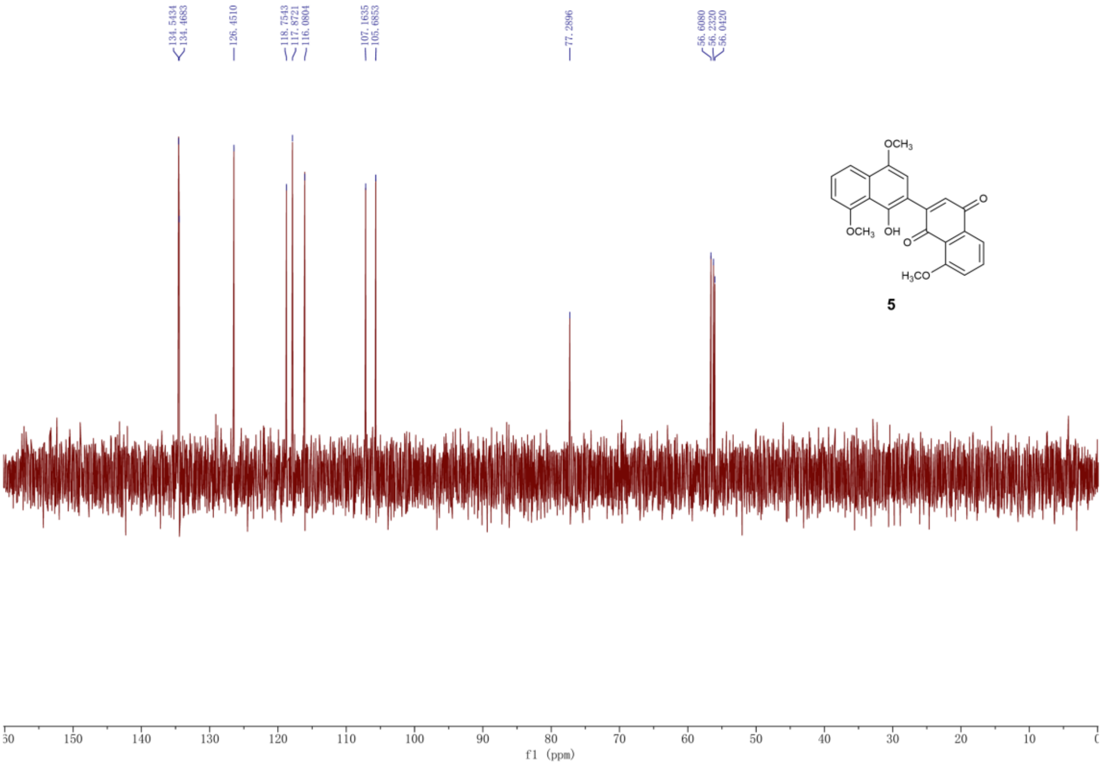

Figure S35. *DEPT of compound **5***

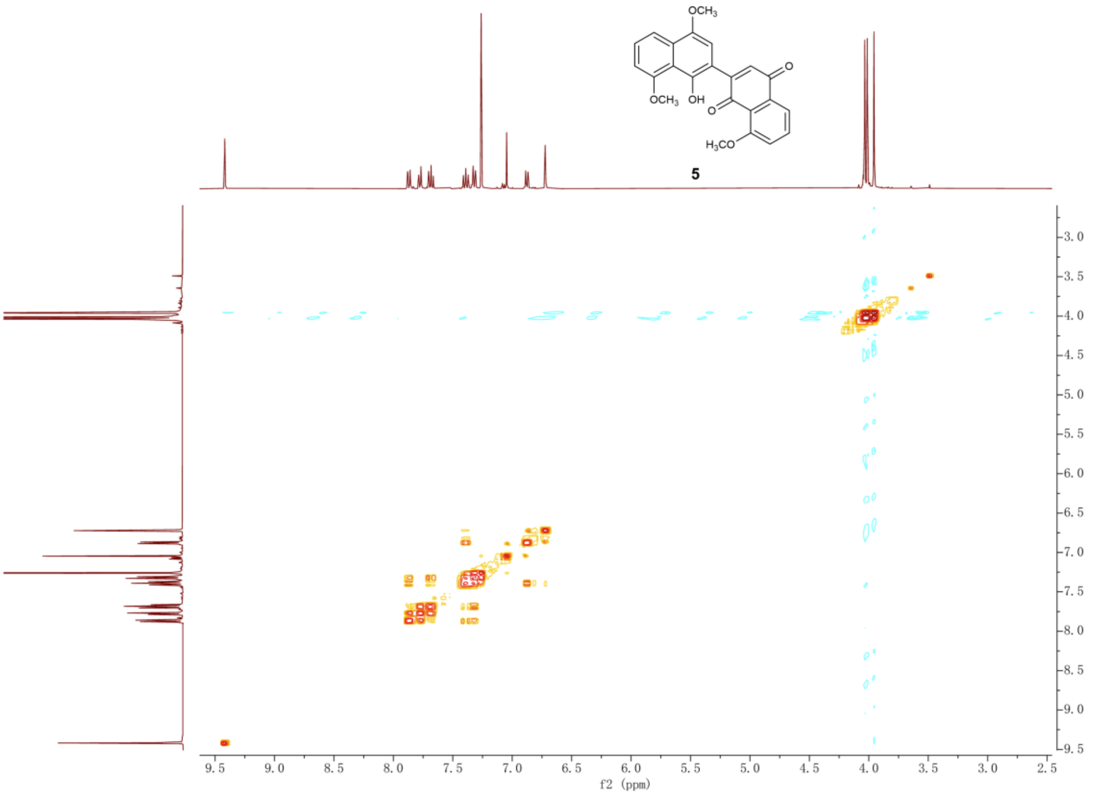

Figure S36. *^{1}H-^{1}H COSY of compound* ***5***

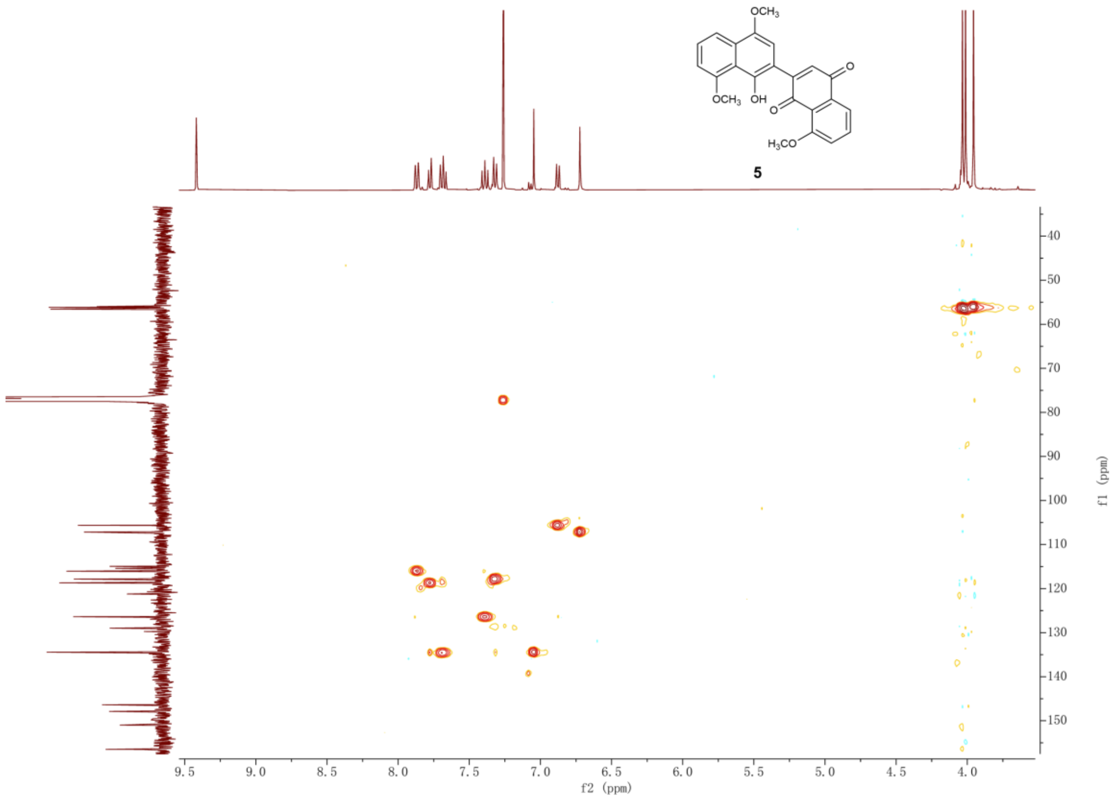

Figure S37. *HSQC of compound* ***5***

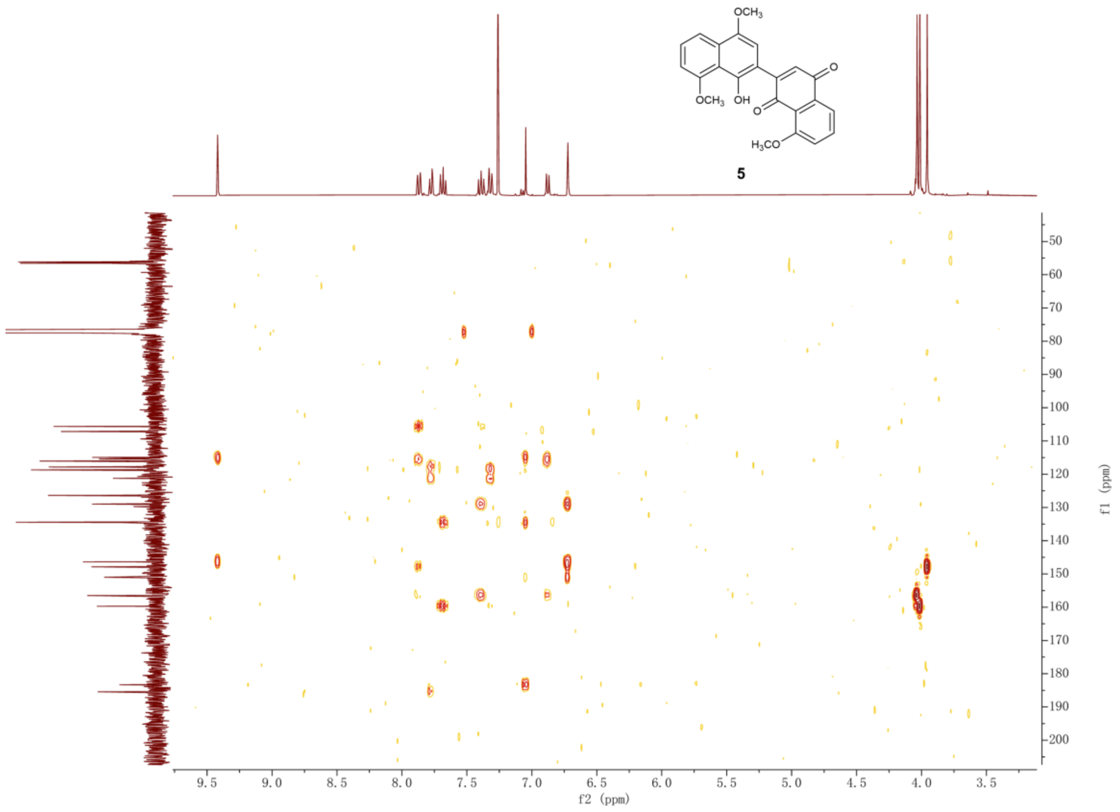

Figure S38. *HMBC of compound* ***5***

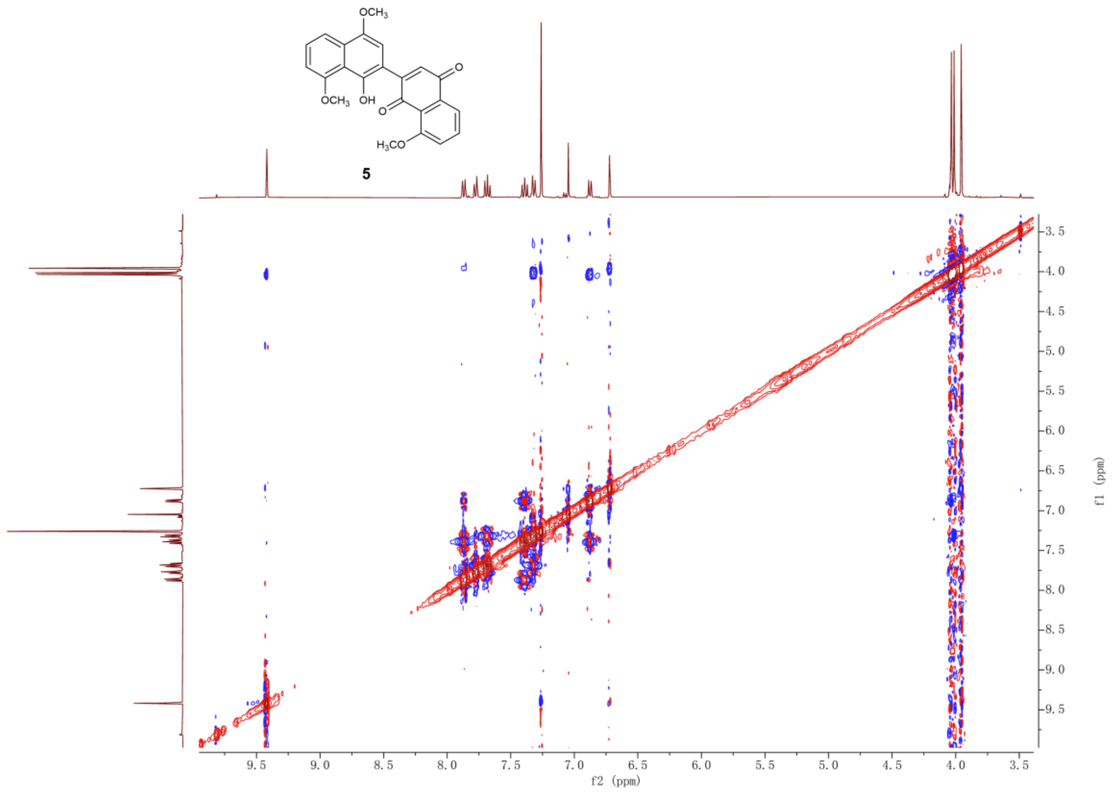

Figure S39. *NOSEY of compound* ***5***

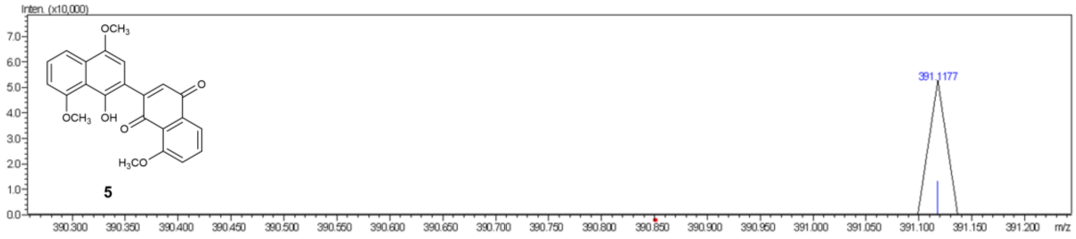

Figure S40. *HR-ESI-MS of compound* ***5***

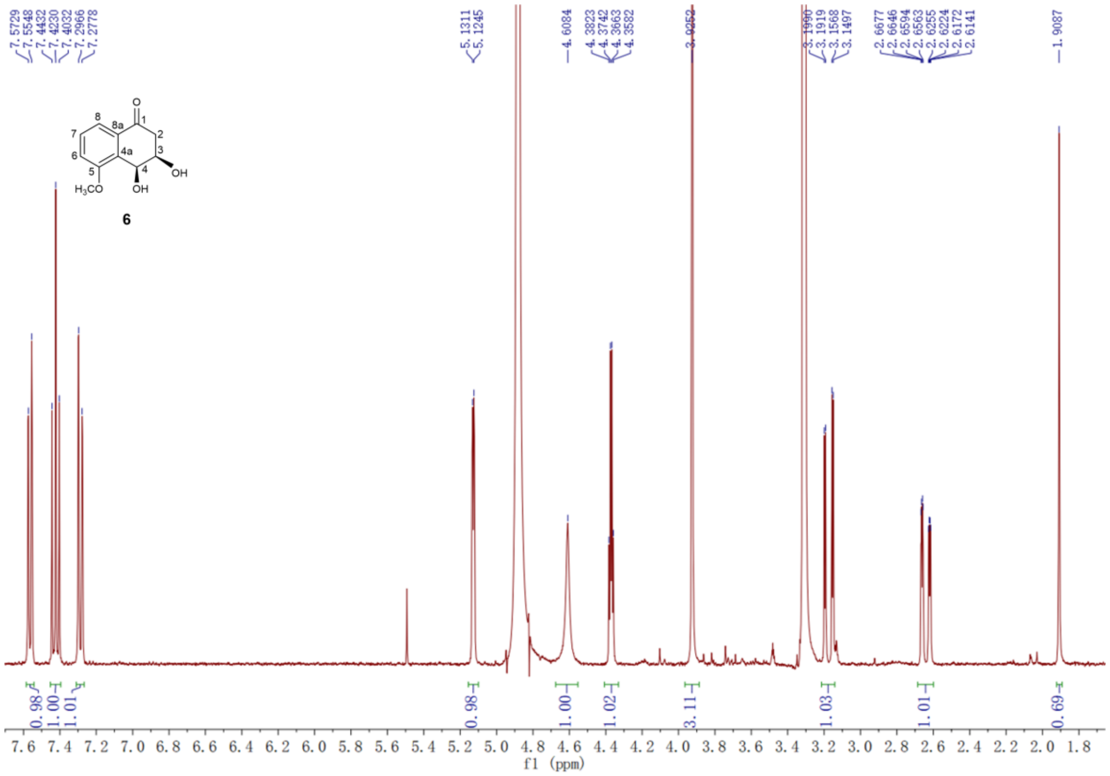

Figure S41. *^{1}H-NMR of compound* ***6***

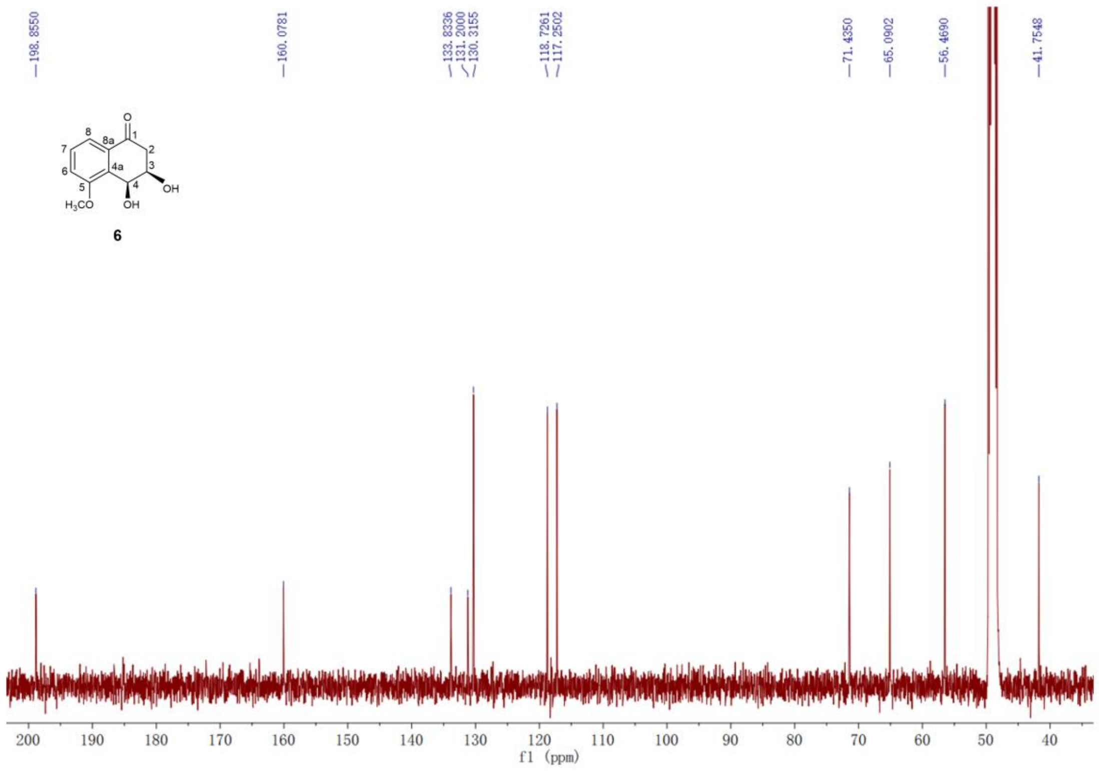

Figure S42. *^{13}C-NMR of compound* ***6***

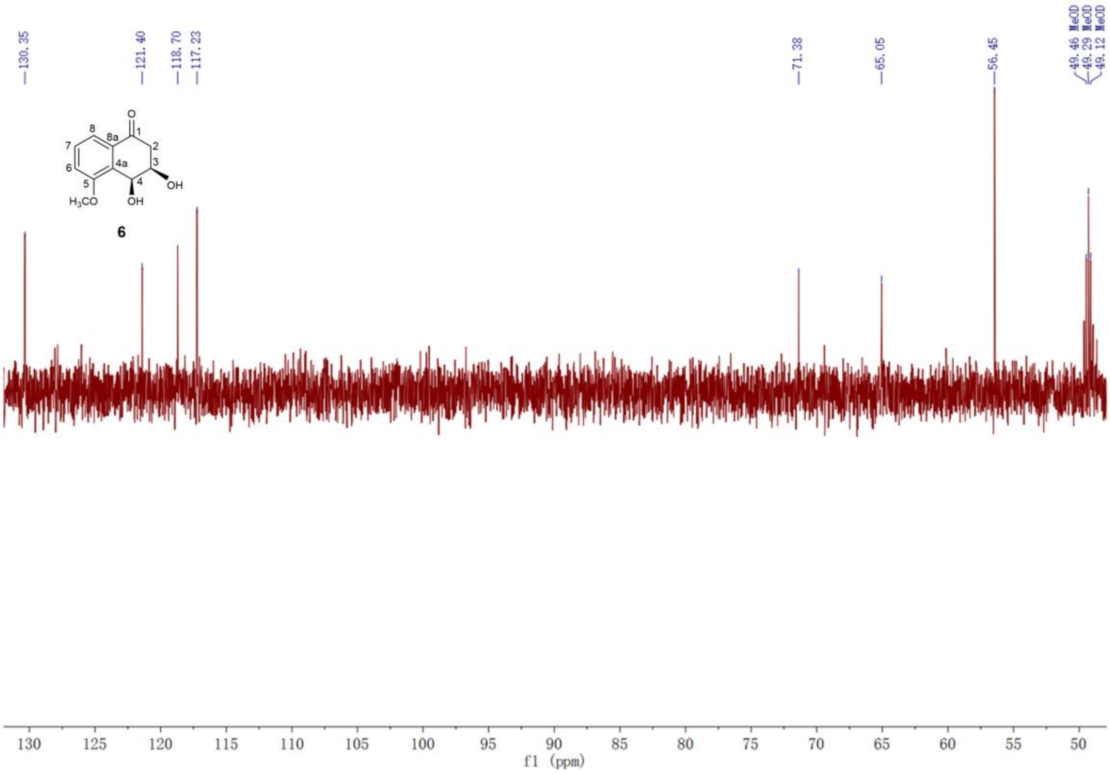

Figure S43. *DEPT of compound* ***6***

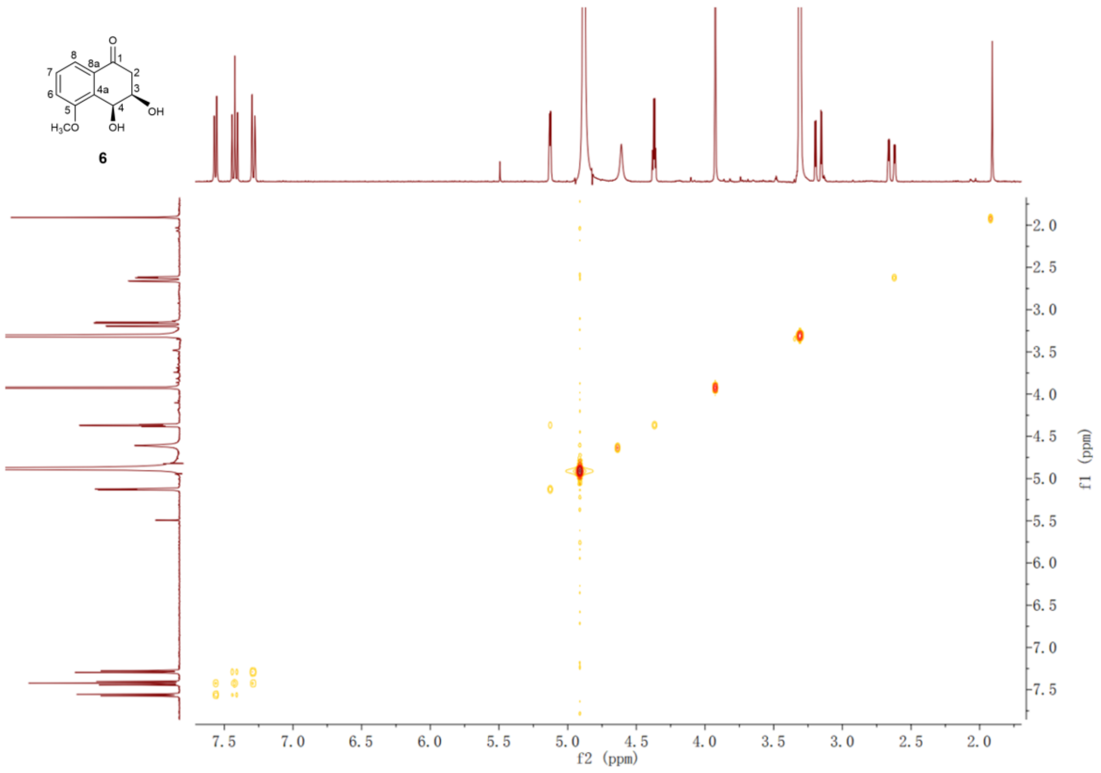

Figure S44. *^{1}H-^{1}H COSY of compound* ***6***

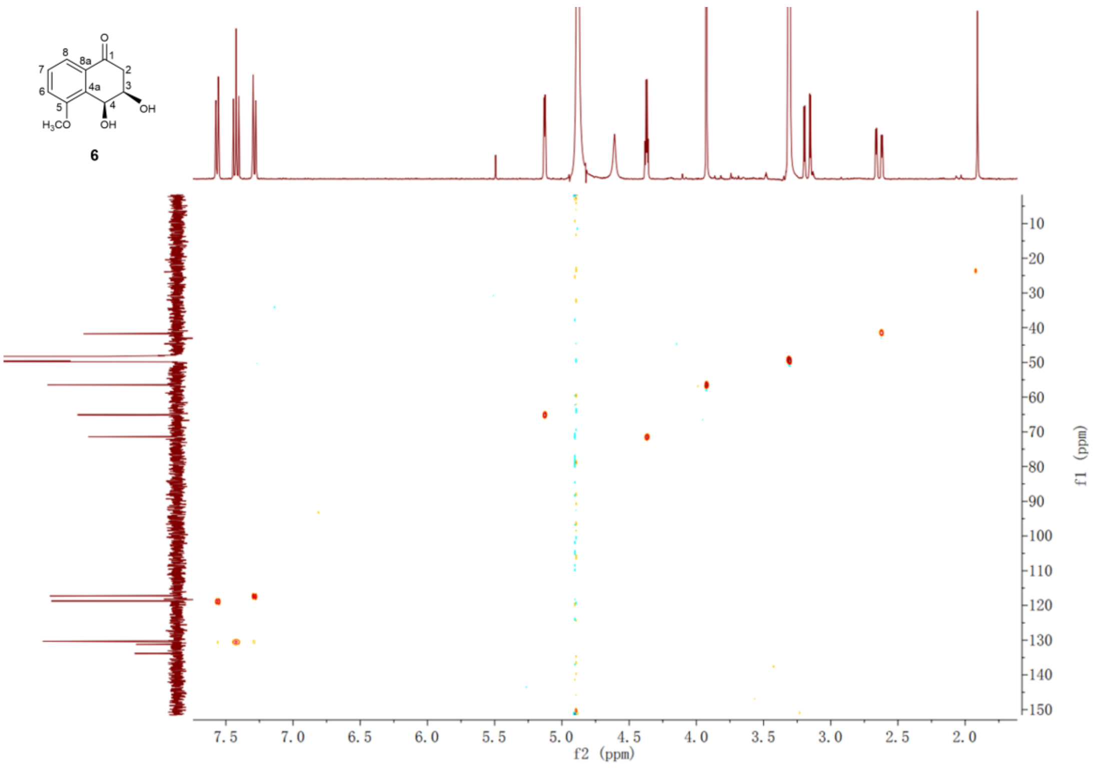

Figure S45. *HSQC of compound* ***6***

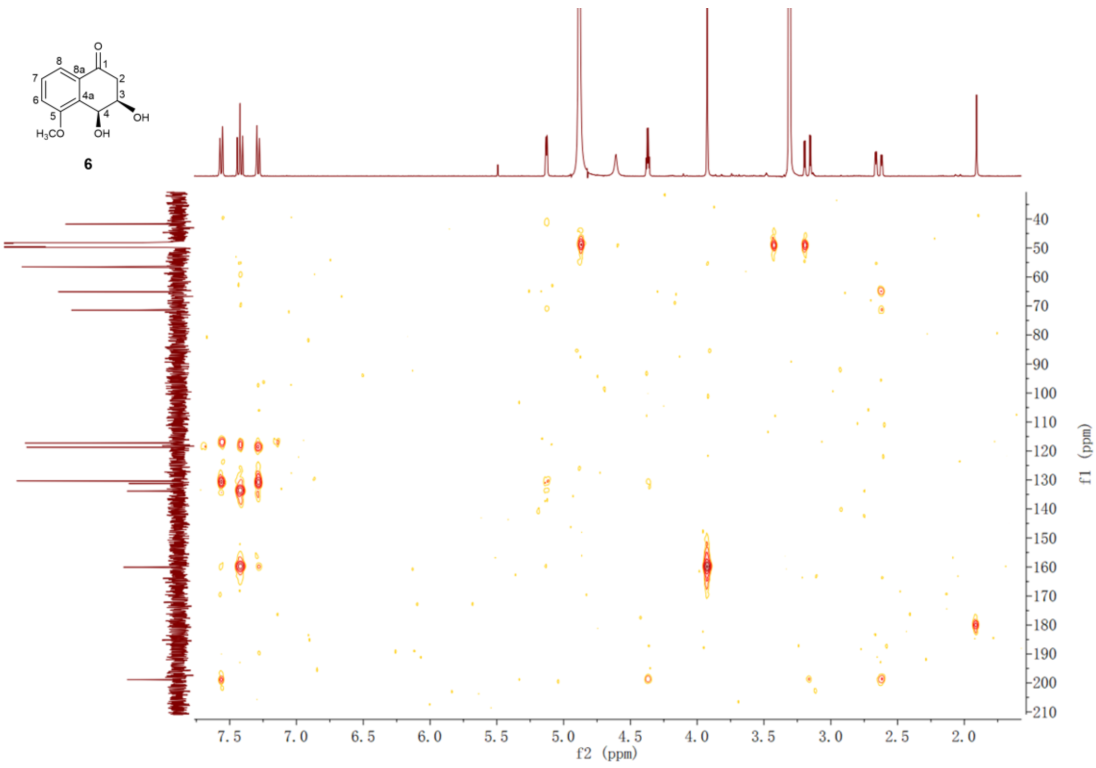

Figure S46. *HMBC of compound* ***6***

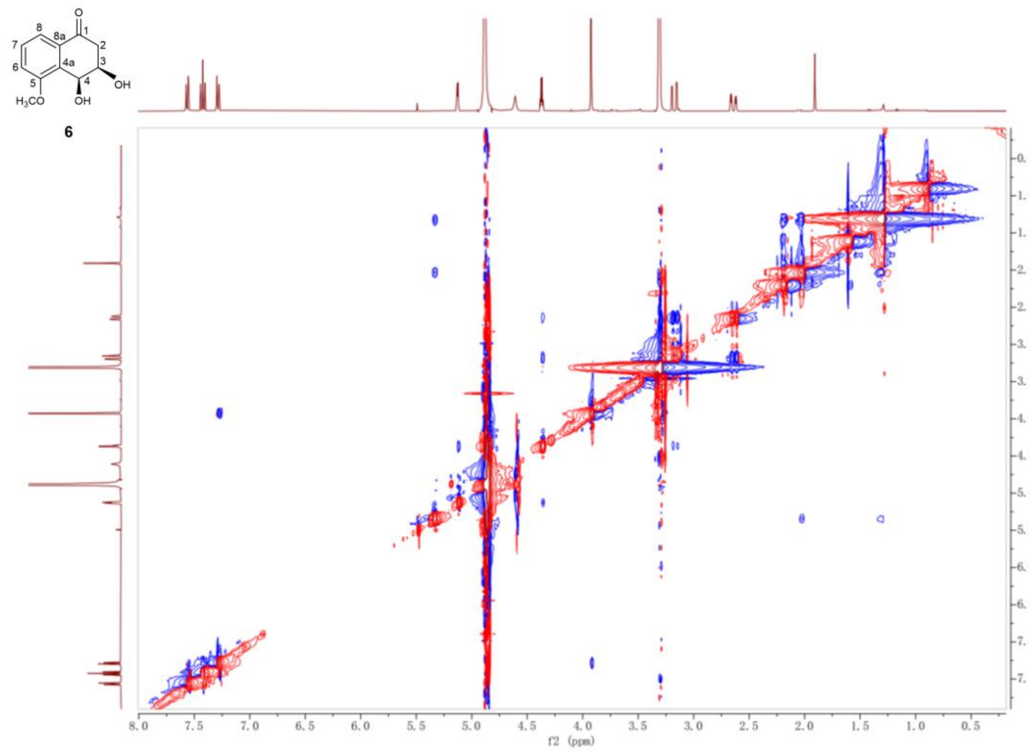

Figure S47. *NOESY of compound* ***6***

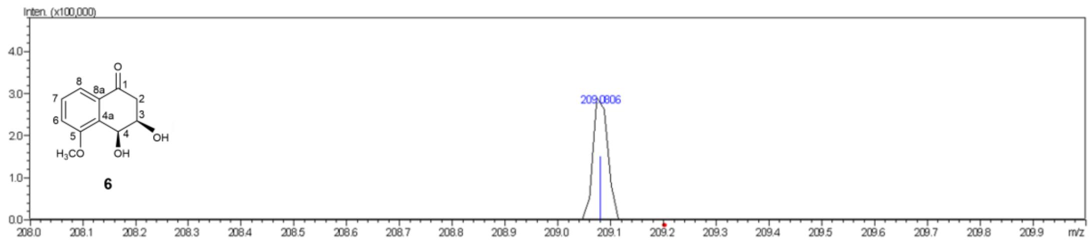

Figure S47. *HR-ESI-MS of compound* ***6***

Table S1. *Gibbs free energies[a] and equilibrium populations[b] of low-energy conformers of dalesconoside A (**1**)*

*Conformers of **1***	*In MeOH*	
	ΔG [a]	*P (%) [b]*

1-1	*-744279.56707494*	*58.70*
1-2	*-744278.51599569*	*9.94*
1-3	*-744278.45826477*	*9.02*
1-4	*-744278.55678384*	*10.65*
1-5	*-744278.61137721*	*11.68*

[a]ΔG, B3LYP/ 6-31g (d, p), in kcal/mol. [b] Boltzmann-population.

Table S2. *Cartesian coordinates for the low-energy reoptimized MMFF conformers of dalesconoside A (**1**) at B3LYP/6-31G(d,p) level of theory in gas*

*Conformer **1-1***		*Standard Orientation (Ångstroms)*			
Center Number	*Atom*	*Type*	*X*	*Y*	*Z*
1.	*6.*	*0.*	*-0.555849*	*3.082147*	*1.578182*
2.	*6.*	*0.*	*-0.352026*	*2.735590*	*2.928406*
3.	*6.*	*0.*	*-0.152146*	*1.422644*	*3.302851*
4.	*6.*	*0.*	*-0.155445*	*0.368197*	*2.332110*
5.	*6.*	*0.*	*-0.350845*	*0.745380*	*0.955911*
6.	*6.*	*0.*	*-0.550766*	*2.106569*	*0.609217*
7.	*6.*	*0.*	*0.015198*	*-1.024105*	*2.630892*
8.	*6.*	*0.*	*0.024991*	*-1.956553*	*1.618585*

9.	*6.*	*0.*	*-0.140365*	*-1.591193*	*0.265692*
10.	*6.*	*0.*	*-0.340846*	*-0.267162*	*-0.055535*
11.	*8.*	*0.*	*-0.005819*	*1.165100*	*4.649896*
12.	*6.*	*0.*	*1.347238*	*1.078913*	*5.104802*
13.	*8.*	*0.*	*0.235780*	*-1.474932*	*3.916918*
14.	*6.*	*0.*	*-0.948323*	*-1.719609*	*4.679505*
15.	*8.*	*0.*	*-0.525614*	*0.188105*	*-1.344972*
16.	*6.*	*0.*	*-0.944134*	*-0.737687*	*-2.338105*
17.	*8.*	*0.*	*0.131876*	*-1.546053*	*-2.776583*
18.	*6.*	*0.*	*0.667827*	*-1.069193*	*-4.029397*
19.	*6.*	*0.*	*-0.065172*	*0.249393*	*-4.315980*
20.	*6.*	*0.*	*-1.394517*	*0.071947*	*-3.554691*
21.	*8.*	*0.*	*-0.264121*	*0.380898*	*-5.722399*
22.	*8.*	*0.*	*-2.283569*	*-0.714819*	*-4.318660*
23.	*6.*	*0.*	*2.178484*	*-0.927544*	*-3.886865*
24.	*8.*	*0.*	*2.533411*	*0.030905*	*-2.907379*
25.	*1.*	*0.*	*-0.714959*	*4.121808*	*1.308207*
26.	*1.*	*0.*	*-0.356239*	*3.491419*	*3.707187*
27.	*1.*	*0.*	*-0.700710*	*2.357545*	*-0.433176*
28.	*1.*	*0.*	*0.183457*	*-2.996681*	*1.884045*
29.	*1.*	*0.*	*-0.083846*	*-2.347680*	*-0.507062*

30.	*1.*	*0.*	*1.854392*	*0.211339*	*4.672235*
31.	*1.*	*0.*	*1.898750*	*1.996297*	*4.862369*
32.	*1.*	*0.*	*1.298450*	*0.963364*	*6.189675*
33.	*1.*	*0.*	*-0.618477*	*-2.140860*	*5.631730*
34.	*1.*	*0.*	*-1.496809*	*-0.791490*	*4.867368*
35.	*1.*	*0.*	*-1.598380*	*-2.442092*	*4.169392*
36.	*1.*	*0.*	*-1.726551*	*-1.388114*	*-1.935946*
37.	*1.*	*0.*	*0.429036*	*-1.794738*	*-4.817667*
38.	*1.*	*0.*	*0.510613*	*1.085196*	*-3.905871*
39.	*1.*	*0.*	*-1.851952*	*1.021979*	*-3.246849*
40.	*1.*	*0.*	*-0.361549*	*1.319423*	*-5.929025*
41.	*1.*	*0.*	*-2.052976*	*-0.537623*	*-5.246183*
42.	*1.*	*0.*	*2.610329*	*-1.915186*	*-3.665356*
43.	*1.*	*0.*	*2.596264*	*-0.584449*	*-4.839412*
44.	*1.*	*0.*	*2.080912*	*-0.229394*	*-2.091065*

*Conformer **1-2***		*Standard Orientation (Ångstroms)*			
Center Number	*Atom*	*Type*	*X*	*Y*	*Z*
1.	*6.*	*0.*	*1.708436*	*2.839643*	*-1.140022*

2.	*6.*	*0.*	*1.648110*	*2.660823*	*-2.536144*
3.	*6.*	*0.*	*1.055380*	*1.541777*	*-3.084242*
4.	*6.*	*0.*	*0.490615*	*0.520879*	*-2.251487*
5.	*6.*	*0.*	*0.551611*	*0.728481*	*-0.826955*
6.	*6.*	*0.*	*1.167272*	*1.894100*	*-0.301125*
7.	*6.*	*0.*	*-0.120182*	*-0.685115*	*-2.730098*
8.	*6.*	*0.*	*-0.655435*	*-1.593607*	*-1.844989*
9.	*6.*	*0.*	*-0.619619*	*-1.385342*	*-0.450253*
10.	*6.*	*0.*	*-0.014428*	*-0.253240*	*0.045663*
11.	*8.*	*0.*	*1.082159*	*1.422264*	*-4.457669*
12.	*6.*	*0.*	*-0.094241*	*1.882840*	*-5.128633*
13.	*8.*	*0.*	*-0.252282*	*-0.953180*	*-4.077493*
14.	*6.*	*0.*	*0.874544*	*-1.597284*	*-4.676821*
15.	*8.*	*0.*	*0.078302*	*0.031513*	*1.397192*
16.	*6.*	*0.*	*0.015831*	*-1.056758*	*2.324129*
17.	*8.*	*0.*	*-1.304990*	*-1.494556*	*2.518194*
18.	*6.*	*0.*	*-1.777587*	*-1.127507*	*3.838558*
19.	*6.*	*0.*	*-0.507644*	*-1.038870*	*4.691710*
20.	*6.*	*0.*	*0.542086*	*-0.541112*	*3.682642*
21.	*8.*	*0.*	*-0.189801*	*-2.340025*	*5.149186*
22.	*8.*	*0.*	*1.811075*	*-1.067101*	*4.040959*

23.	*6.*	*0.*	*-2.602148*	*0.155692*	*3.757830*
24.	*8.*	*0.*	*-1.844080*	*1.283554*	*3.347780*
25.	*1.*	*0.*	*2.182182*	*3.727265*	*-0.731625*
26.	*1.*	*0.*	*2.074209*	*3.393943*	*-3.213405*
27.	*1.*	*0.*	*1.202646*	*2.022059*	*0.773517*
28.	*1.*	*0.*	*-1.132087*	*-2.480477*	*-2.249498*
29.	*1.*	*0.*	*-1.090814*	*-2.097338*	*0.216011*
30.	*1.*	*0.*	*-0.955893*	*1.252139*	*-4.890565*
31.	*1.*	*0.*	*-0.307945*	*2.926955*	*-4.866987*
32.	*1.*	*0.*	*0.117399*	*1.815047*	*-6.197836*
33.	*1.*	*0.*	*0.589030*	*-1.818496*	*-5.707564*
34.	*1.*	*0.*	*1.750029*	*-0.940709*	*-4.678618*
35.	*1.*	*0.*	*1.109893*	*-2.534885*	*-4.157003*
36.	*1.*	*0.*	*0.614019*	*-1.885044*	*1.928640*
37.	*1.*	*0.*	*-2.406333*	*-1.950554*	*4.193467*
38.	*1.*	*0.*	*-0.623567*	*-0.336168*	*5.529052*
39.	*1.*	*0.*	*0.544185*	*0.552392*	*3.663282*
40.	*1.*	*0.*	*0.773817*	*-2.366444*	*5.252171*
41.	*1.*	*0.*	*2.496284*	*-0.583286*	*3.564334*
42.	*1.*	*0.*	*-3.454805*	*-0.022977*	*3.086319*
43.	*1.*	*0.*	*-3.003532*	*0.392012*	*4.749544*

44.	*1.*	*0.*	*-1.521701*	*1.110696*	*2.448853*

*Conformer **1-3***		*Standard Orientation (Ångstroms)*			
Center Number	*Atom*	*Type*	*X*	*Y*	*Z*
1.	*6.*	*0.*	*1.696275*	*3.056515*	*0.129991*
2.	*6.*	*0.*	*2.287625*	*3.101969*	*-1.147850*
3.	*6.*	*0.*	*2.139611*	*2.056930*	*-2.036676*
4.	*6.*	*0.*	*1.369061*	*0.897948*	*-1.694269*
5.	*6.*	*0.*	*0.774636*	*0.869785*	*-0.382121*
6.	*6.*	*0.*	*0.958647*	*1.959467*	*0.508363*
7.	*6.*	*0.*	*1.153304*	*-0.228308*	*-2.555426*
8.	*6.*	*0.*	*0.401602*	*-1.298882*	*-2.126741*
9.	*6.*	*0.*	*-0.190615*	*-1.328899*	*-0.846741*
10.	*6.*	*0.*	*-0.000571*	*-0.267224*	*0.007871*
11.	*8.*	*0.*	*2.711202*	*2.201745*	*-3.283187*
12.	*6.*	*0.*	*4.016788*	*1.634563*	*-3.420737*
13.	*8.*	*0.*	*1.725289*	*-0.309418*	*-3.808783*
14.	*6.*	*0.*	*0.960546*	*0.285750*	*-4.860039*
15.	*8.*	*0.*	*-0.538462*	*-0.208309*	*1.282054*

16.	*6.*	*0.*	*-0.890151*	*-1.437524*	*1.926658*
17.	*8.*	*0.*	*-2.106748*	*-1.945285*	*1.442715*
18.	*6.*	*0.*	*-3.153737*	*-1.790557*	*2.433448*
19.	*6.*	*0.*	*-2.416925*	*-1.804909*	*3.776806*
20.	*6.*	*0.*	*-1.073498*	*-1.136414*	*3.431877*
21.	*8.*	*0.*	*-2.226175*	*-3.152791*	*4.163264*
22.	*8.*	*0.*	*-0.058047*	*-1.693003*	*4.253130*
23.	*6.*	*0.*	*-3.965034*	*-0.528092*	*2.148495*
24.	*8.*	*0.*	*-3.213997*	*0.667878*	*2.292800*
25.	*1.*	*0.*	*1.826328*	*3.890868*	*0.812652*
26.	*1.*	*0.*	*2.865828*	*3.963213*	*-1.466752*
27.	*1.*	*0.*	*0.501042*	*1.911672*	*1.488591*
28.	*1.*	*0.*	*0.268585*	*-2.134694*	*-2.805830*
29.	*1.*	*0.*	*-0.813604*	*-2.166346*	*-0.558087*
30.	*1.*	*0.*	*4.701132*	*2.043987*	*-2.666702*
31.	*1.*	*0.*	*3.982469*	*0.543711*	*-3.343390*
32.	*1.*	*0.*	*4.368434*	*1.914151*	*-4.416124*
33.	*1.*	*0.*	*1.492069*	*0.065704*	*-5.788456*
34.	*1.*	*0.*	*-0.045287*	*-0.150911*	*-4.906141*
35.	*1.*	*0.*	*0.891732*	*1.370416*	*-4.732309*
36.	*1.*	*0.*	*-0.096177*	*-2.168124*	*1.737407*

37.	*1.*	*0.*	*-3.799644*	*-2.670945*	*2.354356*
38.	*1.*	*0.*	*-2.958657*	*-1.238813*	*4.547784*
39.	*1.*	*0.*	*-1.157364*	*-0.054107*	*3.563805*
40.	*1.*	*0.*	*-1.411738*	*-3.174980*	*4.688681*
41.	*1.*	*0.*	*0.719299*	*-1.122684*	*4.219733*
42.	*1.*	*0.*	*-4.397033*	*-0.614549*	*1.140590*
43.	*1.*	*0.*	*-4.794643*	*-0.463785*	*2.861339*
44.	*1.*	*0.*	*-2.502395*	*0.651282*	*1.632951*

*Conformer **1-4***		*Standard Orientation (Ångstroms)*			
Center Number	*Atom*	*Type*	*X*	*Y*	*Z*
1.	*6.*	*0.*	*-2.194350*	*-2.625564*	*-1.123485*
2.	*6.*	*0.*	*-1.766449*	*-3.717567*	*-0.342876*
3.	*6.*	*0.*	*-0.642226*	*-3.625517*	*0.451695*
4.	*6.*	*0.*	*0.132238*	*-2.420612*	*0.502001*
5.	*6.*	*0.*	*-0.330440*	*-1.311445*	*-0.291955*
6.	*6.*	*0.*	*-1.492770*	*-1.443294*	*-1.095033*
7.	*6.*	*0.*	*1.327774*	*-2.243192*	*1.273427*
8.	*6.*	*0.*	*1.983972*	*-1.033387*	*1.269416*

9.	*6.*	*0.*	*1.522851*	*0.064661*	*0.512969*
10.	*6.*	*0.*	*0.394225*	*-0.077096*	*-0.263123*
11.	*8.*	*0.*	*-0.267036*	*-4.756511*	*1.147621*
12.	*6.*	*0.*	*-0.725373*	*-4.812490*	*2.500463*
13.	*8.*	*0.*	*1.829095*	*-3.240369*	*2.087588*
14.	*6.*	*0.*	*2.699439*	*-4.166793*	*1.434537*
15.	*8.*	*0.*	*-0.129416*	*0.925494*	*-1.047780*
16.	*6.*	*0.*	*0.689572*	*2.033739*	*-1.378495*
17.	*8.*	*0.*	*0.851711*	*2.908473*	*-0.269598*
18.	*6.*	*0.*	*0.051053*	*4.081894*	*-0.443223*
19.	*6.*	*0.*	*-0.914067*	*3.759765*	*-1.596004*
20.	*6.*	*0.*	*-0.048824*	*2.832802*	*-2.458516*
21.	*8.*	*0.*	*-1.309536*	*4.895369*	*-2.340690*
22.	*8.*	*0.*	*0.899000*	*3.586491*	*-3.187789*
23.	*6.*	*0.*	*-0.659857*	*4.444257*	*0.849653*
24.	*8.*	*0.*	*-1.451771*	*5.592936*	*0.525587*
25.	*1.*	*0.*	*-3.078909*	*-2.723093*	*-1.745768*
26.	*1.*	*0.*	*-2.304376*	*-4.660113*	*-0.355364*
27.	*1.*	*0.*	*-1.810617*	*-0.594260*	*-1.686877*
28.	*1.*	*0.*	*2.870862*	*-0.932088*	*1.886573*
29.	*1.*	*0.*	*2.039040*	*1.014365*	*0.572811*

30.	*1.*	*0.*	*-0.257478*	*-4.032407*	*3.108438*
31.	*1.*	*0.*	*-1.818159*	*-4.719657*	*2.545909*
32.	*1.*	*0.*	*-0.430718*	*-5.792006*	*2.883369*
33.	*1.*	*0.*	*3.075606*	*-4.835644*	*2.211988*
34.	*1.*	*0.*	*2.161065*	*-4.754100*	*0.684221*
35.	*1.*	*0.*	*3.543864*	*-3.645088*	*0.965884*
36.	*1.*	*0.*	*1.678633*	*1.688390*	*-1.695264*
37.	*1.*	*0.*	*0.690847*	*4.919770*	*-0.750829*
38.	*1.*	*0.*	*-1.781927*	*3.202570*	*-1.215000*
39.	*1.*	*0.*	*-0.634702*	*2.170911*	*-3.108172*
40.	*1.*	*0.*	*-1.615659*	*5.547031*	*-1.690609*
41.	*1.*	*0.*	*0.443744*	*4.408549*	*-3.431813*
42.	*1.*	*0.*	*-1.282664*	*3.598709*	*1.174154*
43.	*1.*	*0.*	*0.065267*	*4.664909*	*1.644673*
44.	*1.*	*0.*	*-2.003534*	*5.817209*	*1.284285*

Conformer ***1-5***		*Standard Orientation (Ångstroms)*			
Center Number	*Atom*	*Type*	*X*	*Y*	*Z*
1.	*6.*	*0.*	*2.494561*	*2.577554*	*0.221603*

2.	6.	0.	2.928708	2.685413	-1.114326
3.	6.	0.	2.500509	1.791815	-2.074451
4.	6.	0.	1.590001	0.734210	-1.748014
5.	6.	0.	1.161089	0.639212	-0.376365
6.	6.	0.	1.631968	1.570308	0.585224
7.	6.	0.	1.083459	-0.231270	-2.679422
8.	6.	0.	0.218402	-1.215446	-2.258763
9.	6.	0.	-0.210744	-1.310557	-0.918115
10.	6.	0.	0.256482	-0.402612	0.005714
11.	8.	0.	2.938459	1.994855	-3.367161
12.	6.	0.	4.097177	1.242958	-3.736081
13.	8.	0.	1.475870	-0.246559	-4.003990
14.	6.	0.	0.703799	0.588621	-4.868734
15.	8.	0.	-0.092827	-0.412128	1.337094
16.	6.	0.	-0.673454	-1.583207	1.883930
17.	8.	0.	-2.026764	-1.730386	1.473937
18.	6.	0.	-2.909197	-1.392089	2.548929
19.	6.	0.	-2.035485	-0.684680	3.598039
20.	6.	0.	-0.696121	-1.406742	3.406291
21.	8.	0.	-2.496003	-0.848960	4.925289
22.	8.	0.	-0.739388	-2.685841	4.006437

23.	*6.*	*0.*	*-4.058480*	*-0.533498*	*2.049315*
24.	*8.*	*0.*	*-4.829155*	*-0.231188*	*3.218019*
25.	*1.*	*0.*	*2.846481*	*3.290960*	*0.960794*
26.	*1.*	*0.*	*3.604354*	*3.477234*	*-1.421621*
27.	*1.*	*0.*	*1.293712*	*1.471628*	*1.608972*
28.	*1.*	*0.*	*-0.135429*	*-1.931869*	*-2.993113*
29.	*1.*	*0.*	*-0.926384*	*-2.071997*	*-0.635105*
30.	*1.*	*0.*	*4.929595*	*1.447946*	*-3.050661*
31.	*1.*	*0.*	*3.881585*	*0.170438*	*-3.753733*
32.	*1.*	*0.*	*4.368722*	*1.572151*	*-4.741454*
33.	*1.*	*0.*	*1.072242*	*0.407480*	*-5.881061*
34.	*1.*	*0.*	*-0.360651*	*0.324954*	*-4.818706*
35.	*1.*	*0.*	*0.837943*	*1.646183*	*-4.620677*
36.	*1.*	*0.*	*-0.114361*	*-2.466560*	*1.560076*
37.	*1.*	*0.*	*-3.311570*	*-2.311036*	*2.995791*
38.	*1.*	*0.*	*-1.920676*	*0.377421*	*3.337854*
39.	*1.*	*0.*	*0.166184*	*-0.821478*	*3.748896*
40.	*1.*	*0.*	*-3.435729*	*-0.608720*	*4.913599*
41.	*1.*	*0.*	*-1.269412*	*-2.576150*	*4.812413*
42.	*1.*	*0.*	*-3.657715*	*0.375683*	*1.579229*
43.	*1.*	*0.*	*-4.653584*	*-1.075895*	*1.302164*

44.	*1.*	*0.*	*-5.527174*	*0.390846*	*2.980943*

Table S3. *Gibbs free energies[a] and equilibrium populations[b] of low-energy conformers of dalesconoside B (**2**)*

*Conformers of **2***	*In MeOH*	
	ΔG [a]	*P (%) [b]*
2-1	*-722512.40857848*	*90.46*
2-2	*-722510.94271512*	*7.60*
2-3	*-722510.05165092*	*1.69*
2-4	*-722508.92652549*	*0.25*

[a]ΔG, B3LYP/ 6-31g (d, p), in kcal/mol. [b] Boltzmann-population.

Table S4. *Cartesian coordinates for the low-energy reoptimized MMFF conformers of dalesconoside B (**2**) at B3LYP/6-31G(d,p) level of theory in gas*

1.2

*Conformer **2-1***		*Standard Orientation (Ångstroms)*			
Center Number	*Atom*	*Type*	*X*	*Y*	*Z*
1.	*6.*	*0.*	*1.268382*	*4.924338*	*-0.190601*
2.	*6.*	*0.*	*1.305173*	*4.005234*	*0.862338*

3.	6.	0.	1.030912	2.658126	0.623370
4.	6.	0.	0.737413	2.190187	-0.678248
5.	6.	0.	0.682905	3.143455	-1.713557
6.	6.	0.	0.952292	4.499867	-1.469509
7.	6.	0.	0.449843	0.701776	-0.943653
8.	6.	0.	0.694099	0.374930	-2.423653
9.	6.	0.	-0.054065	1.313953	-3.367509
10.	6.	0.	0.305780	2.765771	-3.115965
11.	8.	0.	0.268827	3.598352	-4.008348
12.	8.	0.	1.414368	-0.119073	-0.250588
13.	8.	0.	1.022987	1.714898	1.628606
14.	6.	0.	1.290062	2.120302	2.966940
15.	6.	0.	-0.994705	0.387246	-0.462634
16.	6.	0.	-1.593210	-1.005909	-0.742151
17.	6.	0.	-0.967365	-2.204057	-0.018957
18.	8.	0.	-0.908732	-1.913430	1.368940
19.	6.	0.	-1.025085	-3.134457	2.150652
20.	6.	0.	-1.294988	-4.260459	1.139274
21.	6.	0.	-1.791102	-3.510129	-0.110590
22.	8.	0.	0.301853	-2.556043	-0.535114
23.	6.	0.	0.225603	-3.321377	2.998656

24.	*8.*	*0.*	*-1.623904*	*-4.186622*	*-1.334256*
25.	*1.*	*0.*	*1.480914*	*5.970454*	*0.007178*
26.	*1.*	*0.*	*1.544692*	*4.348447*	*1.861254*
27.	*1.*	*0.*	*0.898147*	*5.186874*	*-2.306126*
28.	*1.*	*0.*	*1.772866*	*0.460344*	*-2.595369*
29.	*1.*	*0.*	*0.429130*	*-0.669141*	*-2.607712*
30.	*1.*	*0.*	*0.152732*	*1.092321*	*-4.417847*
31.	*1.*	*0.*	*-1.140962*	*1.215099*	*-3.236401*
32.	*1.*	*0.*	*1.347964*	*0.105771*	*0.692866*
33.	*1.*	*0.*	*1.206263*	*1.217573*	*3.572461*
34.	*1.*	*0.*	*0.556422*	*2.857789*	*3.311435*
35.	*1.*	*0.*	*2.300182*	*2.534452*	*3.066217*
36.	*1.*	*0.*	*-1.028615*	*0.569686*	*0.615563*
37.	*1.*	*0.*	*-1.662599*	*1.129880*	*-0.914884*
38.	*1.*	*0.*	*-1.598240*	*-1.237814*	*-1.812511*
39.	*1.*	*0.*	*-2.643833*	*-0.956522*	*-0.433528*
40.	*1.*	*0.*	*-1.894364*	*-2.995351*	*2.807907*
41.	*1.*	*0.*	*-0.368275*	*-4.784415*	*0.884589*
42.	*1.*	*0.*	*-2.014851*	*-4.995425*	*1.508079*
43.	*1.*	*0.*	*-2.857337*	*-3.275908*	*-0.016716*
44.	*1.*	*0.*	*0.879842*	*-1.765695*	*-0.450986*

45.	*1.*	*0.*	*1.101837*	*-3.430686*	*2.352786*
46.	*1.*	*0.*	*0.381728*	*-2.459235*	*3.654809*
47.	*1.*	*0.*	*0.137869*	*-4.215472*	*3.625823*
48.	*1.*	*0.*	*-0.679857*	*-4.090905*	*-1.541330*

Conformer ***2-2***		*Standard Orientation (Ångstroms)*			
Center Number	*Atom*	*Type*	*X*	*Y*	*Z*
1.	*6.*	*0.*	*0.674571*	*5.026083*	*-0.226703*
2.	*6.*	*0.*	*0.814313*	*4.116085*	*0.825522*
3.	*6.*	*0.*	*0.733497*	*2.744870*	*0.579143*
4.	*6.*	*0.*	*0.536511*	*2.247157*	*-0.729894*
5.	*6.*	*0.*	*0.372934*	*3.188425*	*-1.764587*
6.	*6.*	*0.*	*0.447229*	*4.568041*	*-1.512799*
7.	*6.*	*0.*	*0.468734*	*0.734045*	*-1.003196*
8.	*6.*	*0.*	*0.787694*	*0.452199*	*-2.478654*
9.	*6.*	*0.*	*-0.063455*	*1.281769*	*-3.437674*
10.	*6.*	*0.*	*0.081774*	*2.768687*	*-3.175125*
11.	*8.*	*0.*	*-0.055109*	*3.591694*	*-4.066727*
12.	*8.*	*0.*	*1.526740*	*0.056614*	*-0.291775*

13.	*8.*	*0.*	*0.831093*	*1.805937*	*1.581911*
14.	*6.*	*0.*	*1.027830*	*2.236778*	*2.924275*
15.	*6.*	*0.*	*-0.925739*	*0.215482*	*-0.551374*
16.	*6.*	*0.*	*-1.285844*	*-1.260900*	*-0.804875*
17.	*6.*	*0.*	*-0.455065*	*-2.317010*	*-0.053076*
18.	*8.*	*0.*	*-0.328513*	*-1.899298*	*1.312809*
19.	*6.*	*0.*	*-0.829654*	*-2.899725*	*2.227246*
20.	*6.*	*0.*	*-1.794746*	*-3.737935*	*1.372187*
21.	*6.*	*0.*	*-1.150171*	*-3.716170*	*-0.016749*
22.	*8.*	*0.*	*0.807418*	*-2.502062*	*-0.641347*
23.	*6.*	*0.*	*0.316140*	*-3.688199*	*2.857866*
24.	*8.*	*0.*	*-0.210185*	*-4.767016*	*-0.132466*
25.	*1.*	*0.*	*0.737095*	*6.090612*	*-0.022888*
26.	*1.*	*0.*	*0.981854*	*4.484329*	*1.830136*
27.	*1.*	*0.*	*0.317165*	*5.244842*	*-2.349379*
28.	*1.*	*0.*	*1.847102*	*0.690319*	*-2.625848*
29.	*1.*	*0.*	*0.678019*	*-0.617748*	*-2.672649*
30.	*1.*	*0.*	*0.197225*	*1.097241*	*-4.483211*
31.	*1.*	*0.*	*-1.128427*	*1.030090*	*-3.333219*
32.	*1.*	*0.*	*1.384913*	*0.227211*	*0.654705*
33.	*1.*	*0.*	*1.058751*	*1.327767*	*3.525365*

34.	*1.*	*0.*	*0.199631*	*2.868767*	*3.264671*
35.	*1.*	*0.*	*1.972975*	*2.780823*	*3.035843*
36.	*1.*	*0.*	*-1.016017*	*0.412548*	*0.520473*
37.	*1.*	*0.*	*-1.686197*	*0.835311*	*-1.040649*
38.	*1.*	*0.*	*-1.250324*	*-1.503626*	*-1.872774*
39.	*1.*	*0.*	*-2.332330*	*-1.382610*	*-0.497048*
40.	*1.*	*0.*	*-1.367198*	*-2.351205*	*3.010654*
41.	*1.*	*0.*	*-1.920161*	*-4.762196*	*1.730609*
42.	*1.*	*0.*	*-2.779677*	*-3.259233*	*1.338848*
43.	*1.*	*0.*	*-1.899270*	*-3.791937*	*-0.818838*
44.	*1.*	*0.*	*1.295753*	*-1.655279*	*-0.531047*
45.	*1.*	*0.*	*0.825267*	*-4.291498*	*2.104015*
46.	*1.*	*0.*	*1.036124*	*-3.004470*	*3.318158*
47.	*1.*	*0.*	*-0.066202*	*-4.357873*	*3.636571*
48.	*1.*	*0.*	*0.583936*	*-4.357739*	*-0.518440*

Conformer ***2-3***		*Standard Orientation (Ångstroms)*			
Center Number	*Atom*	*Type*	*X*	*Y*	*Z*
1.	*6.*	*0.*	*1.070920*	*4.970452*	*-0.150944*

2.	*6.*	*0.*	*0.936357*	*4.062666*	*0.904204*
3.	*6.*	*0.*	*0.738197*	*2.707234*	*0.636534*
4.	*6.*	*0.*	*0.627355*	*2.231046*	*-0.690762*
5.	*6.*	*0.*	*0.863864*	*3.153167*	*-1.726488*
6.	*6.*	*0.*	*1.058371*	*4.517504*	*-1.459829*
7.	*6.*	*0.*	*0.265164*	*0.760798*	*-0.969250*
8.	*6.*	*0.*	*0.231055*	*0.447688*	*-2.477109*
9.	*6.*	*0.*	*1.284379*	*1.201122*	*-3.290008*
10.	*6.*	*0.*	*1.067679*	*2.690425*	*-3.137773*
11.	*8.*	*0.*	*1.116722*	*3.472098*	*-4.073492*
12.	*8.*	*0.*	*1.316787*	*-0.071583*	*-0.410411*
13.	*8.*	*0.*	*0.671301*	*1.753287*	*1.625790*
14.	*6.*	*0.*	*0.756272*	*2.156033*	*2.988592*
15.	*6.*	*0.*	*-1.114333*	*0.421214*	*-0.337623*
16.	*6.*	*0.*	*-1.729886*	*-0.965835*	*-0.618506*
17.	*6.*	*0.*	*-1.039944*	*-2.186571*	*0.000969*
18.	*8.*	*0.*	*-0.866979*	*-1.955335*	*1.390274*
19.	*6.*	*0.*	*-0.907549*	*-3.210371*	*2.124368*
20.	*6.*	*0.*	*-1.260266*	*-4.293937*	*1.092283*
21.	*6.*	*0.*	*-1.862092*	*-3.494712*	*-0.078428*
22.	*8.*	*0.*	*0.185596*	*-2.507932*	*-0.631405*

23.	*6.*	*0.*	*0.414424*	*-3.423809*	*2.849156*
24.	*8.*	*0.*	*-1.795001*	*-4.117326*	*-1.339540*
25.	*1.*	*0.*	*1.218840*	*6.023650*	*0.067386*
26.	*1.*	*0.*	*0.995201*	*4.418051*	*1.925664*
27.	*1.*	*0.*	*1.223225*	*5.182502*	*-2.300095*
28.	*1.*	*0.*	*0.347440*	*-0.632412*	*-2.595342*
29.	*1.*	*0.*	*-0.762064*	*0.703448*	*-2.863976*
30.	*1.*	*0.*	*2.286759*	*0.958288*	*-2.912971*
31.	*1.*	*0.*	*1.247936*	*0.942931*	*-4.350938*
32.	*1.*	*0.*	*1.339166*	*0.127700*	*0.540543*
33.	*1.*	*0.*	*0.664948*	*1.240753*	*3.573826*
34.	*1.*	*0.*	*-0.058973*	*2.838816*	*3.253857*
35.	*1.*	*0.*	*1.718517*	*2.634772*	*3.205128*
36.	*1.*	*0.*	*-1.052041*	*0.552933*	*0.742950*
37.	*1.*	*0.*	*-1.820463*	*1.175393*	*-0.706403*
38.	*1.*	*0.*	*-1.831918*	*-1.159816*	*-1.690945*
39.	*1.*	*0.*	*-2.747888*	*-0.934751*	*-0.213594*
40.	*1.*	*0.*	*-1.715637*	*-3.107005*	*2.861436*
41.	*1.*	*0.*	*-0.357261*	*-4.801409*	*0.738504*
42.	*1.*	*0.*	*-1.944719*	*-5.047992*	*1.488889*
43.	*1.*	*0.*	*-2.917762*	*-3.272847*	*0.113512*

44.	*1.*	*0.*	*0.764152*	*-1.716269*	*-0.569113*
45.	*1.*	*0.*	*1.229789*	*-3.502015*	*2.123871*
46.	*1.*	*0.*	*0.626740*	*-2.587729*	*3.522955*
47.	*1.*	*0.*	*0.385128*	*-4.342971*	*3.444774*
48.	*1.*	*0.*	*-0.871510*	*-4.008343*	*-1.619520*

Conformer ***2-4***		*Standard Orientation (Ångstroms)*			
Center Number	*Atom*	*Type*	*X*	*Y*	*Z*
1.	*6.*	*0.*	*2.707385*	*4.353249*	*-0.354607*
2.	*6.*	*0.*	*2.601996*	*3.505628*	*0.752700*
3.	*6.*	*0.*	*2.212653*	*2.176888*	*0.580737*
4.	*6.*	*0.*	*1.939050*	*1.651835*	*-0.703881*
5.	*6.*	*0.*	*2.028653*	*2.537227*	*-1.795550*
6.	*6.*	*0.*	*2.416345*	*3.876235*	*-1.620459*
7.	*6.*	*0.*	*1.524291*	*0.178265*	*-0.885365*
8.	*6.*	*0.*	*1.779894*	*-0.259821*	*-2.335829*
9.	*6.*	*0.*	*1.172591*	*0.689828*	*-3.365851*
10.	*6.*	*0.*	*1.680513*	*2.107883*	*-3.189215*
11.	*8.*	*0.*	*1.777132*	*2.875426*	*-4.134741*

12.	*8.*	*0.*	*2.356875*	*-0.696041*	*-0.119312*
13.	*8.*	*0.*	*2.066616*	*1.306867*	*1.639082*
14.	*6.*	*0.*	*2.215788*	*1.795232*	*2.967274*
15.	*6.*	*0.*	*0.040578*	*0.026482*	*-0.446637*
16.	*6.*	*0.*	*-0.499224*	*-1.406390*	*-0.556557*
17.	*6.*	*0.*	*-1.798128*	*-1.641262*	*0.215224*
18.	*8.*	*0.*	*-2.235851*	*-2.948741*	*-0.074977*
19.	*6.*	*0.*	*-2.985411*	*-3.510775*	*1.038295*
20.	*6.*	*0.*	*-2.929062*	*-2.456661*	*2.154123*
21.	*6.*	*0.*	*-1.728718*	*-1.577722*	*1.760401*
22.	*8.*	*0.*	*-2.801401*	*-0.677970*	*-0.127500*
23.	*6.*	*0.*	*-4.385471*	*-3.885699*	*0.574744*
24.	*8.*	*0.*	*-1.747943*	*-0.268963*	*2.290459*
25.	*1.*	*0.*	*3.009778*	*5.385869*	*-0.209333*
26.	*1.*	*0.*	*2.823794*	*3.889335*	*1.741017*
27.	*1.*	*0.*	*2.470188*	*4.508282*	*-2.499430*
28.	*1.*	*0.*	*2.866788*	*-0.302446*	*-2.465845*
29.	*1.*	*0.*	*1.411149*	*-1.279731*	*-2.473226*
30.	*1.*	*0.*	*1.386764*	*0.380204*	*-4.392405*
31.	*1.*	*0.*	*0.077498*	*0.720618*	*-3.270742*
32.	*1.*	*0.*	*2.329127*	*-0.376652*	*0.795413*

33.	*1.*	*0.*	*1.980511*	*0.955244*	*3.621155*
34.	*1.*	*0.*	*1.517730*	*2.615594*	*3.167958*
35.	*1.*	*0.*	*3.241702*	*2.131514*	*3.159008*
36.	*1.*	*0.*	*-0.031862*	*0.358234*	*0.592920*
37.	*1.*	*0.*	*-0.588284*	*0.713247*	*-1.024118*
38.	*1.*	*0.*	*0.251651*	*-2.109235*	*-0.182996*
39.	*1.*	*0.*	*-0.687956*	*-1.681364*	*-1.602731*
40.	*1.*	*0.*	*-2.445754*	*-4.417738*	*1.341910*
41.	*1.*	*0.*	*-3.831254*	*-1.835992*	*2.148838*
42.	*1.*	*0.*	*-2.823634*	*-2.897565*	*3.148375*
43.	*1.*	*0.*	*-0.790240*	*-2.037676*	*2.087828*
44.	*1.*	*0.*	*-2.963908*	*-0.749991*	*-1.077834*
45.	*1.*	*0.*	*-4.926381*	*-2.991468*	*0.250448*
46.	*1.*	*0.*	*-4.339793*	*-4.587257*	*-0.263337*
47.	*1.*	*0.*	*-4.947019*	*-4.358585*	*1.387870*
48.	*1.*	*0.*	*-2.398745*	*0.215557*	*1.758698*

Table S5. *Gibbs free energies[a] and equilibrium populations[b] of low-energy conformers of dalesconoside C (**3**)*

*Conformers of **3***	*In MeOH*	
	ΔG[a]	*P (%)[b]*

3-1	*-722993.41758384*	*68.62*
3-2	*-722992.29057588*	*10.22*
3-3	*-722992.72104774*	*21.16*

[a]ΔG, B3LYP/ 6-31g (d, p), in kcal/mol. [b] Boltzmann-population.

Table S6. *Cartesian coordinates for the low-energy reoptimized MMFF conformers of dalesconoside C (**3**) at B3LYP/6-31G(d,p) level of theory in gas*

*Conformer **3-1***		*Standard Orientation (Ångstroms)*			
Center Number	*Atom*	*Type*	*X*	*Y*	*Z*
1.	*6.*	*0.*	*-3.475773*	*1.011675*	*3.348760*
2.	*6.*	*0.*	*-2.628593*	*0.001476*	*3.775755*
3.	*6.*	*0.*	*-1.412598*	*-0.237522*	*3.110880*
4.	*6.*	*0.*	*-1.039534*	*0.553350*	*2.015245*
5.	*6.*	*0.*	*-1.916488*	*1.585183*	*1.599289*
6.	*6.*	*0.*	*-3.124944*	*1.813762*	*2.257142*
7.	*6.*	*0.*	*-0.502338*	*-1.324622*	*3.667936*
8.	*6.*	*0.*	*0.662971*	*-1.670363*	*2.735619*
9.	*6.*	*0.*	*1.290231*	*-0.407590*	*2.145874*
10.	*6.*	*0.*	*0.272604*	*0.359765*	*1.265674*

11.	*8.*	*0.*	*-1.479992*	*2.323758*	*0.534526*
12.	*6.*	*0.*	*-2.314185*	*3.355886*	*0.035894*
13.	*8.*	*0.*	*-0.039934*	*-0.955815*	*4.979075*
14.	*6.*	*0.*	*0.092806*	*-0.306523*	*-0.104455*
15.	*6.*	*0.*	*1.065658*	*-0.118550*	*-1.140891*
16.	*6.*	*0.*	*0.904989*	*-0.778816*	*-2.414661*
17.	*6.*	*0.*	*-0.239920*	*-1.621100*	*-2.615908*
18.	*6.*	*0.*	*-1.158032*	*-1.779398*	*-1.595872*
19.	*6.*	*0.*	*-0.987813*	*-1.128775*	*-0.364909*
20.	*6.*	*0.*	*2.207511*	*0.715624*	*-0.953954*
21.	*6.*	*0.*	*3.136810*	*0.895908*	*-1.946601*
22.	*6.*	*0.*	*2.995512*	*0.256897*	*-3.196914*
23.	*6.*	*0.*	*1.905616*	*-0.556523*	*-3.419802*
24.	*8.*	*0.*	*1.708401*	*-1.208306*	*-4.622093*
25.	*6.*	*0.*	*2.658759*	*-1.044010*	*-5.667359*
26.	*8.*	*0.*	*-0.487716*	*-2.288260*	*-3.772473*
27.	*1.*	*0.*	*0.664689*	*1.365559*	*1.086178*
28.	*1.*	*0.*	*-4.417785*	*1.189466*	*3.859471*
29.	*1.*	*0.*	*-2.889052*	*-0.612767*	*4.632406*
30.	*1.*	*0.*	*-3.791945*	*2.604073*	*1.934792*
31.	*1.*	*0.*	*-1.098467*	*-2.226989*	*3.846188*

32.	1.	0.	1.394643	-2.249624	3.307368
33.	1.	0.	0.302445	-2.310815	1.921538
34.	1.	0.	1.627988	0.248702	2.960533
35.	1.	0.	2.177889	-0.656198	1.557079
36.	1.	0.	-3.277758	2.963160	-0.312287
37.	1.	0.	-1.779011	3.792874	-0.808298
38.	1.	0.	-2.495384	4.132560	0.790236
39.	1.	0.	0.256494	-0.036996	4.931013
40.	1.	0.	-2.015629	-2.418891	-1.775306
41.	1.	0.	-1.741680	-1.272193	0.402264
42.	1.	0.	2.348486	1.221238	-0.007426
43.	1.	0.	3.996333	1.537720	-1.777516
44.	1.	0.	3.742556	0.413823	-3.964178
45.	1.	0.	2.729067	0.004945	-5.976690
46.	1.	0.	3.648267	-1.402505	-5.361999
47.	1.	0.	2.294951	-1.645699	-6.500854
48.	1.	0.	0.228892	-2.087553	-4.398290

Conformer **3-2**		*Standard Orientation (Ångstroms)*			
Center	*Atom*	*Type*	*X*	*Y*	*Z*

Number					
1.	*6.*	*0.*	*-3.478244*	*1.046396*	*3.337214*
2.	*6.*	*0.*	*-2.622711*	*0.051601*	*3.784347*
3.	*6.*	*0.*	*-1.405112*	*-0.188161*	*3.127081*
4.	*6.*	*0.*	*-1.038725*	*0.578283*	*2.013989*
5.	*6.*	*0.*	*-1.922945*	*1.593695*	*1.576595*
6.	*6.*	*0.*	*-3.133292*	*1.827437*	*2.229411*
7.	*6.*	*0.*	*-0.492090*	*-1.261683*	*3.695434*
8.	*6.*	*0.*	*0.636574*	*-1.651064*	*2.737475*
9.	*6.*	*0.*	*1.284919*	*-0.400365*	*2.146274*
10.	*6.*	*0.*	*0.273991*	*0.377150*	*1.267479*
11.	*8.*	*0.*	*-1.491951*	*2.312673*	*0.495565*
12.	*6.*	*0.*	*-2.329413*	*3.334069*	*-0.018032*
13.	*8.*	*0.*	*0.028191*	*-0.729188*	*4.926148*
14.	*6.*	*0.*	*0.092569*	*-0.283865*	*-0.104973*
15.	*6.*	*0.*	*1.062348*	*-0.090894*	*-1.143344*
16.	*6.*	*0.*	*0.900001*	*-0.746920*	*-2.419213*
17.	*6.*	*0.*	*-0.242055*	*-1.593099*	*-2.619550*
18.	*6.*	*0.*	*-1.156136*	*-1.758315*	*-1.597134*
19.	*6.*	*0.*	*-0.985442*	*-1.109751*	*-0.364958*
20.	*6.*	*0.*	*2.203107*	*0.744716*	*-0.955766*

21.	*6.*	*0.*	*3.128571*	*0.932310*	*-1.950547*
22.	*6.*	*0.*	*2.984318*	*0.299502*	*-3.203780*
23.	*6.*	*0.*	*1.896250*	*-0.516200*	*-3.426839*
24.	*8.*	*0.*	*1.696276*	*-1.161783*	*-4.632432*
25.	*6.*	*0.*	*2.642982*	*-0.989915*	*-5.679445*
26.	*8.*	*0.*	*-0.490797*	*-2.258355*	*-3.777645*
27.	*1.*	*0.*	*0.673991*	*1.380788*	*1.093837*
28.	*1.*	*0.*	*-4.420949*	*1.228096*	*3.845181*
29.	*1.*	*0.*	*-2.880675*	*-0.546848*	*4.652853*
30.	*1.*	*0.*	*-3.805672*	*2.606145*	*1.890407*
31.	*1.*	*0.*	*-1.104764*	*-2.150299*	*3.917839*
32.	*1.*	*0.*	*1.372489*	*-2.259582*	*3.279117*
33.	*1.*	*0.*	*0.236775*	*-2.283374*	*1.935413*
34.	*1.*	*0.*	*1.616444*	*0.243006*	*2.969770*
35.	*1.*	*0.*	*2.166600*	*-0.661945*	*1.553879*
36.	*1.*	*0.*	*-3.293087*	*2.933913*	*-0.357877*
37.	*1.*	*0.*	*-1.797186*	*3.758517*	*-0.870558*
38.	*1.*	*0.*	*-2.510903*	*4.122804*	*0.723659*
39.	*1.*	*0.*	*0.551271*	*-1.430667*	*5.338815*
40.	*1.*	*0.*	*-2.011885*	*-2.400491*	*-1.776172*
41.	*1.*	*0.*	*-1.737666*	*-1.255966*	*0.403453*

42.	*1.*	*0.*	*2.345910*	*1.245430*	*-0.006939*
43.	*1.*	*0.*	*3.987041*	*1.575463*	*-1.781138*
44.	*1.*	*0.*	*3.727788*	*0.463243*	*-3.973128*
45.	*1.*	*0.*	*2.709059*	*0.060542*	*-5.984730*
46.	*1.*	*0.*	*3.634709*	*-1.346207*	*-5.378569*
47.	*1.*	*0.*	*2.278974*	*-1.589220*	*-6.514622*
48.	*1.*	*0.*	*0.221549*	*-2.050621*	*-4.405842*

Conformer ***3-3***		*Standard Orientation (Ångstroms)*			
Center Number	*Atom*	*Type*	*X*	*Y*	*Z*
1.	*6.*	*0.*	*-3.435411*	*1.079452*	*3.241217*
2.	*6.*	*0.*	*-2.562841*	*0.195815*	*3.861014*
3.	*6.*	*0.*	*-1.336025*	*-0.128913*	*3.259816*
4.	*6.*	*0.*	*-0.988608*	*0.415607*	*2.012685*
5.	*6.*	*0.*	*-1.867925*	*1.360224*	*1.430630*
6.	*6.*	*0.*	*-3.089300*	*1.678383*	*2.028163*
7.	*6.*	*0.*	*-0.366679*	*-1.036494*	*4.012762*
8.	*6.*	*0.*	*1.075667*	*-0.723887*	*3.612295*
9.	*6.*	*0.*	*1.222447*	*-0.881223*	*2.100469*

10.	*6.*	*0.*	*0.321386*	*0.092671*	*1.293878*
11.	*8.*	*0.*	*-1.421771*	*1.935470*	*0.274317*
12.	*6.*	*0.*	*-2.262777*	*2.855393*	*-0.400947*
13.	*8.*	*0.*	*-0.558921*	*-0.976891*	*5.425108*
14.	*6.*	*0.*	*0.121953*	*-0.463284*	*-0.120588*
15.	*6.*	*0.*	*1.043295*	*-0.178509*	*-1.177848*
16.	*6.*	*0.*	*0.851099*	*-0.770537*	*-2.481983*
17.	*6.*	*0.*	*-0.263898*	*-1.650101*	*-2.683573*
18.	*6.*	*0.*	*-1.123144*	*-1.915749*	*-1.633465*
19.	*6.*	*0.*	*-0.925102*	*-1.329197*	*-0.376409*
20.	*6.*	*0.*	*2.166515*	*0.680318*	*-0.987204*
21.	*6.*	*0.*	*3.044973*	*0.953106*	*-2.003737*
22.	*6.*	*0.*	*2.867764*	*0.388916*	*-3.286043*
23.	*6.*	*0.*	*1.798158*	*-0.449083*	*-3.512770*
24.	*8.*	*0.*	*1.570197*	*-1.033437*	*-4.744369*
25.	*6.*	*0.*	*2.476739*	*-0.784591*	*-5.811302*
26.	*8.*	*0.*	*-0.539106*	*-2.257766*	*-3.867338*
27.	*1.*	*0.*	*0.844368*	*1.051699*	*1.210009*
28.	*1.*	*0.*	*-4.386953*	*1.323334*	*3.705082*
29.	*1.*	*0.*	*-2.807195*	*-0.247986*	*4.819482*
30.	*1.*	*0.*	*-3.763899*	*2.388048*	*1.565078*

31.	*1.*	*0.*	*-0.575409*	*-2.085361*	*3.762029*
32.	*1.*	*0.*	*1.321726*	*0.302167*	*3.922062*
33.	*1.*	*0.*	*1.753562*	*-1.397780*	*4.146465*
34.	*1.*	*0.*	*2.263124*	*-0.746340*	*1.787248*
35.	*1.*	*0.*	*0.956338*	*-1.910517*	*1.830103*
36.	*1.*	*0.*	*-3.211466*	*2.392085*	*-0.699566*
37.	*1.*	*0.*	*-1.715855*	*3.160064*	*-1.294274*
38.	*1.*	*0.*	*-2.473574*	*3.740411*	*0.213397*
39.	*1.*	*0.*	*-0.469632*	*-0.048635*	*5.684712*
40.	*1.*	*0.*	*-1.955614*	*-2.587830*	*-1.812509*
41.	*1.*	*0.*	*-1.628780*	*-1.557187*	*0.419032*
42.	*1.*	*0.*	*2.335288*	*1.127061*	*-0.015678*
43.	*1.*	*0.*	*3.891604*	*1.610689*	*-1.830299*
44.	*1.*	*0.*	*3.573203*	*0.620266*	*-4.073491*
45.	*1.*	*0.*	*2.504535*	*0.280764*	*-6.067064*
46.	*1.*	*0.*	*3.487084*	*-1.126271*	*-5.559433*
47.	*1.*	*0.*	*2.101527*	*-1.353123*	*-6.662886*
48.	*1.*	*0.*	*0.139335*	*-1.990253*	*-4.510273*

Table S7. *Gibbs free energies[a] and equilibrium populations[b] of low-energy conformers of dalesconoside D (**6**)*

*Conformers of **6***	*In MeOH*	
	ΔG [a]	*P (%) [b]*
6-1	*-456272.67724935*	*40.03*
6-2	*-456272.88432765*	*56.80*
6-3	*-456270.32157681*	*0.75*
6-4	*-456271.0174854*	*2.42*

[a]ΔG, B3LYP/ 6-31g (d, p), in kcal/mol. [b] Boltzmann-population.

Table S8. *Cartesian coordinates for the low-energy reoptimized MMFF conformers of dalesconoside D (**6**) at B3LYP/6-31G(d,p) level of theory in gas*

*Conformer **6-1***		*Standard Orientation (Ångstroms)*			
Center Number	*Atom*	*Type*	*X*	*Y*	*Z*
1.	*6.*	*0.*	*-0.065813*	*2.612871*	*-1.066355*
2.	*6.*	*0.*	*-0.233784*	*2.389895*	*0.304128*
3.	*6.*	*0.*	*-0.262242*	*1.084149*	*0.795258*
4.	*6.*	*0.*	*-0.108775*	*-0.023368*	*-0.064686*
5.	*6.*	*0.*	*0.028418*	*0.229798*	*-1.440097*
6.	*6.*	*0.*	*0.058107*	*1.541728*	*-1.936187*
7.	*6.*	*0.*	*-0.125449*	*-1.447317*	*0.487371*
8.	*6.*	*0.*	*0.455894*	*-2.469127*	*-0.503261*

9.	*6.*	*0.*	*-0.127030*	*-2.294499*	*-1.897265*
10.	*6.*	*0.*	*0.104541*	*-0.892117*	*-2.431179*
11.	*8.*	*0.*	*0.285048*	*-0.680156*	*-3.618621*
12.	*8.*	*0.*	*0.673837*	*-1.602664*	*1.659137*
13.	*8.*	*0.*	*-0.448253*	*0.788050*	*2.130625*
14.	*6.*	*0.*	*-0.620403*	*1.856527*	*3.055382*
15.	*8.*	*0.*	*1.858985*	*-2.296583*	*-0.607349*
16.	*1.*	*0.*	*-0.043290*	*3.631760*	*-1.440706*
17.	*1.*	*0.*	*-0.342150*	*3.234640*	*0.973350*
18.	*1.*	*0.*	*0.173934*	*1.679657*	*-3.005148*
19.	*1.*	*0.*	*-1.171000*	*-1.725567*	*0.704152*
20.	*1.*	*0.*	*0.216731*	*-3.471831*	*-0.114859*
21.	*1.*	*0.*	*0.310297*	*-3.009115*	*-2.598060*
22.	*1.*	*0.*	*-1.211616*	*-2.472982*	*-1.871091*
23.	*1.*	*0.*	*0.436445*	*-0.879839*	*2.259634*
24.	*1.*	*0.*	*-0.757243*	*1.388951*	*4.030967*
25.	*1.*	*0.*	*-1.507201*	*2.452345*	*2.811302*
26.	*1.*	*0.*	*0.260548*	*2.508465*	*3.084055*
27.	*1.*	*0.*	*2.161462*	*-2.133670*	*0.300502*

Conformer ***6-2***	*Standard Orientation*

		(Ångstroms)			
Center Number	*Atom*	*Type*	*X*	*Y*	*Z*
1.	*6.*	*0.*	*-0.154420*	*2.496300*	*-1.484487*
2.	*6.*	*0.*	*-0.223707*	*2.492572*	*-0.085574*
3.	*6.*	*0.*	*-0.177805*	*1.279043*	*0.602345*
4.	*6.*	*0.*	*-0.086248*	*0.057649*	*-0.094706*
5.	*6.*	*0.*	*0.018862*	*0.089554*	*-1.493034*
6.	*6.*	*0.*	*-0.026040*	*1.308424*	*-2.187738*
7.	*6.*	*0.*	*-0.083305*	*-1.236989*	*0.692742*
8.	*6.*	*0.*	*-0.384738*	*-2.462637*	*-0.178422*
9.	*6.*	*0.*	*0.457305*	*-2.435743*	*-1.462589*
10.	*6.*	*0.*	*0.216037*	*-1.175781*	*-2.273398*
11.	*8.*	*0.*	*0.212719*	*-1.176134*	*-3.494168*
12.	*8.*	*0.*	*1.204750*	*-1.483553*	*1.287526*
13.	*8.*	*0.*	*-0.199506*	*1.166258*	*1.970398*
14.	*6.*	*0.*	*-0.361254*	*2.347059*	*2.746146*
15.	*8.*	*0.*	*-0.190161*	*-3.648649*	*0.566820*
16.	*1.*	*0.*	*-0.190417*	*3.444120*	*-2.013169*
17.	*1.*	*0.*	*-0.305313*	*3.431679*	*0.448517*
18.	*1.*	*0.*	*0.049680*	*1.286563*	*-3.268929*

19.	*1.*	*0.*	*-0.838212*	*-1.180449*	*1.485892*
20.	*1.*	*0.*	*-1.447472*	*-2.434735*	*-0.448417*
21.	*1.*	*0.*	*1.519454*	*-2.454288*	*-1.181857*
22.	*1.*	*0.*	*0.257772*	*-3.309160*	*-2.086704*
23.	*1.*	*0.*	*1.318477*	*-0.825112*	*1.985864*
24.	*1.*	*0.*	*-0.385690*	*2.022157*	*3.786980*
25.	*1.*	*0.*	*-1.300267*	*2.859102*	*2.504619*
26.	*1.*	*0.*	*0.476245*	*3.040717*	*2.602785*
27.	*1.*	*0.*	*0.623252*	*-3.497966*	*1.074556*

Conformer ***6-3***		*Standard Orientation (Ångstroms)*			
Center Number	*Atom*	*Type*	*X*	*Y*	*Z*
1.	*6.*	*0.*	*0.132700*	*2.872127*	*-1.191548*
2.	*6.*	*0.*	*-0.529047*	*2.715524*	*0.028075*
3.	*6.*	*0.*	*-0.696324*	*1.443640*	*0.580122*
4.	*6.*	*0.*	*-0.207698*	*0.305892*	*-0.082615*

5.	*6.*	*0.*	*0.461444*	*0.478954*	*-1.310143*
6.	*6.*	*0.*	*0.627330*	*1.758025*	*-1.859159*
7.	*6.*	*0.*	*-0.407724*	*-1.063770*	*0.525706*
8.	*6.*	*0.*	*-0.396847*	*-2.158278*	*-0.546434*
9.	*6.*	*0.*	*0.869289*	*-2.054532*	*-1.397355*
10.	*6.*	*0.*	*1.003572*	*-0.692953*	*-2.063717*
11.	*8.*	*0.*	*1.544839*	*-0.568240*	*-3.150863*
12.	*8.*	*0.*	*0.649369*	*-1.302104*	*1.463297*
13.	*8.*	*0.*	*-1.403344*	*1.310894*	*1.754380*
14.	*6.*	*0.*	*-0.607093*	*1.375105*	*2.946661*
15.	*8.*	*0.*	*-0.464630*	*-3.390125*	*0.174323*
16.	*1.*	*0.*	*0.255225*	*3.863857*	*-1.616480*
17.	*1.*	*0.*	*-0.936592*	*3.568931*	*0.561059*
18.	*1.*	*0.*	*1.143546*	*1.843858*	*-2.808955*
19.	*1.*	*0.*	*-1.374683*	*-1.085423*	*1.044221*
20.	*1.*	*0.*	*-1.288253*	*-2.037170*	*-1.181020*
21.	*1.*	*0.*	*1.731887*	*-2.184671*	*-0.731415*
22.	*1.*	*0.*	*0.918289*	*-2.823000*	*-2.175260*
23.	*1.*	*0.*	*0.556596*	*-2.236233*	*1.710686*
24.	*1.*	*0.*	*-1.301473*	*1.263131*	*3.781626*
25.	*1.*	*0.*	*-0.099301*	*2.344902*	*3.027048*

26.	*1.*	*0.*	*0.132141*	*0.568237*	*2.962338*
27.	*1.*	*0.*	*-0.313221*	*-4.117577*	*-0.442577*

Conformer ***6-4***		*Standard Orientation (Ångstroms)*			
Center Number	*Atom*	*Type*	*X*	*Y*	*Z*
1.	*6.*	*0.*	*-0.524687*	*2.716777*	*-0.997376*
2.	*6.*	*0.*	*-0.630755*	*2.467712*	*0.375278*
3.	*6.*	*0.*	*-0.383123*	*1.184822*	*0.874181*
4.	*6.*	*0.*	*-0.026894*	*0.137456*	*-0.000990*
5.	*6.*	*0.*	*0.076546*	*0.413250*	*-1.373975*
6.	*6.*	*0.*	*-0.177341*	*1.700324*	*-1.873119*
7.	*6.*	*0.*	*0.262429*	*-1.239584*	*0.555484*
8.	*6.*	*0.*	*0.009485*	*-2.325246*	*-0.496747*
9.	*6.*	*0.*	*0.802490*	*-2.020200*	*-1.767251*
10.	*6.*	*0.*	*0.450765*	*-0.659381*	*-2.347638*
11.	*8.*	*0.*	*0.489383*	*-0.452052*	*-3.550219*
12.	*8.*	*0.*	*1.629118*	*-1.278494*	*0.969440*
13.	*8.*	*0.*	*-0.474377*	*0.852019*	*2.193418*

14.	*6.*	*0.*	*-0.809197*	*1.863901*	*3.130279*
15.	*8.*	*0.*	*0.420575*	*-3.548625*	*0.116259*
16.	*1.*	*0.*	*-0.718918*	*3.718082*	*-1.370331*
17.	*1.*	*0.*	*-0.904725*	*3.275154*	*1.043447*
18.	*1.*	*0.*	*-0.090282*	*1.865732*	*-2.940735*
19.	*1.*	*0.*	*-0.394288*	*-1.428613*	*1.414223*
20.	*1.*	*0.*	*-1.067947*	*-2.354788*	*-0.722609*
21.	*1.*	*0.*	*1.866209*	*-2.000353*	*-1.498244*
22.	*1.*	*0.*	*0.658461*	*-2.774074*	*-2.547734*
23.	*1.*	*0.*	*1.793897*	*-2.206913*	*1.197705*
24.	*1.*	*0.*	*-0.067397*	*2.672106*	*3.132586*
25.	*1.*	*0.*	*-0.812564*	*1.377487*	*4.106415*
26.	*1.*	*0.*	*-1.802799*	*2.286693*	*2.934021*
27.	*1.*	*0.*	*0.426936*	*-4.242192*	*-0.555768*
